# (*E*)-4-(4-Acrylamidophenoxy)-*N*-Methylpicolinamides as b-Raf/VEGFR-2 Inhibitors with Antiangiogenic Activity in HUVEC and Zebrafish Model

**DOI:** 10.3390/molecules31101757

**Published:** 2026-05-20

**Authors:** Ganga Reddy Velma, Srinivasa Reddy Telukutla, Jayaram Vankudoth, Ajmer Singh Grewal, Steven Privér, Poornachandra Yedla, Ravikumar Akunuri, Donald Wlodkowic, Srihari Pabbaraja, Suresh K. Bhargava, Magdalena Plebanski, Ahmed Kamal

**Affiliations:** 1 Centre for Advanced Materials & Industrial Chemistry (CAMIC), School of Science, RMIT University, GPO BOX 2476, Melbourne, VIC 3001, Australia; velmagangareddy47@gmail.com (G.R.V.);; 2 Academy of Scientific and Innovative Research (AcSIR), Anusandhan Bhawan, 2-Rafi Marg, New Delhi 110 001, India; 3 Department of Organic Synthesis & Process Chemistry, CSIR-Indian Institute of Chemical Technology (IICT), Hyderabad 500 007, India; 4 Accelerator for Translational Research in Clinical Trials (ATRACT) Centre, School of Health and Biomedical Sciences, STEM College, RMIT University, Melbourne, VIC 3083, Australia; 5 Department of Biochemistry, Chemical Synthesis Core Facility, Albert Einstein College of Medicine, Gruss MRRC-314, Bronx, NY 10461, USA; 6 Department of Pharmaceutical Sciences, Guru Gobind Singh College of Pharmacy, Yamuna Nagar 135 001, India; 7 Phenomics Laboratory, School of Science, RMIT University, Plenty Road, P.O. Box 71, Bundoora, VIC 3083, Australia; 8 Department of Chemistry, Osmania University, Hyderabad 500 007, India

**Keywords:** anticancer, b-Raf inhibitors, VEGFR-2 inhibitors, angiogenesis, apoptosis, zebrafish

## Abstract

Pharmacophore hybridization is a well-established strategy for developing novel anticancer agents with improved biological profiles. In this study, a new series of (*E*)-4-(4-acrylamidophenoxy)-*N*-methylpicolinamide derivatives has been rationally designed by hybridizing key structural features of sorafenib with cinnamide pharmacophores and subsequently synthesized. The antiproliferative activities of the synthesized compounds were evaluated against a panel of human cancer cell lines, including A549 (lung), DU-145 (prostate), SKOV3 (ovarian), and HepG2 (liver), along with non-cancerous Hek293T cells. In comparison with the standard drug sorafenib, most of the (*E*)-4-(4-acrylamidophenoxy)-*N*-methylpicolinamides demonstrated significant antiproliferative activity, with specificity toward the HepG2 (liver cancer) cell line, and no effect on the noncancerous cells (Hek293T). Among them, compound **5f**, the derivative containing a trifluoromethyl-substituted cinnamoyl moiety was identified as the lead candidate, exhibiting an IC_50_ of 5.3 µM towards HepG2 (liver) cancer cells, comparable to the reference drug sorafenib. Enzyme inhibition studies showed that compound **5f** inhibited both b-Raf and VEGFR-2 with IC_50_ values of 1.45 and 0.37 µM, respectively. Furthermore, compound **5f** suppressed angiogenesis in vitro and in vivo, as evidenced by the tube formation assay using HUVECs and in transgenic zebrafish Tg(fli1a:EGFP) models, respectively. Mechanistic studies indicated that compound **5f** induced apoptosis in HepG2 cells through mitochondrial membrane depolarization and increased ROS generation. Molecular docking studies supported experimental findings and showed that **5f** can interact with catalytically active residues via hydrogen-bonding interactions. Overall, these results highlight the potential of compound **5f** as a promising dual target therapeutic lead with dual direct anticancer and antiangiogenic properties.

## 1. Introduction

Protein tyrosine kinases represent key therapeutic targets in drug discovery, particularly for the management of proliferative disorders [1,2]. Disruption of normal tyrosine kinase signaling has become a fundamental mechanism enabling cancer cells to bypass physiological regulation of growth, proliferation, and survival [3,4,5].

Sorafenib is a clinically approved small-molecule inhibitor that targets multiple signaling pathways involved in tumor progression [6]. It interferes with receptor tyrosine kinases associated with angiogenesis, including vascular endothelial growth factor receptors (VEGFRs) and platelet-derived growth factor receptor-β (PDGFR-β) [7]. In addition, it inhibits intracellular kinases within the RAF signaling cascade, such as Raf-1 and B-Raf (including mutant forms) [8]. Structurally, sorafenib is a biaryl urea derivative whose planar aromatic framework facilitates interaction with the ATP-binding pocket of kinases, thereby blocking phosphorylation-dependent signaling [8]. Through concurrent inhibition of these pathways, sorafenib suppresses tumor cell proliferation, disrupts angiogenesis, and promotes apoptosis in cancer cells [9,10]. Sorafenib, in view of its unique structural features, demonstrates its broad multi-kinase inhibitory profile and pronounced antiangiogenic activity, achieved through suppression of pro-angiogenic receptor tyrosine kinases (RTKs), including VEGFR [8]. This drug has demonstrated clinical efficacy with an acceptable safety profile in patients with hepatocellular carcinoma and advanced renal cell carcinoma, and has subsequently received approval for its use across multiple cancers [11]. However, the emergence of drug resistance has led to increased toxicity and decreased effectiveness, thereby causing undesirable side effects [12,13]. Consequently, the development of structurally optimized VEGFR-2 inhibitors remains necessary to achieve effective cancer therapies with improved safety profiles and reduced susceptibility to drug resistance [14,15,16].

**Figure 1:**
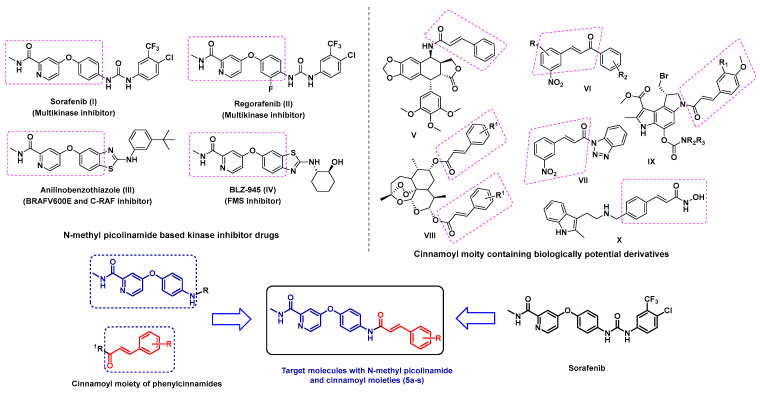
Design strategy for the target molecules (**5a–s**).

In this context, N-methyl-4-phenoxypicolinamide represents one of the most privileged scaffolds with optimized pharmacophore properties, and it is found in diverse multi-kinase inhibitors such as sorafenib (I) and regorafenib (II). In contrast, the 2-anilinobenzothiazole derivative (III) acts as a BRAFV600E and C-RAF inhibitor and BLZ-945 (IV) as an FMS inhibitor Figure 1 [17]. Picolinamide motifs play a key role in protein binding, contributing to multiple cellular and enzymatic activities and exhibiting diverse biological properties such as antidiabetic [18], antifungal [19], and antitumor [6].

Cinnamic acid derivatives, particularly cinnamides V–X (Figure 1) [20,21,22,23,24,25], are an essential class of organic compounds possessing diverse biological properties [26], such as antidiabetic [27], antitubercular [28], TRP channel antagonist [29], antiinflammatory [30], antifungal [31], and antiallergy [32]. Notably, several naturally occurring antitumor agents have been modified by attaching a cinnamoyl moiety to enhance their antitumor efficacy [33]. This approach has led to the synthesis of new libraries of molecules with improved anticancer activity. In recent years, several synthetic cinnamide derivatives of various heterocycles have been reported to possess biological activities, including anticancer activities [34]. These findings indicate that both natural and synthetic cinnamides have immense potential for the development of pharmaceutically important chemical agents. Because of their ease of synthesis and ability to synergize with various bioactive pharmacophores, these compounds have been studied for multiple applications.

Based on analysis of these complementary pharmacophoric features, sorafenib was structurally modified to design a new series of (E)-4-(4-acrylamidophenoxy)-N-methylpicolinamide conjugates (**5a–s**), which were tested against liver cancer cells and compared with the parent molecule, sorafenib. Furthermore, these compounds were evaluated for B-Raf and VEGFR-2 inhibitory activity, as well as for in vitro and in vivo antiangiogenic properties, indicating useful multi-functionalities to support the development of effective cancer therapeutics.

## 2. Results and Discussions

### 2.1. Chemistry

**Scheme 1:**
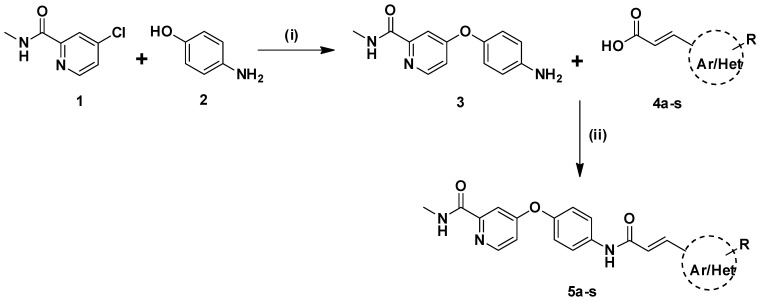
Reagents and conditions: (i) Aromatic amine (**2**), K-*t*OBu, DMF, 80 °C, 4 h; (ii) EDCI, HOBt, DMF, 0 °C to rt, 12 h.

The key precursor 4-(4-aminophenoxy)-*N*-methylpicolinamide (**3**) was prepared according to a previously reported method [35]. Commercially available aromatic and hetero cyclic substituted cinnamic/acrylic acids were used to produce new intermediates **4a–s**. 4-(4-Aminophenoxy)-N-methylpicolinamide (**3**) was synthesized by reacting 4-chloro-N-methylpicolinamide (**1**) with 4-aminophenol (**2**) in DMF at 80 °C for 4 h using potassium tert-butoxide. In the second step, substituted aromatic or heterocyclic acrylic acids (**4a–s**) were coupled with compound **3** via EDCI/HOBt-mediated amide coupling in DMF to afford the desired (E)-4-(4-acrylamidophenoxy)-N-methylpicolinamide derivatives (**5a–s**). The crude products were purified by column chromatography (EtOAc/hexane) to obtain the final compounds in 70–80% yields (Scheme 1). The structures of compounds **5a–s** were confirmed by NMR spectroscopy (^1^H and ^13^C) and high-resolution mass spectrometry (HRMS), with all data consistent with the proposed structures. These compounds are also disclosed in our granted U.S. patent [36].

**Figure 2:**
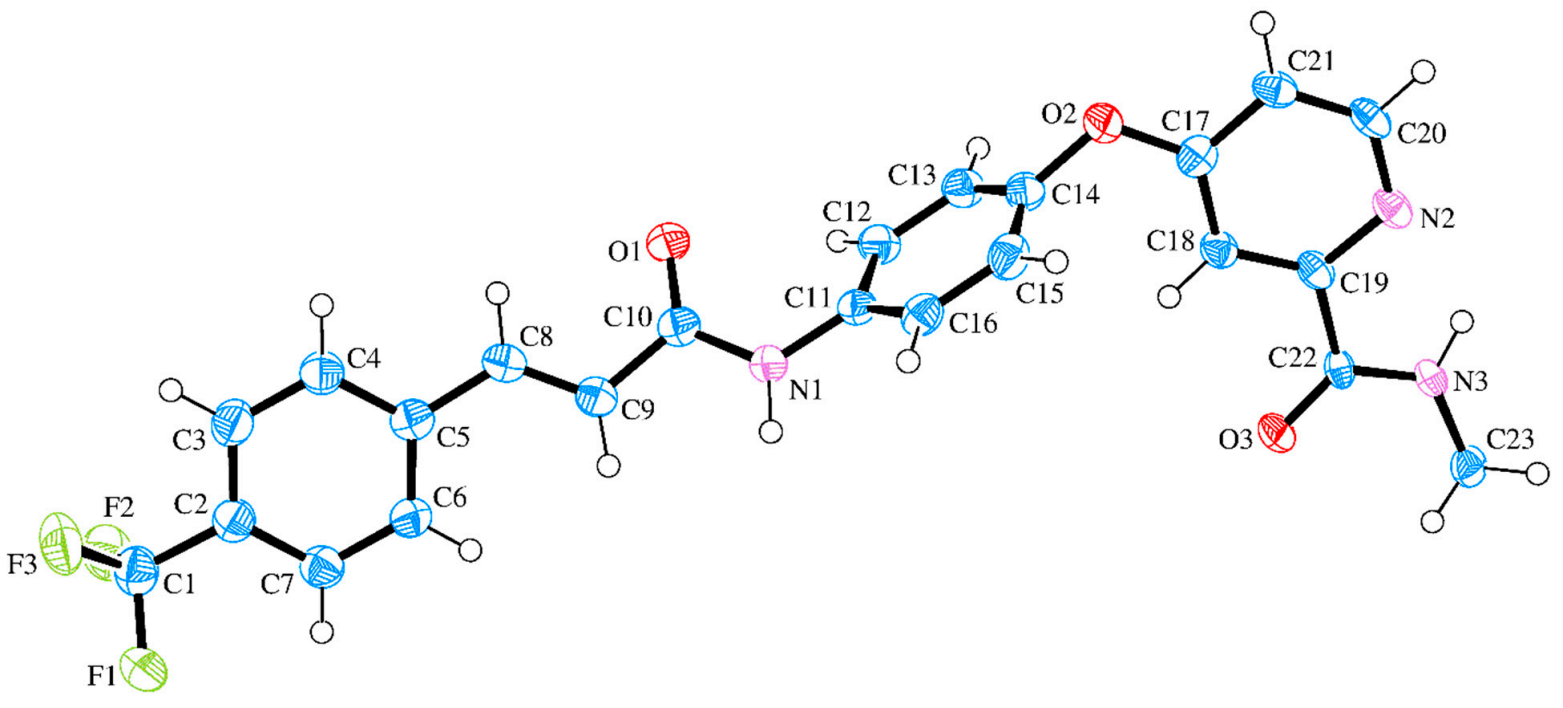
Molecular structure of **5f**. Ellipsoids show 50% probability levels. Crystals were obtained from CH_2_Cl_2_/hexane.

The molecular structure of **5f** was confirmed by single-crystal X-ray diffraction analysis and is shown in Figure 2. The crystal and refinement data are listed in Table S1, and the atomic coordinates and equivalent isotropic displacement parameters, including the selected bond lengths and angles, are provided in Tables S2 and S3 of the Supporting Information. All other bond lengths and angles are within expected ranges and are unremarkable.

### 2.2. In Vitro Cytotoxic Studies

The conjugates of (*E*)-4-(4-acrylamidophenoxy)-*N*-methylpicolinamides (**5a–s)** were evaluated for their cytotoxicity towards a panel of human cancer cell lines, namely lung (A549), ovarian (SKOV3), prostate (DU-145), and liver (HepG2), as well as a noncancerous cell line (Hek293T), employing the MTT assay [37]. and were compared with the positive control drug, sorafenib. As summarized in Table 1, among the four different types of cancer cell lines examined, compounds such as **5c**, **5d**, **5e**, **5f**, **5g**, **5i**, **5j**, **5k**, **5o**, **5p**, **5q** and **5r** exhibited selective and potent activities against HepG2 (liver cancer) cells, with IC_50_ values that range between 5.3 and 8.63 µM, while exhibiting low cytotoxic effects towards the noncancerous Hek293T cells. A number of compounds also showed comparable cytotoxic activity to sorafenib against the SCOV ovarian cancer line (**5e, 5i, 5j, 5n**) without increased toxicity against Hek293T. In addition, these compounds exhibited greater cytotoxicity than the standard drug sorafenib (IC_50_ of 8.74 µM) towards the HepG2 (liver cancer) cell line, particularly **5f** (IC_50_ of 5.3 µM), as shown in Table 1.

Based on the MTT assay results, the structure–activity relationships (SARs) of compounds **5a–s** were examined. Compounds **5c** (4-Cl), **5d** (2-furan), **5f** (4-CF_3_), **5g** (3,4,5-OMe), and **5h** (2,3-OMe) showed moderately sized hydrophobic substituents at the terminal R position, displaying notable growth-inhibitory activity, with HepG2 cells showing greater sensitivity to these analogs than the other tested cell lines. In HepG2 cells, these compounds exhibited IC_50_ values ranging from 5.3 to 9.1 µM, which are comparable to that of sorafenib (7.22 µM). In contrast, derivatives containing small alkyl or weakly hydrophobic groups, such as **5a** (3-F) and **5b** (3-OH, 4-OMe), exhibited weaker activity (IC_50_ = 9.63–24.18 µM), indicating that insufficient hydrophobic surface reduces target engagement. Substituents introducing branching or steric bulk, such as **5e** (4-OMe), showed moderate activity (7.18 µM), suggesting a steric limit in the distal region. Compounds incorporating polar or heteroatom-rich motifs, including **5k** (2,5-OMe) and **5l** (3-indolyl), showed reduced activity (IC_50_ = 7.54–14.71 µM), consistent with the requirement for a predominantly hydrophobic R group. Several additional analogues, such as **5i** (3-OH), **5j** (3,4-F), **5n** (3,4-OMe), and **5p** (4-F), also demonstrated good activity against HepG2 cells (IC_50_ = 6.12–9.16 µM). Notably, nearly all active compounds exhibited minimal cytotoxicity toward non-cancerous HEK-293T cells, with IC_50_ values greater than 50 µM, indicating a favorable selectivity window.

### 2.3. B-Raf^V600E^ and VEGFR-2 Kinase Inhibition

A kinase inhibition assay was performed to investigate the effects of the synthesized compounds (**5a–s**) on the B-Raf^V600E^ and VEGFR-2 enzymes [38]. These compounds exhibited inhibitory activities ranging from the low micromolar to the nanomolar range. As summarized in Table 2, compounds **5a**, **5e**, **5f**, **5i**, **5j**, and **5n** showed inhibitory activity against both enzymes. Notably, compound **5f** inhibited both B-Raf^V600E^ and VEGFR-2, with IC_50_ values of 1.45 μM and 0.37 μM, respectively, while the reference drug sorafenib exhibited IC_50_ values of 0.43 and 0.16 μM, respectively. Compound **5f** showed a stronger inhibition of VEGFR-2 than B-RafV600E, indicating a moderate selectivity toward VEGFR-2. This selectivity may contribute to the antiangiogenic effects, as VEGFR-2 plays a major role in angiogenesis. In contrast, inhibition of B-Raf primarily affects tumour cell proliferation. Therefore, the dual inhibition profile of compound **5f** may enable simultaneous inhibition of tumour angiogenesis and tumour cell growth. Based on the combined B-Raf^V600E^ and VEGFR-2 inhibition profiles, compound **5f** was selected as the lead compound in the series and subsequently used to assess its antiangiogenic and mechanistic properties.

### 2.4. In Vitro Capillary Tube Formation in HUVEC Cells

**Figure 3:**
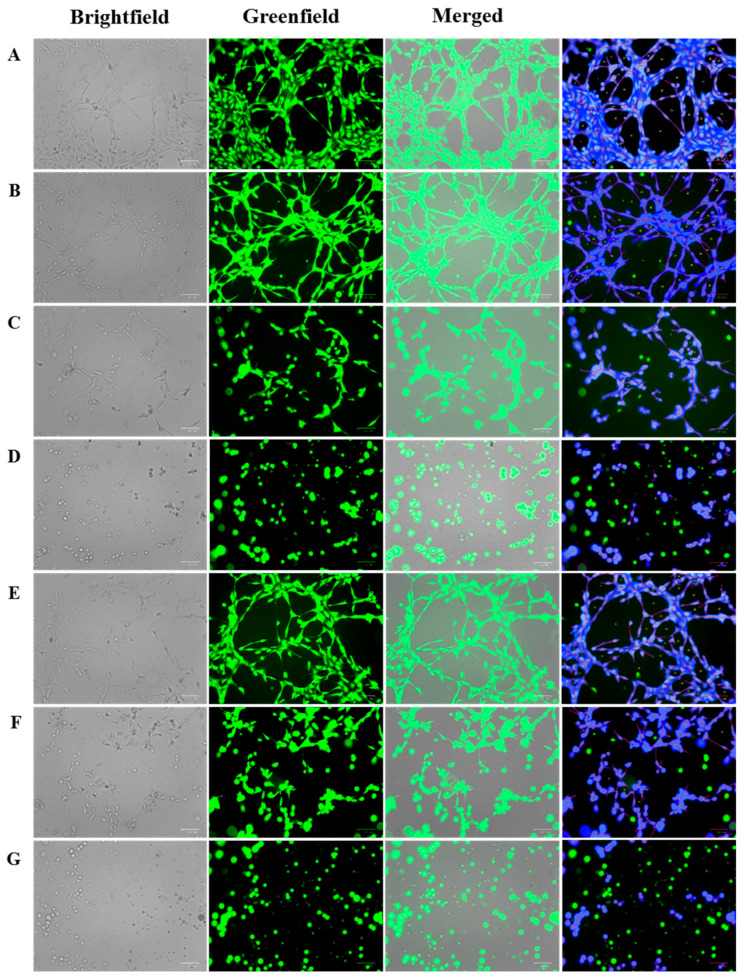
HUVECs capillary tube formation assay. (**A**) Untreated or control HUVECs. (**B**–**D**) HUVECs were treated with **5f** at 1, 2.5, and 5 μM. (**E**–**G**) HUVECs were treated with standard drug sorafenib at 1, 2.5, and 5 μM, respectively. Representative images from three independent experiments are shown.

Angiogenesis plays a crucial role in tumour growth and metastasis, and inhibition of the angiogenesis process represents a therapeutic option for cancer treatment [39]. The capillary tube formation assay is a widely accepted method for assessing angiogenic properties in vitro. Therefore, the antiangiogenic properties of compound **5f** were investigated using the capillary tube formation assay [40]. The primary endothelial cells (HUVECs) were treated with the active compound **5f** or the standard drug sorafenib at 1, 2.5, and 5 μM. Following 18 h of treatment, a concentration-dependent inhibition of capillary tube formation was observed. As shown in Figure 3, the representative images clearly showed the inhibitory effects of compound **5f** after treatment at 5 μM, resulting in near-complete inhibition of tube formation compared to sorafenib.

### 2.5. Angiogenesis Inhibition in Transgenic Zebrafish Model

**Figure 4:**
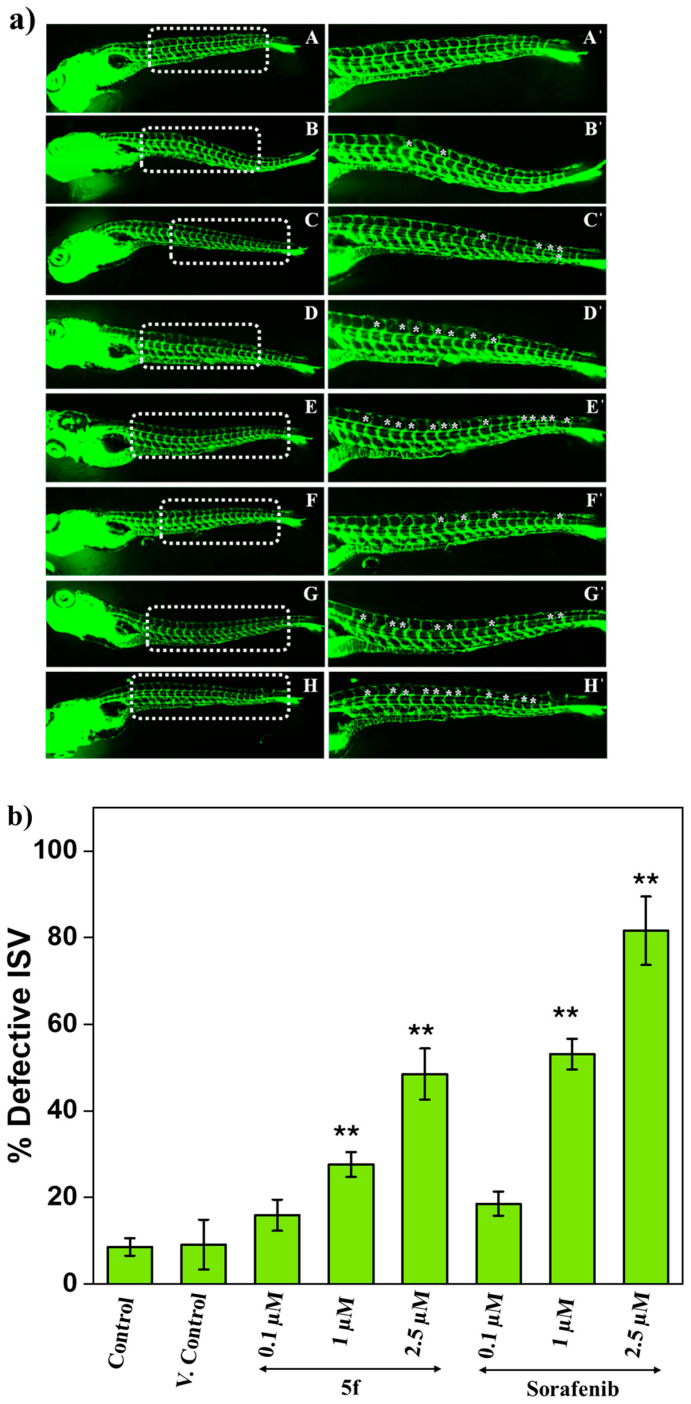
Angiogenesis assay in transgenic Tg(fli1a:EGFP) zebrafish embryos. (**a**) Treatment of embryos with various concentrations of conjugate **5f** or sorafenib (standard drug) at 24 hpf. The zebrafish larvae images at 4 dpf were captured employing a fluorescence microscope in the green channel. (**A**,**A’**) Control embryos. (**B**,**B’**) Vehicle-treated control embryos. (**C**–**E** and **C’**–**E’**) Compound **5f**-treated embryos at 0.1, 1, and 2.5 µM. (**F**–**H** and **F’**–**H’**) Sorafenib-treated embryos at 0.1, 1, and 2.5 µM concentrations. White asterisks showed intersegmental vessels (ISVs). An asterisk is marked for the inhibition of ISV sprouting. Magnified sections of images **A**–**H** are displayed respectively in **A’**–**H’**. Representative images from three independent experiments are shown. (**b**) The percentage of defective intersegmental vessels (ISV) was manually calculated. This bar graph shows quantitative analysis of defective ISVs expressed as the mean ± SD from at least 10 embryos per group. The significance levels were determined using Student’s *t*-test (** *p* < 0.01 vs. control).

Inspired by the promising in vitro antiangiogenic properties of **5f**, an in vivo antiangiogenesis assay was also performed using a transgenic Tg(fil1a:EGFP) zebrafish model, which enables direct visualization of vascular development [41]. Embryos at the 24 hpf (hours post fertilisation) were treated with compound **5f** and sorafenib at 0.1, 1, and 2.5 μM concentrations, with untreated embryos and 0.1% DMSO serving as blank and vehicle controls, respectively [42]. The formation of intersegmental vessels (ISVs) was examined at 4 days post-fertilization (dpf) using a fluorescence microscope. As shown in Figure 4, compound **5f** treatment resulted in concentration-dependent disruption of ISV sprouting compared with control embryos, which exhibited intact, well-organized vascular structures. Quantitative analysis of defective ISVs showed that at 0.1 µM, compound **5f** showed modest inhibition. Increasing the concentration to 1 µM led to a significant (27.9%) reduction in vessel formation, and the percentage of ISV inhibition increased to 48.5% at 2.5 µM. Together, these results demonstrate that compound **5f** effectively inhibits angiogenesis in vivo.

### 2.6. Molecular Docking Studies

Molecular docking provides valuable structural insights into protein-ligand complexes, which are important for structure-based drug design. Docking tools were employed to predict the binding orientations of compound **5f** within the limitations of the protein-binding pocket. The observed cytotoxicity and multi-kinase (VEGFR-2 and B-Raf^V600E^) inhibition profiles of these derivatives, particularly that of conjugate **5f**, prompted us to conduct docking studies and compare their binding modes with those of the reference multi-kinase inhibitor, sorafenib.

**Figure 5:**
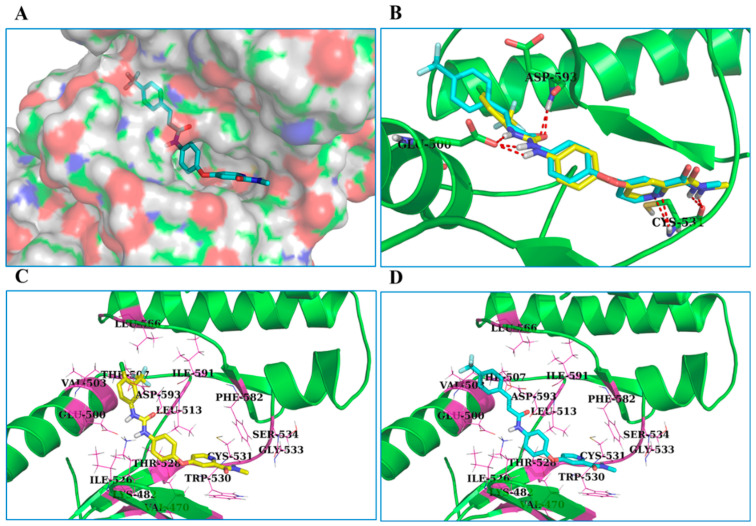
(**A**) Surface binding pose of compound **5f**. (**B**) Binding pose comparison of compound **5f** with sorafenib, along with hydrogen bonding. (**C**,**D**) Docking pose for sorafenib and compound **5f**, respectively (hydrophobic interactions) at the BAX binding pocket of B-Raf^V600E^ (PDB ID: 1UWJ).

**Figure 6:**
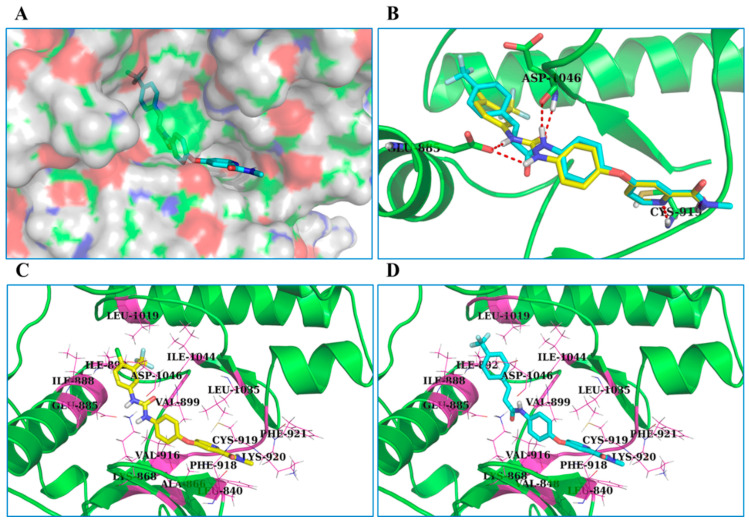
(**A**) Surface binding pose of compound **5f**. (**B**) Binding pose comparison of compound **5f** with sorafenib, along with hydrogen bonding. (**C**,**D**) Docking pose for sorafenib and compound **5f**, respectively (hydrophobic interactions) at the BAX binding pocket of VEGFR-2 (PDB ID: 4AGC).

Compound **5f** and sorafenib were docked into the sorafenib and axitinib binding pockets of B-Raf^V600E^ (PDB ID: 1UWJ) [43] and VEGFR-2 (PDB ID: 4AGC) [44], respectively, and the results indicated that both ligands fit well into the binding pockets of both targets (Figure 5A and Figure 6A). Compound **5f** and sorafenib are linear molecules, as shown in Figure 5 and Figure 6, respectively, and both adopt similar binding poses within the pockets. The diaryl urea and *N*-aryl cinnamamide motifs from sorafenib and compound **5f**, respectively, extend into the hydrophobic pocket. The *N*-methyl picolinamide moiety of both ligands was oriented toward the solvent-exposed region of the binding pocket. Both compound **5f** and sorafenib form multiple hydrogen bonds and hydrophobic interactions with key residues of the B-Raf^V600E^ and VEGFR-2 target proteins. The amino acid residues involved in hydrogen bonding (shown in red) and hydrophobic interactions (shown in blue) with both targets are listed in Table 3 for compound **5f** and sorafenib.

Both ligands showed multiple hydrogen bonding interactions with the target proteins, as shown in Figure 5B and Figure 6B. In the case of B-Raf^V600E^, the N-methyl picolinamide motif present in both ligands interacted with Cys531, whereas the nitrogen atoms in the diaryl urea and *N*-aryl cinnamamide motifs interacted with Glu500 residues. Moreover, the carbonyl oxygen atoms in these motifs interacted with Asp593. Similarly, the *N*-methyl picolinamide motif present in both ligands interacted with the Cys919 residue of VEGFR-2. The nitrogen and carbonyl oxygen atoms present in the diaryl urea portion of sorafenib showed interactions with Glu885 and Asp1046 residues, respectively. At the same time, the nitrogen atom in the **5f** N-aryl cinnamamide group interacted with the Asp1046 residue of VEGFR-2, in addition to several hydrophobic interactions with the target proteins, similar to those observed for sorafenib (Figure 5C,D and Figure 6C,D). The amino acids involved in hydrophobic interactions (residues in blue) with *N*-methyl picolinamide, diaryl urea, and *N*-aryl cinnamamide motifs present in the ligands are depicted in Table 3.

### 2.7. Wound Healing Assay (Cell Migration Assay)

**Figure 7:**
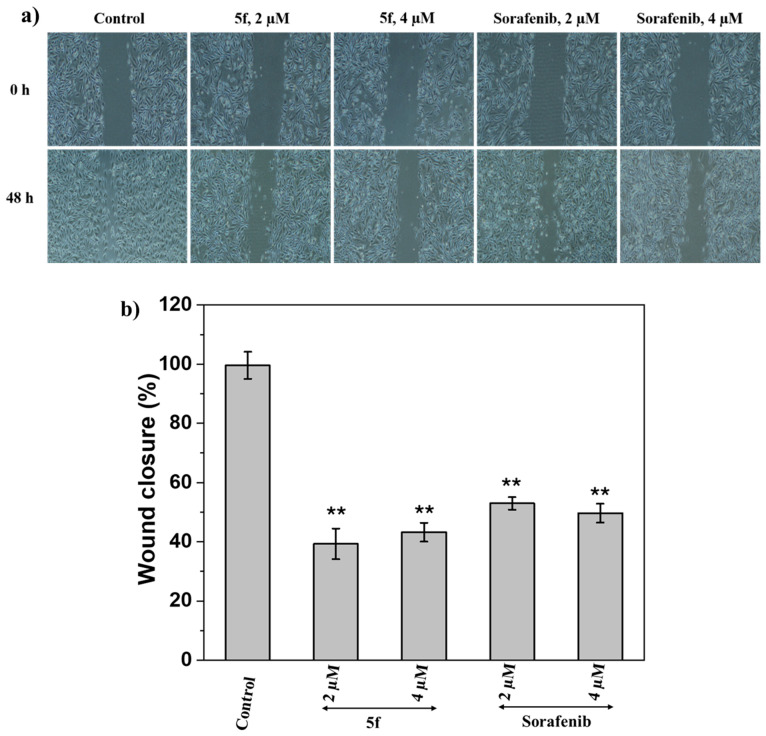
Images of wound healing assay. (**a**) After reaching near confluency, the monolayer was scratched and treated, separately, with 2 and 4 µM concentrations of compound **5f** and sorafenib (standard). Images were captured at 0 and after 48 h. Representative images from three independent experiments are shown. (**b**) The percentage of wound closure was analyzed using ImageJ version 1.53t. Data presented are the mean ± standard deviation (*n* = 3). The significance levels were determined using Student’s *t*-test (** *p* < 0.01 vs. control).

Endothelial cell migration is a critical step in angiogenesis and vascular remodelling. Therefore, to investigate the effect of compound 5f on endothelial cell migration, we performed a linear scratch (wound-healing) assay on HUVEC cells [45]. In this study, cells were treated with 2 and 4 µM concentrations of **5f**, as well as with standard sorafenib, for 48 h and compared with untreated control cells. As shown in Figure 7a,b, untreated HUVEC cells displayed near-complete wound closure (95%) after 48 h, whereas treatment with compound **5f** resulted in markedly reduced wound closure (35–40%) relative to the control, and was comparable to the results for sorafenib.

### 2.8. Transwell Migration (Invasion) Assay

**Figure 8:**
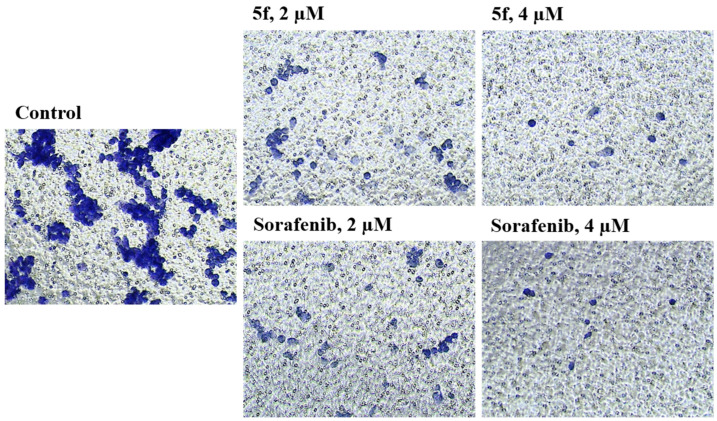
Transwell migration assay: HUVEC cells that invaded through the Matrigel-coated chamber were stained with 0.25 % crystal violet. The photos were captured employing an inverted fluorescence microscope. Representative images from three independent experiments are shown.

Endothelial cell invasion is an essential process during angiogenesis and metastatic progression [37,46]. Here, we performed the Boyden chamber (Transwell) invasion assay in HUVECs to assess the ability of the passage of cancer cells through the membrane barriers and Matrigel in the presence of **5f** (2 and 4 µM), with sorafenib as a standard drug. The majority of cells that migrated to the bottom layer of the Boyden chamber were from the control, whereas in **5f**-treated cells, the number of invaded cells was reduced notably, as shown in Figure 8. These results indicated that compound **5f** effectively suppressed endothelial cell invasion in a concentration-dependent manner (Figure 8).

### 2.9. Apoptosis Studies

#### 2.9.1. Hoechst 33242

**Figure 9:**
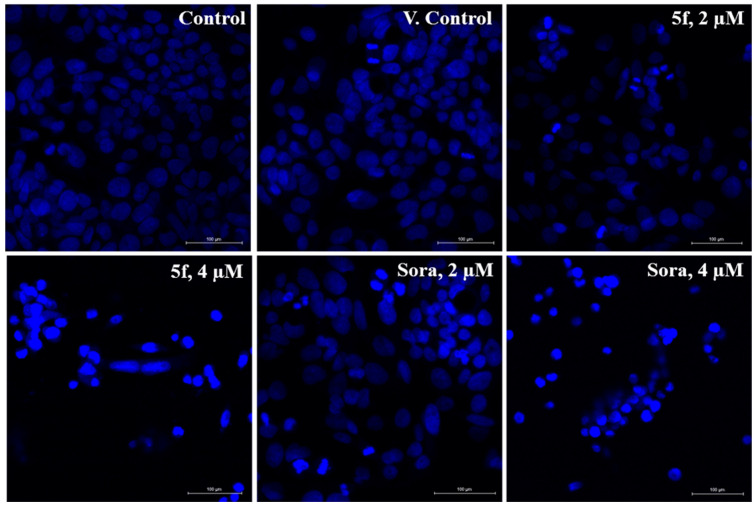
Apoptosis induced by compound **5f** and sorafenib in HepG2 cells, observed by fluorescence microscopy using Hoechst 33242 staining after 48 h. Representative images from three independent experiments are shown. Scale bar represents 100 μm.

Chromatin condensation, DNA fragmentation, and nuclear shrinkage are hallmark morphological features of apoptotic cell death [46]. To investigate whether **5f** induced apoptosis in hepatoma (HepG2) cells, nuclear morphological features were assessed using Hoechst 33242 staining after 24 h of treatment [45]. As shown in Figure 9, untreated control cells showed uniform nuclei with intact morphology, whereas HepG2 cells treated with **5f** displayed pronounced apoptotic features, including chromatin condensation and nuclear shrinkage, in a concentration-dependent manner. The number of cells displaying apoptotic morphology increased markedly at 4 µM, and the damage was comparable to that observed for sorafenib-treated cells at the same concentration.

#### 2.9.2. Measurement of Mitochondrial Membrane Potential (∆Ψm)

**Figure 10:**
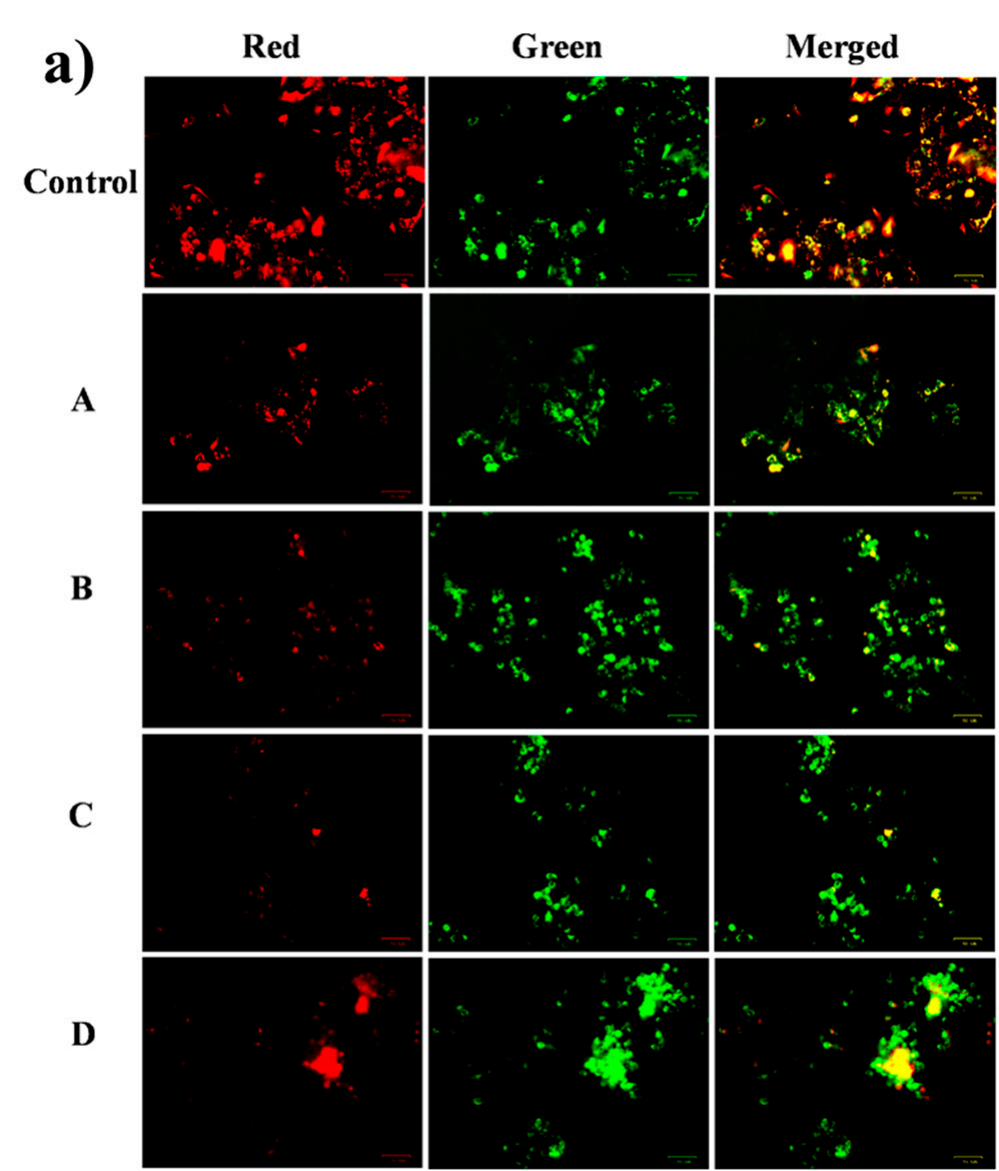

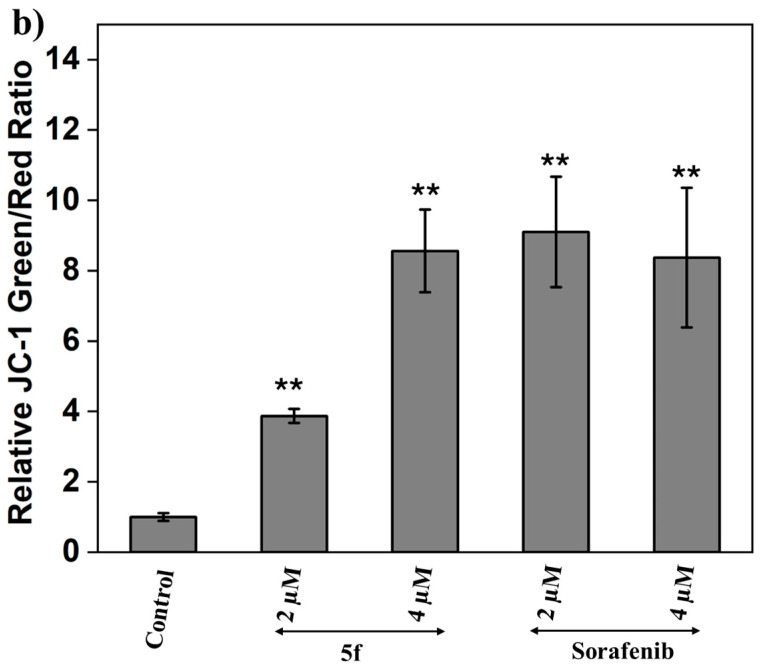
Assessment of mitochondrial membrane potential in HepG2 cells. (**a**) (**A**,**B**) Cells were treated with compound **5f** at 2 μM and 4 μM, respectively, for 24 h. (**C**,**D**) Cells were treated with sorafenib at 2 μM and 4 μM, respectively, for 24 h. Representative images from three independent experiments are shown. (**b**) The relative JC-1 green to red fluorescence ratio was analyzed using ImageJ. Data presented are the mean ± standard deviation (n = 3). The significance levels were determined using Student’s *t*-test (** *p* < 0.01 vs. control).

Mitochondrial membrane potential (ΔΨm) was evaluated using a JC-1 fluorescent probe [47,48]. Cells maintaining intact and polarized mitochondria exhibit red fluorescence due to the formation of JC-1 aggregates. Potent apoptotic inducers exhibit green fluorescence as they lose membrane potential and become deenergized. HepG2 cells separately treated with **5f** and sorafenib exhibited enhanced green fluorescence compared to untreated cells, indicating a loss of mitochondrial membrane potential (Figure 10a). To further validate these findings, the fluorescence intensity was quantitatively analyzed using ImageJ software. Treatment of the HepG2 cells with a 2 µM solution of **5f** resulted in an approximate 4-fold increase in fluorescence intensity, whereas treatment with a 4 µM solution produced a more pronounced effect (approximately 9-fold increase), confirming the compound induces mitochondrial depolarisation. Similar trends were observed for sorafenib-treated cells (Figure 10b).

#### 2.9.3. Measurement of Reactive Oxygen Species (ROS)

**Figure 11:**
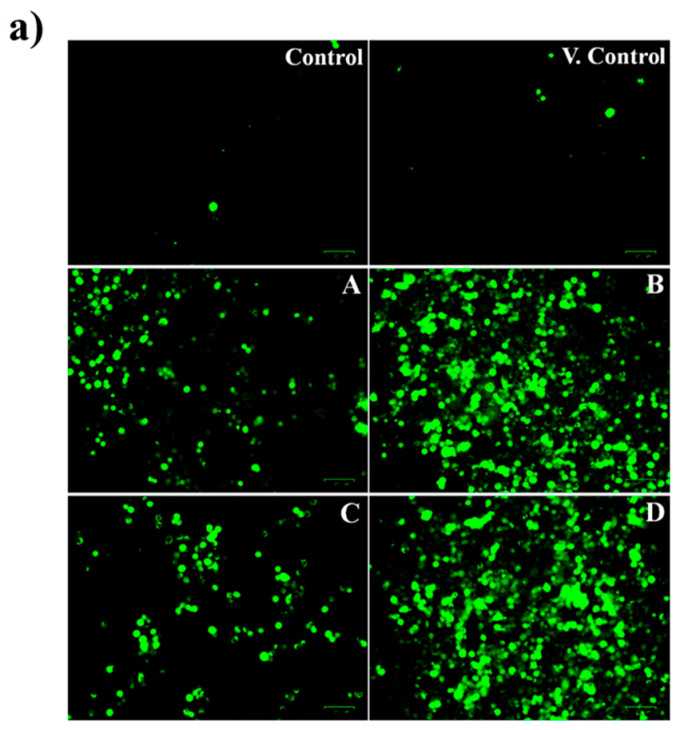

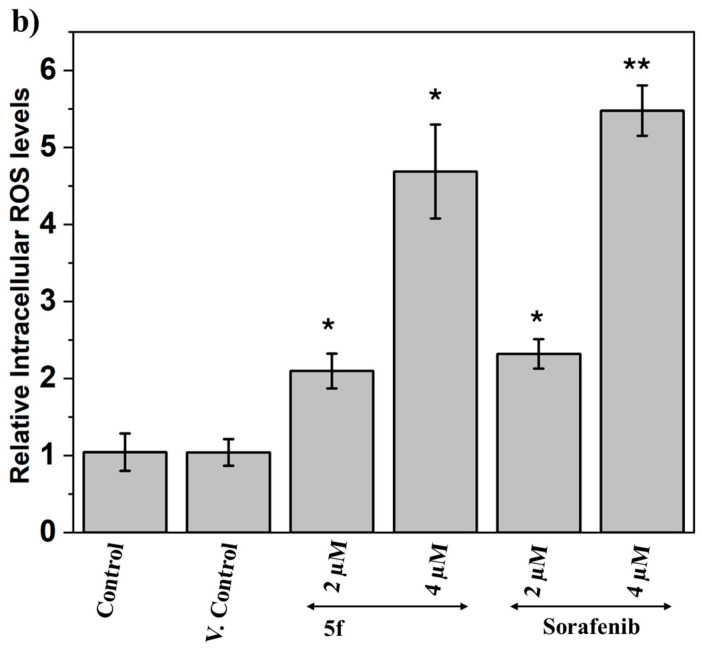
Effects of **5f** and sorafenib on the intracellular ROS levels. (**a**) HepG2 cells were treated with compounds **5f** and sorafenib for 24 h and then stained with carboxy-DCFDA. (**A**) 2 μM of **5f**; (**B**) 4 μM of **5f**; (**C**) 2 μM of sorafenib; (**D**) 4 μM of sorafenib. All the images were captured by a fluorescence microscope. (**b**) The relative green fluorescence was analyzed using ImageJ. Data presented are the mean ± standard deviation (n = 3). The significance levels were determined using Student’s *t*-test (* *p* < 0.05, ** *p* < 0.01 vs. control).

Intracellular ROS accumulation in HepG2 cells following a 24 h treatment with compound **5f** and sorafenib was estimated by carboxy 2′, 7′-dichlorofluorescein diacetate (DCFDA) [37]. It is a non-fluorescent substance and is converted through oxidation by ROS to 2′, 7′-dichlorofluorescein (DCF) inside cells. The ROS production was enhanced in a dose-dependent manner upon treatment with **5f**, comparable to that observed with sorafenib (Figure 11a). Quantitative fluorescence intensity analysis using ImageJ software revealed statistically significant elevation of ROS levels compared with the control group in a dose-dependent manner (Figure 11b).

## 3. Conclusions

In summary, a new series of (*E*)-4-(4-acrylamidophenoxy)-*N*-methylpicolinamide derivatives was designed and synthesized using a pharmacophore hybridization strategy combining structural elements of sorafenib and cinnamide scaffolds. Biological evaluation revealed that several of the synthesized compounds exhibited selective antiproliferative activity against HepG2 liver cancer cells while showing nontoxicity towards non-cancerous Hek293T cells. Structure–activity relationship analysis indicated that the inclusion of lipophilic substituents at the terminal aromatic region is crucial for favorable anticancer activity in this series. Among the compounds synthesized, compound **5f** emerged as the most promising analogue, demonstrating dual inhibitory activity against **B-RafV600E and VEGFR-2 kinases** with greater inhibition towards VEGFR-2. This preferential VEGFR-2 inhibition may contribute to its **antiangiogenic effects,** as supported by the results of the HUVEC tube formation assay and zebrafish angiogenesis model. Mechanistic studies further indicated that compound **5f** induces **apoptosis in HepG2 cells through mitochondrial membrane depolarization and increased intracellular ROS generation.** Compound **5f** demonstrated **comparable antiproliferative activity to sorafenib towards HepG2 cells,** and its enhanced VEGFR-2 selectivity may offer a potential advantage in targeting tumour angiogenesis. Nevertheless, this study has certain limitations, as the antiproliferative activity was evaluated in a **limited panel of cancer cell lines**. Further validation in **additional cancer models and in vivo studies** will be necessary to fully establish the therapeutic potential of this scaffold. Overall, these findings highlight the ***N*-aryl cinnamamide–picolinamide framework** as a promising lead for the development of **dual-target anticancer agents,** capable of simultaneously targeting angiogenesis and tumour cell survival pathways.

## 4. Experimental Section

All chemicals and solvents were obtained commercially from Alfa Aesar (Ward Hill, MA, USA) and Sigma-Aldrich (Burlington, MA, USA) and were used as received unless otherwise noted. Reaction progress was routinely monitored by thin-layer chromatography (TLC) using silica gel plates (Merck, F254, Darmstadt, Germany), and spots were visualized under UV illumination. Product purification was carried out by column chromatography on silica gel (60–120 mesh), employing mixtures of hexane and ethyl acetate as the mobile phase. Melting points of the synthesized compounds were measured using a digital melting point apparatus and are reported without correction. Proton (^1^H) and carbon (^13^C) NMR spectra were recorded on Bruker AVANCE instruments (Billerica, MA, USA) operating at 300, 400, or 500 MHz. Tetramethylsilane (TMS) served as the internal standard. Chemical shifts (δ) are reported in ppm, and coupling constants (J) are expressed in Hz. Signal multiplicities are denoted as singlet (s), doublet (d), triplet (t), quartet (q), doublet of doublets (dd), and multiplet (m). High-resolution mass spectrometric analyses were performed using an electrospray ionization quadrupole time-of-flight (ESI-QTOF) system.

### 4.1. General Procedure for the Synthesis of (E)-4-(4-cinnamamidophenoxy)-N-Methylpicolinamide Derivatives ***(5a–s)***

*Synthesis of 4-(4-aminophenoxy)-N-methylpicolinamide*

Potassium *tert*-butoxide (1.46 g, 13 mmol) was added portionwise to a stirred solution of 4-aminophenol (**2**, 1.09 g, 10 mmol) in anhydrous DMF (30 mL) at room temperature under a nitrogen atmosphere. The reaction mixture was stirred at ambient temperature for 2 h, after which *N*-methyl-4-chloropyridine-2-carboxamide (**1**, 1.71 g, 11 mmol) and potassium carbonate (0.83 g, 6 mmol) were added sequentially. The reaction mixture was heated to 100 °C and maintained at this temperature until completion of the reaction, as monitored by TLC. Upon completion, the mixture was allowed to cool to room temperature and poured into water (300 mL). The aqueous phase was extracted with ethyl acetate (100 mL × 2). The combined organic layers were washed with saturated aqueous sodium carbonate solution and brine, dried over anhydrous sodium sulfate, filtered, and concentrated under reduced pressure to afford the crude product (**3**).

To a solution of aromatic or hetero cyclic substituted acrylic acids (**4a–s**, 1 mmol) in dry dimethylformamide, cooled to 0 °C, were added EDCI (1.2 mmol) and HOBt (1.2 mmol), and the reaction mixture was stirred for 20 min. Subsequently, 4-(4-aminophenoxy)-*N*-methylpicolinamide (**3**, 1 mmol) was added, and the mixture was stirred at room temperature for 12 h. After completion of the reaction, the mixture was carefully quenched by pouring into ice-cold water (25 mL), and the aqueous phase was extracted with ethyl acetate (3 × 50 mL). The combined organic phases were washed with saturated sodium chloride solution. The organic layer was then dried over anhydrous Na_2_SO_4_, filtered, and concentrated in vacuo. The obtained crude compounds were purified by silica gel column chromatography using a gradient elution of ethyl acetate in hexane (0–50%) to afford the desired compounds (**5a–s**) in yields typically ranging from 70% to 80%.

#### 4.1.1. *(E)*-4-(4-(3-(3-fluorophenyl)acrylamido)phenoxy)-*N*-Methylpicolinamide **(5a)**

White solid, yield 71%, Mp: 174–176 °C; ^1^H NMR (500 MHz, CDCl_3_): *δ* 8.39 (d, *J* = 5.49 Hz, 1H), 8.24 (s, 1H), 8.08 (d, *J* = 4.88 Hz, 1H), 7.73–7.68 (m, 3H), 7.65 (d, *J* = 2.44 Hz, 1H), 7.34 (dd, *J* = 7.78, 13.73 Hz, 1H), 7.16 (d, *J* = 9.61 Hz, 1H), 7.07 (dd, *J* = 2.28, 8.39 Hz, 1H), 7.04 (d, *J* = 8.85 Hz, 2H), 6.99 (dd, *J* = 2.44, 5.49 Hz, 1H), 6.58 (d, *J* = 15.41 Hz, 1H), 3.02 (d, *J* = 5.03 Hz, 3H). MS (ESI): *m/z* 392 [M + H]^+^. HRMS (ESI) calcd for C_22_H_19_FN_3_O_3_ [M + H]^+^ 392.14050; found: 392.14102.

#### 4.1.2. *(E)*-4-(4-(3-(4-chlorophenyl)acrylamido)phenoxy)-*N*-Methylpicolinamide **(5c)**

White solid, yield 72%, Mp: 192–195 °C; ^1^H NMR (500 MHz, CDCl_3_): *δ* 8.39 (d, *J* = 5.64 Hz, 1H), 8.15 (s,1H), 8.07 (d, *J* = 4.88 Hz, 1H), 7.72–7.67 (m, 3H), 7.65 (d, *J* = 2.44 Hz, 1H), 7.42 (d, *J* = 8.54 Hz, 2H), 7.34 (d, *J* = 8.39 Hz, 2H), 7.04 (d, *J* = 8.85 Hz, 2H), 6.99 (dd, *J* = 2.44, 5.49 Hz, 1H), 6.56 (d, *J* = 15.41 Hz, 1H), 3.02 (d, *J* = 5.18 Hz, 3H). ^13^C NMR (75 MHz, CDCl_3_+DMSO-*d*_6_): *δ* 166.00, 164.18, 163.81, 151.85, 149.40, 148.93, 139.32, 136.48, 134.88, 133.26, 128.84, 128.68, 122.18, 121.27, 120.79, 113.58, 109.58, 25.76. MS (ESI): *m/z* 408 [M + H]^+^. HRMS (ESI) calcd for C_22_H_19_ClN_3_O_3_ [M + H]^+^ 408.11095; found: 408.11126.

#### 4.1.3. *(E)*-4-(4-(3-(furan-2-yl)acrylamido)phenoxy)-*N*-Methylpicolinamide **(5d)**

White solid, yield 70%, Mp: 163–165 °C; ^1^H NMR (500 MHz, CDCl_3_): *δ* 8.38 (d, *J* = 5.64 Hz, 1H), 8.04 (d, *J* = 4.42 Hz, 1H), 7.88 (s, 1H), 7.69 (d, *J* = 2.28 Hz, 2H), 7.67 (s, 1H), 7.53 (d, *J* = 15.10 Hz, 1H), 7.45 (s, 1H), 7.04 (d, *J* = 8.85 Hz, 2H), 6.96 (dd, *J* = 2.59, 5.64 Hz, 1H), 6.60 (d, *J* = 3.35 Hz, 1H), 6.49 (d, *J* = 14.95 Hz, 1H), 6.47 (d, *J* = 1.83 Hz, 1H), 3.02 (d, *J* = 5.18 Hz, 3H). ^13^C NMR (75 MHz, CDCl_3_+DMSO-*d*_6_): *δ* 165.69, 163.81, 163.60, 151.61, 150.81, 149.11, 148.41, 143.41, 136.44, 127.06, 120.74, 120.43, 119.03, 113.17, 111.64, 109.24, 25.44. MS (ESI): *m/z* 364 [M + H]^+^. HRMS (ESI) calcd for C_20_H_18_N_3_O_4_ [M + H]^+^ 364.12918; found: 364.12948.

#### 4.1.4. *(E)*-4-(4-(3-(4-methoxyphenyl)acrylamido)phenoxy)-*N*-Methylpicolinamide **(5e)**

White solid, yield 78%, Mp: 204–208 °C; ^1^H NMR (300 MHz, CDCl_3_+DMSO-*d*_6_): *δ* 9.77 (s, 1H), 8.39 (d, *J* = 5.66 Hz, 1H), 8.19 (s, 1H), 7.82 (d, *J* = 8.87 Hz, 2H), 7.65 (dd, *J* = 7.17, 9.63 Hz, 2H), 7.52 (d, *J* = 8.49 Hz, 3H), 7.06 (d, *J* = 8.87 Hz, 2H), 6.98–6.90 (m, 3H), 6.66 (d, *J* = 15.67 Hz, 1H), 3.85 (s, 3H), 2.99 (d, *J* = 4.91 Hz, 3H). ^13^C NMR (75 MHz, CDCl_3_+DMSO-*d*_6_): *δ* 165.33, 163.68, 163.46, 159.89, 151.39, 148.91, 148.00, 139.59, 136.28, 128.41, 126.66, 120.43, 120.15, 118.60, 113.39, 112.90, 108.80, 54.38, 25.16. MS (ESI): *m/z* 404 [M + H]^+^. HRMS (ESI) calcd for C_23_H_22_N_3_O_4_ [M + H]^+^ 404.16048; found: 404.16078.

#### 4.1.5. *(E)-N*-methyl-4-(4-(3-(4-(trifluoromethyl)phenyl)acrylamido)phenoxy)picolinamide **(5f)**

White solid, yield 74%, Mp: 210–212 °C; ^1^H NMR (300 MHz, CDCl_3_+DMSO-*d*_6_): *δ* 10.06 (s, 1H), 8.40 (d, *J* = 5.47 Hz, 1H), 8.23 (d, *J* = 4.72 Hz, 1H), 7.83 (d, *J* = 8.87 Hz, 2H), 7.73 (d, *J* = 9.25 Hz, 1H), 7.70–7.63 (m, 5H), 7.08 (d, *J* = 8.87 Hz, 2H), 6.98 (dd, *J* = 2.45, 5.47 Hz, 1H), 6.92 (d, *J* = 15.67 Hz, 1H), 2.99 (d, *J* = 5.09 Hz, 3H). ^13^C NMR (75 MHz, CDCl_3_+DMSO-*d*_6_): *δ* 165.65, 163.84, 163.10, 151.65, 149.17, 148.70, 138.46, 138.03, 136.19, 130.08 (q, *J* = 3.2 Hz), 127.37, 125.11 (q, CF_3_), 124.04, 121.52, 120.97, 120.52, 113.30, 109.21, 25.48. MS (ESI): *m/z* 442 [M + H]^+^. HRMS (ESI) calcd for C_23_H_19_F_3_N_3_O_3_ [M + H]^+^ 442.13730; found: 442.13699.

#### 4.1.6. *(E)*-*N*-methyl-4-(4-(3-(3,4,5-trimethoxyphenyl)acrylamido)phenoxy)picolinamide **(5g)**

White solid, yield 79%, Mp: 148–150 °C; ^1^H NMR (400 MHz, CDCl_3_): *δ* 8.38 (s, 1H), 8.09–8.03 (m, 2H), 7.71–7.65 (m, 4H), 7.05 (d, *J* = 8.92 Hz, 2H), 6.99 (dd, *J* = 2.56, 5.62 Hz, 1H), 6.75 (s, 2H), 6.51 (d, *J* = 15.52 Hz, 1H), 3.89 (s, 3H), 3.87 (s, 6H), 3.02 (d, *J* = 5.13 Hz, 3H). ^13^C NMR (75 MHz, CDCl_3)_: *δ* 166.61, 164.79, 153.63, 153.06, 152.31, 149.86, 137.95, 136.20, 124.31, 121.90, 121.53, 116.69, 114.25, 114.15, 112.56, 110.24, 56.20, 55.93, 26.32. MS (ESI): *m/z* 464 [M + H]^+^. HRMS (ESI) calcd for C_25_H_26_N_3_O_6_ [M + H]^+^ 464.18161; found: 464.18090.

#### 4.1.7. *(E)*-4-(4-(3-(2,3-dimethoxyphenyl)acrylamido)phenoxy)-*N*-Methylpicolinamide **(5h)**

White solid, yield 74%, Mp: 128–131 °C; ^1^H NMR (500 MHz, CDCl_3_): *δ* 8.38 (s, 1H), 8.07–8.01 (m, 2H), 7.89 (s, 1H), 7.71 (s, 1H), 7.70 (d, *J* = 2.59 Hz, 2H), 7.12 (d, *J* = 7.78 Hz, 1H), 7.07–7.03 (m, 3H), 6.96 (dd, *J* = 2.44, 5.49 Hz, 1H), 6.94 (d, *J* = 7.17 Hz, 1H), 6.68 (d, *J* = 15.71 Hz, 1H), 3.89 (s, 3H), 3.87 (s, 3H), 3.02 (d, *J* = 5.03 Hz, 3H). ^13^C NMR (75 MHz, CDCl_3_+DMSO-*d*_6_): *δ* 165.76, 164.03, 163.90, 152.54, 151.69, 149.19, 148.53, 147.58, 136.53, 135.21, 128.44, 123.60, 122.73, 120.88, 120.51, 118.63, 113.27, 112.90, 109.34, 60.47, 55.25, 25.52. MS (ESI): *m/z* 434 [M + H]^+^. HRMS (ESI) calcd for C_24_H_24_N_3_O_5_ [M + H]^+^ 434.17105; found: 434.17054.

#### 4.1.8. *(E)*-4-(4-(3-(3-hydroxyphenyl)acrylamido)phenoxy)-*N*-Methylpicolinamide **(5i)**

White solid, yield 70%, Mp: 180–182 °C; ^1^H NMR (300 MHz, CDCl_3_): *δ* 9.89 (s, 1H), 9.14 (s, 1H), 8.40 (d, *J* = 5.66 Hz, 1H), 8.20 (d, *J* = 4.91 Hz, 1H), 7.82 (d, *J* = 8.87 Hz, 2H), 7.66–7.53 (m, 3H), 7.21 (t, *J* = 7.93 Hz, 1H), 7.07–7.00 (s, 4H), 6.97 (dd, *J* = 2.45, 5.66 Hz, 1H), 6.86 (dd, *J* = 1.51, 7.93 Hz, 1H), 6.75 (d, *J* = 15.48 Hz, 1H), 2.99 (d, *J* = 5.09 Hz, 3H). MS (ESI): *m/z* 390 [M + H]^+^. HRMS (ESI) calcd for C_22_H_20_N_3_O_4_ [M + H]^+^ 390.14483; found: 390.14535.

#### 4.1.9. *(E)*-4-(4-(3-(3,4-difluorophenyl)acrylamido)phenoxy)-*N*-Methylpicolinamide **(5j)**

White solid, yield 76%, Mp: 208–210 °C; ^1^H NMR (400 MHz, CDCl_3_): *δ* 8.40 (d, *J* = 5.62 Hz, 2H), 8.10 (d, *J* = 4.76 Hz, 1H), 7.70 (d, *J* = 8.55 Hz, 2H), 7.64 (d, *J* = 15.89 Hz, 2H), 7.06–7.00 (m, 3H), 6.96 (d, *J* = 6.11 Hz, 2H), 6.84–6.78 (m, 1H), 6.58 (d, *J* = 15.52 Hz, 1H), 3.03 (d, *J* = 5.13 Hz, 3H). ^13^C NMR (75 MHz, CDCl_3)_: *δ* 165.84, 164.03, 163.07, 162.72 (d, *J_CF_* = 247.1 Hz), 162.55 (d, *J_CF_* = 246.8 Hz), 151.79, 149.11 (d, *J_CF_* = 28.8 Hz), 138.0 (d, *J_CF_* = 10.5 Hz), 136.3, 124.3, 121.1 (d, *J_CF_* = 33 Hz), 113.4, 110.0 (dd, *J_CF_* = 25.1, 23.9 Hz), 104.3 (dd, *J_CF_* = 25.5, 25.4 Hz), 103.8, 25.4. MS (ESI): *m/z* 410 [M + H]^+^. HRMS (ESI) calcd for C_22_H_18_F_2_N_3_O_3_ [M + H]^+^ 410.13107; found: 410.13108.

#### 4.1.10. *(E)*-4-(4-(3-(2,5-dimethoxyphenyl)acrylamido)phenoxy)-*N*-Methylpicolinamide **(5k)**

White solid, yield 71%, Mp: 133–135 °C; ^1^H NMR (400 MHz, CDCl_3_): *δ* 8.38 (d, *J* = 5.62 Hz, 1H), 8.03 (d, *J* = 3.66 Hz, 1H), 7.98 (d, *J* = 15.65 Hz, 1H), 7.74 (s, 1H), 7.70 (d, *J* = 2.32 Hz, 2H), 7.68 (s, 1H), 708–7.03 (m, 3H), 6.96 (dd, *J* = 2.56, 5.50 Hz, 1H), 6.89 (d, *J* = 2.93 Hz, 1H), 6.87 (d, *J* = 8.92 Hz, 1H), 6.70 (d, *J* = 15.65 Hz, 1H), 3.86 (s, 3H), 3.79 (s, 3H), 3.02 (d, *J* = 5.13 Hz, 3H). ^13^C NMR (75 MHz, CDCl_3_+DMSO-*d*_6_): *δ* 165.69, 163.84, 163.69, 152.67, 151.62, 149.15, 148.49, 143.68, 140.32, 138.80, 136.38, 130.02, 120.83, 120.73, 120.50, 117.69, 113.27, 109.18, 104.44, 60.12, 55.46, 25.46. MS (ESI): *m/z* 434 [M + H]^+^. HRMS (ESI) calcd for C_24_H_24_N_3_O_5_ [M + H]^+^ 434.17105; found: 434.17069.

#### 4.1.11. *(E)*-*N*-methyl-4-(4-(3-(4-(trifluoromethoxy)phenyl)acrylamido)phenoxy)picolinamide **(5m)**

White solid, yield 76%, Mp: 198–200 °C; ^1^H NMR (300 MHz, CDCl_3_+DMSO-*d*_6_): *δ* 10.01 (s, 1H), 8.39 (d, *J* = 4.34 Hz, 1H), 8.20 (s, 1H), 7.83 (d, *J* = 7.74 Hz, 2H), 7.56–7.40 (m, 4H), 7.23 (d, *J* = 4.53 Hz, 1H), 7.07 (d, *J* = 7.93 Hz, 2H), 6.97 (s, 1H), 6.86 (d, *J* = 15.67 Hz, 1H), 3.01 (d, *J* = 6.04 Hz, 3H). ^13^C NMR (75 MHz, CDCl_3_+DMSO-*d*_6_): *δ* 165.81, 163.97, 163.28, 151.77, 149.26, 148.98, 148.80, 138.63, 136.79, 136.35, 129.90, 126.11, 123.40 (q, OCF_3_), 121.22, 121.03, 120.65, 118.80, 113.41, 109.39, 25.58. MS (ESI): *m/z* 458 [M + H]^+^. HRMS (ESI) calcd for C_23_H_19_F_3_N_3_O_4_ [M + H]^+^ 458.13222; found: 458.13175.

#### 4.1.12. *(E)*-4-(4-(3-(3,4-dimethoxyphenyl)acrylamido)phenoxy)-*N*-Methylpicolinamide **(5n)**

White solid, yield 75%, Mp: 136–138 °C; ^1^H NMR (500 MHz, CDCl_3_): *δ* 8.38 (d, *J* = 5.64 Hz, 1H), 8.05 (d, *J* = 4.88 Hz, 1H), 7.85 (s, 1H), 7.73–7.66 (m, 4H), 7.12 (dd, *J* = 1.83, 8.24 Hz, 1H), 7.06–7.03 m, 3H), 6.98 (dd, *J* = 2.59, 5.64 Hz, 1H), 6.87 (d, *J* = 8.24 Hz, 1H), 6.47 (d, *J* = 15.41 Hz, 1H), 3.92 (s, 3H), 3.90 (s, 3H), 3.02 (d, *J* = 5.18 Hz, 3H). ^13^C NMR (75 MHz, CDCl_3_): *δ* 166.51, 149.77, 149.17, 127.62, 122.33, 121.78, 121.46, 114.28, 111.12, 111.12, 109.78, 77.37, 77.05, 76.73, 56.01, 55.90, 26.25. MS (ESI): *m/z* 434 [M + H]^+^. HRMS (ESI) calcd for C_24_H_24_N_3_O_5_ [M + H]^+^ 434.17105; found: 434.17056.

#### 4.1.13. *(E)*-*N*-methyl-4-(4-(3-(thiophen-2-yl)acrylamido)phenoxy)picolinamide **(5o)**

White solid, yield 74%, Mp: 183–185 °C; ^1^H NMR (500 MHz, CDCl_3_): *δ* 8.38 (d, *J* = 5.49 Hz, 1H), 8.05 (d, *J* = 4.73 Hz, 1H), 7.90 (s, 1H), 7.87 (d, *J* = 15.25 Hz, 1H), 7.68 (dd, *J* = 3.20, 5.79 Hz, 3H), 7.34 (d, *J* = 5.03 Hz, 1H), 7.25 (d, *J* = 3.35 Hz, 1H), 7.07–7.03 (m, 3H), 6.97 (dd, J = 2.59,5.49 Hz, 1H), 6.38 (d, *J* = 15.10 Hz, 1H), 3.02 (d, *J* = 5.18 Hz, 3H). ^13^C NMR (75 MHz, CDCl_3_+DMSO-*d*_6_): *δ* 165.99, 164.13, 163.76, 151.84, 149.35, 148.77, 139.84, 136.61, 133.28, 129.91, 127.72, 126.92, 121.07, 120.73, 120.51, 113.48, 109.59, 25.71. MS (ESI): *m/z* 380 [M + H]^+^. HRMS (ESI) calcd for C_20_H_18_N_3_O_3_S [M + H]^+^ 380.10634; found: 380.10686.

#### 4.1.14. *(E)*-4-(4-(3-(4-fluorophenyl)acrylamido)phenoxy)-*N*-Methylpicolinamide **(5p)**

White solid, yield 77%, Mp: 222–224 °C; 1H NMR (400 MHz, DMSO-*d*_6_): δ 10.42 (s, 1H), 8.78 (dd, *J* = 4.8 Hz, 1H), 8.51 (d, *J* = 5.6 Hz, 1H), 7.84 (d, *J* = 8.9 Hz, 2H), 7.71 (dd, *J* = 8.6, 5.5 Hz, 2H), 7.62 (d, *J* = 15.7 Hz, 1H), 7.39 (d, *J* = 2.6 Hz, 1H), 7.29 (t, *J* = 8.7 Hz, 2H), 7.24–7.19 (m, 2H), 7.15 (dd, *J* = 5.7, 2.6 Hz, 1H), 6.80 (d, *J* = 15.7 Hz, 1H), 2.79 (d, *J* = 4.8 Hz, 3H). ^13^C NMR (101 MHz, DMSO-*D*_6_) δ 165.87, 163.79, 163.53, 162.90 (d, *J_CF_* = 248.7), 152.49, 150.43, 148.55, 139.18, 137.13, 131.35 (d, *J_CF_* = 3.3), 129.99 (d, *J_CF_* = 8.3), 122.00, 121.49, 121.03, 116.05 (d, *J_CF_* = 21.9), 114.07, 108.79, 26.03. MS (ESI): *m/z* 392 [M + H]^+^. HRMS (ESI) calcd for C_22_H_19_FN_3_O_3_ [M + H]^+^ 392.14050; found: 392.14082.

#### 4.1.15. 4-(4-(3-(3,4-dichlorophenyl)ureido)phenoxy)-*N*-Methylpicolinamide **(5q)**

White solid, yield 80%, Mp: 212–216 °C; ^1^H NMR (400 MHz, DMSO-*d*_6_): δ 10.43 (s, 1H), 8.78 (q, *J* = 4.7 Hz, 1H), 8.51 (d, *J* = 5.6 Hz, 1H), 7.93 (d, *J* = 2.0 Hz, 1H), 7.83 (d, *J* = 8.6 Hz, 2H), 7.71 (d, *J* = 8.4 Hz, 1H), 7.67–7.57 (m, 2H), 7.39 (d, *J* = 2.6 Hz, 1H), 7.22 (d, *J* = 8.6 Hz, 2H), 7.15 (dd, *J* = 5.6, 2.5 Hz, 1H), 6.90 (d, *J* = 15.7 Hz, 1H), 2.78 (d, *J* = 4.8 Hz, 3H). ^13^C NMR (101 MHz, DMSO-*d*_6_) δ 165.83, 163.78, 163.10, 152.48, 150.43, 148.67, 137.69, 136.95, 135.65, 131.97, 131.78, 131.18, 129.71, 127.45, 124.45, 121.52, 121.07, 114.09, 108.79, 26.02. MS (ESI): *m/z* 442 [M + H]^+^. HRMS (ESI) calcd for C_22_H_18_Cl_2_N_3_O_3_ [M + H]^+^ 442.0725; found: 442.0719.

#### 4.1.16. 4-(4-(3-(2,4-difluorophenyl)ureido)phenoxy)-*N*-Methylpicolinamide **(5r)**

White solid, yield 72%, Mp: 206–210 °C; ^1^H NMR (400 MHz, DMSO-*d*_6_): δ 10.53 (s, 1H), 8.78 (q, *J* = 4.8 Hz, 1H), 8.50 (d, *J* = 5.6 Hz, 1H), 7.82 (dd, *J* = 16.3, 7.7 Hz, 3H), 7.62 (d, *J* = 15.8 Hz, 1H), 7.43–7.34 (m, 2H), 7.25–7.18 (m, 3H), 7.15 (dd, *J* = 5.6, 2.6 Hz, 1H), 6.94 (d, *J* = 15.9 Hz, 1H), 2.79 (d, *J* = 4.8 Hz, 3H). MS (ESI): m/z 410 [M + H]^+^. ^13^C NMR (101 MHz, DMSO-*D*6) δ 165.85, 163.79, 163.31, 162.87 (dd, *J_CF_* = 251 Hz, 12.7 Hz), 160.89 (dd, *J_CF_* = 254.4, Hz, 13 Hz), 152.49, 150.43, 148.65, 137.03, 132.10, 131.31 (dd, *J_CF_* = 10.2 Hz, 4.5 Hz), 124.68 (d, *J_CF_* = 7 Hz), 121.48, 121.10, 119.21 (dd, *J_CF_* = 11.7 Hz, 3.7 Hz), 114.07, 112.52 (dd, *J_CF_* = 21.7 Hz, 3.4 Hz), 108.80, 105.06, 104.80, 104.54, 26.02. MS (ESI): *m/z* 410 [M + H]^+^. HRMS (ESI) calcd for C_22_H_18_F_2_N_3_O_3_ [M + H]^+^ 410.1316; found: 410.1310.

#### 4.1.17. *N*-methyl-4-(4-(3-(4-nitrophenyl)ureido)phenoxy)picolinamide **(5s)**

Yellow solid, yield 70%, Mp: 240–244 °C; ^1^H NMR (400 MHz, DMSO-*d*_6_): δ 10.71 (s, 1H), 8.78 (q, *J* = 4.8 Hz, 1H), 8.51 (d, *J* = 5.5 Hz, 1H), 8.29 (d, *J* = 8.5 Hz, 2H), 7.91 (d, *J* = 8.5 Hz, 2H), 7.86 (d, *J* = 8.6 Hz, 2H), 7.72 (d, *J* = 15.7 Hz, 1H), 7.39 (d, *J* = 2.5 Hz, 1H), 7.23 (d, *J* = 8.6 Hz, 2H), 7.15 (dd, *J* = 5.6, 2.6 Hz, 1H), 7.08 (d, *J* = 15.8 Hz, 1H), 2.78 (d, *J* = 4.8 Hz, 3H). ^13^C NMR (101 MHz, DMSO-*d*_6_) δ 165.83, 163.79, 162.91, 152.49, 150.44, 148.75, 147.67, 141.31, 137.86, 136.95, 128.83, 126.52, 124.20, 121.52, 121.15, 114.08, 108.82, 26.02. MS (ESI): *m/z* 419 [M + H]^+^. HRMS (ESI) calcd for C_22_H_19_N_4_O_5_ [M + H]^+^ 419.1355; found: 419.1350.

## 5. Biology

### 5.1. Cell Culture

Human cancer cell lines, including lung (A549), prostate (DU-145), ovarian (SKOV3), and liver (HepG2), were procured from ATCC. A549 and DU-145 cells were cultured in RPMI-1640 medium, whereas SKOV3, HepG2, and HEK293T cells were maintained in DMEM. Both the media were supplemented with 10% fetal bovine serum and 1% penicillin–streptomycin. All the cells were cultured in an incubator at 37 °C in a humidified atmosphere containing 5% CO_2_. For subculturing, cells were detached using 0.25% trypsin–EDTA solution. For all the assays, test compounds were dissolved in DMSO to prepare 10 mM stock solutions prior to dilution. All experiments were conducted in triplicate, and results are expressed as mean ± standard deviation. Statistical significance between groups was evaluated using a two-tailed Student’s *t*-test (GraphPad Prism 11), with * *p* < 0.05 and ** *p* < 0.01 considered statistically significant.

### 5.2. MTT Assay

Cytotoxic properties of the newly synthesized sorafenib conjugates were evaluated using the MTT assay on a panel of selected human cancer cell lines (A549, SKOV3, HepG2, and DU145). Cells were seeded at a density of 1.5 × 10^4^ cells in 96-well tissue culture plates. After 24 h, cells were incubated with different concentrations of test samples (**5a–s**) along with the positive control sorafenib and incubated for 48 h. A stock concentration (5mg/mL) of MTT-(3-(4,5-dimethylthiazol-2-yl)-2,5-diphenyltetrazolium bromide, a yellow tetrazole) was prepared in PBS, and 5 μL of MTT was added to each well, followed by 1 h incubation. Later, the medium was removed, and the plates were air-dried overnight at room temperature. Cells without treatment served as a control, and the sorafenib was used as a positive control. DMSO (100 µL) was added to each well, and the absorbance was recorded at 570 nm using a multi-well plate reader (TECAN Infinite 200 PRO, Männedorf, Switzerland). Each experiment was performed in triplicate, and the results are expressed as the mean ± standard deviation [37].

### 5.3. b-Raf Kinase Inhibition Assay

The b-Raf kinase inhibitory effect of the synthesized compounds (**5a–s**) was evaluated using the Z-lyte^TM^ kinase assay kit (Invitrogen, PV3176, Carlsbad, CA, USA) according to the manufacturer’s instructions. The assay is a FRET-based method that measures the differential sensitivity of phosphorylated and non-phosphorylated peptides to proteolytic cleavage. Different concentrations of the test compounds were solubilized in DMSO and transferred to a dose plate. Further, the compounds (2.5 μL/well) were transferred to an assay plate at concentrations ranging from 0.01 to 100 μM for b-Raf activity. Along with test compounds and enzyme, the reaction mixture contains 1X ATP and 2 µM Ser/Thr 3 peptide. After 1 h, 5 µL of development solution was added to each well and incubated at room temperature for 1 h. The reaction was stopped by adding 5 µL of stop reagent. Sorafenib was run in parallel as a positive control. The controls 100% inhibition (no ATP), 0% inhibition (with ATP), and 100% phosphorylation (without ATP and kinase peptide mixture) were used in the experiments. The fluorescence signal was recorded at excitation and emission wavelengths of 400 and 445 nm, respectively. All the experiments were performed in triplicate, and the IC_50_ values were calculated using nonlinear regression analysis.

### 5.4. VEGFR-2 Inhibition Assay

The inhibitory activity of the synthesised compounds against VEGFR-2 kinase was assessed using a luminescence-based assay following the supplier’s protocol (BPS Bioscience). The enzyme VEGFR-2 (KDR) (BPS#40301) and poly(Glu:Tyr) sodium salt substrate were used in combination with Kinase-Glo Plus Luminescence kinase assay kit (Promega#V3772, Promega, Madison, WI, USA). Test compounds at varying concentrations (1 nM to 10 µM) were incubated in a reaction mixture containing Tris buffer (40 mM, pH 7.4), MgCl_2_ (10 mM), BSA (0.1 mg/mL), DTT (1 mM), ATP (10 µM), substrate, and enzyme, for 45 min at 30 °C. After the incubation, Kinase-Glo reagent was added, and the plate was further incubated for 15 min at room temperature in the dark. Luminescence was recorded using a microplate reader (SpectraMax, Molecular Devices, San Jose, CA, USA). Since luminescence intensity correlates inversely with kinase activity, inhibition was quantified accordingly. IC_50_ values were calculated using nonlinear regression analysis of dose–response curves in GraphPad Prism. Each concentration was tested in triplicate [38].

### 5.5. In Vivo Zebrafish Angiogenesis Assay

Transgenic zebrafish embryos [Tg(fli1a:EGFP)] expressing GFP in vascular structures were used to examine the antiangiogenic effects of the synthesised compounds. Embryos were obtained through natural breeding and maintained in E3 at 28.5 °C. Fertilized embryos were sorted and developmentally staged before any experiments, as described earlier. At 24 h post-fertilization, embryos were transferred to 6-well plates (10 embryos per well) containing E3 medium supplemented with compound **5f** (0.1, 1, and 2.5 µM) or sorafenib and incubated for 72 h. After incubation, embryos were anesthetized using tricaine (0.01%), embedded in low-melting agarose, and examined under a fluorescence stereomicroscope (Nikon SMZ18, Nikon, Tokyo, Japan) [42] for the formation of intersegmental vessels (ISVs). The number of intersegmental vessels was calculated manually, and the percentage of ISV inhibition was calculated.

### 5.6. Wound Healing Assay (Migration Assay)

Confluent monolayers of HUVECs cultured in 30 mm Petri dishes were scratched with a 200 μL pipette tip to create uniform wounds (~1 mm wide). Detached cells and debris were removed by washing with PBS, followed by the addition of 2 mL of complete medium (control) or medium supplemented with compound **5f** at concentrations of 2 and 4 μM. Wound closure was monitored by phase-contrast microscopy immediately after scratching and following 30 h of incubation [45].

### 5.7. Transwell Cell Invasion Assay

Cell invasion capacity of HUVEC cells was evaluated using transwell inserts fitted with polycarbonate membranes (8 µm pore size). Serum-starved HUVEC cells were resuspended in low-serum medium (0.1% FBS) and seeded into the upper chamber (2 × 10^4^ cells per well). The lower chamber was filled with medium supplemented with 10% FBS to act as a chemoattractant. Following treatment with compound **5f** and incubation, non-invading cells on the upper side of the membrane surface were removed with a cotton tip and the cells that migrated to the lower surface of the insert were fixed with methanol and stained using crystal violet. Images were captured using an inverted microscope (Nikon) [37].

### 5.8. Nuclear Morphological Analysis

Apoptosis-associated nuclear morphological changes in HepG2 cells were examined using Hoechst 33242 staining. In this assay, A549 cells were grown on coverslips in a 6-well plate and were exposed to compound **5f** for 48 h. Following the treatment, cells were washed with PBS, fixed with 4% paraformaldehyde, and stained with Hoechst dye (2 µg/mL) for 20 min. Excess stain was removed by washing three times with PBS, and nuclear morphology was visualized using a fluorescence imaging system (Bio-Rad ZOE^TM^, Bio-Rad, Hercules, CA, USA) [45].

### 5.9. Assessment of Mitochondrial Membrane Potential

HepG2 cells were seeded in 24-well plates at a density of 5 × 10^5^ cells/mL and treated with compound **5f** (2 and 4 μM) or sorafenib for 24 h. Following treatment, the medium was replaced with 500 μL of fresh medium containing JC-1 dye (5 μg/mL), and the cells were incubated for an additional 20 min. The cells were then washed three times with PBS to remove excess dye and imaged in both red and green fluorescence channels using a ZOE™ Fluorescent Cell Imager (Bio-Rad) [48].

### 5.10. Intracellular Reactive Oxygen Species

The intracellular reactive oxygen species (ROS) levels were determined in HepG2 cells using the DCFDA fluorescence probe. In this assay, cells were treated with increasing concentrations (2 and 4 µM) of compound **5f** or sorafenib for 24 h. After the treatment, cells were incubated with carboxy-DCFDA (10 µM) at 37 °C for 20 min. The intensity of green fluorescence corresponding to ROS generation was measured using a BD Accuri C6 flow cytometer (BD Biosciences, Franklin Lakes, NJ, USA) [37].

### 5.11. Molecular Docking

In silico docking analyses were performed targeting the active sites of B-Raf^V600E^ (PDB ID: 1UWJ) and VEGFR-2 (PDB ID: 4AGC) [43] and VEGFR-2 (PDB ID:4AGC) [44]. The crystal structure coordinates were obtained from the RCSB Protein Data Bank. These proteins were reviewed, excess chains were removed, and the structures were optimized and minimized using the OPLS3e force field with the Protein Preparation Wizard in the Schrödinger program suite. Receptor grids were generated centered on the co-crystal ligand. The number of receptor interaction sites (nsites) = 125, inner grid box size (nx, ny, nz) = 30, grid spacing (bsize) = 1.0, outer docking grid: (nx, ny, nz) = (80, 80, 80). All ligands were built with Maestro Molecule Builder and minimized at pH 7.4 ± 0.5 using the OPLS3e force field. Minimized ligands were docked using flexible ligand extra pression docking in Maestro, version 10.4 of the Schrödinger Suite 2015-4, and the results were visualized in PyMOL [49]. Compound **5f** and sorafenib are shown in stick models colored by atom type: carbon in cyan (compound **5f**) and yellow (sorafenib). Binding interactions were visualized using PyMOL 4.6, with atom types color-coded (hydrogen in white, nitrogen in blue, oxygen in red, chlorine in green, and fluorine in ice blue) appropriately for clarity.

## Figures and Tables

**Table 1:** IC_50_ values (in µM)^a^ for compounds **5a–t** in human cancer cell lines.

Comp.	R	IC_50_ Values (μM) ^a^				
		A549 ^b^	DU145 ^c^	SKOV3 ^d^	HepG2 ^e^	Hek293T ^f^
**5a**	3-F	>25	>25	16.15 ± 2.21	9.63 ± 1.81	>50
**5b**	3-OH, 4-OMe	8.92 ± 1.41	>25	>25	24.18 ± 3.14	>50
**5c**	4-Cl	>25	12.42 ± 2.64	9.23 ± 1.56	6.23 ± 0.34	28.21 ± 3.64
**5d**	2-furyl	>25	>25	>25	7.31 ± 0.61	>50
**5e**	4-OMe	>25	>25	6.4 ± 0.55	7.18 ± 0.42	45.32 ± 5.71
**5f**	4-CF_3_	15.81 ± 1.12	>25	>25	5.3 ± 0.41	39.14 ± 2.83
**5g**	3,4,5-OMe	>25	11.9 ± 0.98	>25	8.21 ± 0.72	>50
**5h**	2,3-OMe	>25	>25	>25	9.11 ± 1.12	>50
**5i**	3-OH	>25	>25	8.27 ± 1.21	7.56 ± 0.61	>50
**5j**	3,4-F	>25	>25	7.34 ± 0.96	8.21 ± 1.14	>50
**5k**	2,5-OMe	>25	19.72 ± 1.42	>25	7.54 ± 0.46	>50
**5l**	3-indolyl	>25	11.45 ± 0.91	>25	14.71 ± 1.81	>50
**5m**	4-OCF_3_	23.2 ± 3.11	>25	17.61 ± 1.48	12.43 ± 2.52	>50
**5n**	3,4-OMe	>25	8.17 ± 1.72	6.12 ± 1.17	14.6 ± 2.1	>50
**5o**	2-thiophenyl	15.87 ± 2.35	7.32 ± 1.41	>25	8.63 ± 1.21	>50
**5p**	4-F	>25	>25	>25	7.61 ± 0.48	>50
**5q**	3,4-Cl	7.21 ± 1.12	16.73 ± 1.26	>25	6.85 ± 0.34	32.84 ± 2.42
**5r**	2,4-F	>25	>25	>25	8.21 ± 0.66	>50
**5s**	4-NO_2_	21.43 ± 2.94	19.4 ± 3.12	>25	9.16 ± 1.21	>50
**3**	**-**	18.5 ± 2.51	>25	>25	21.6 ± 3.41	>50
**Sorafenib**		7.43 ± 0.81	6.02 ± 0.31	7.22 ± 1.11	8.74 ± 0.31	34.61 ± 2.14

^a^ A 50% inhibitory concentration after 72 h of drug treatment, and the values are the average of three individual experiments. ^b^ Lung cancer; ^c^ prostate cancer; ^d^ ovarian cancer; ^e^ liver cancer; and ^f^ human embryonic kidney cell line (noncancerous cell line).

**Table 2:** Kinase inhibitory activities (μM).

Compound	BRAF^V600E^	VEGFR-2
**5a**	4.50 ± 0.26	0.86 ± 0.12
**5b**	8.6 ± 0.19	1.31 ± 0.09
**5c**	5.2 ± 0.24	2.11 ± 0.11
**5d**	5.50 ± 0.31	1.19 ± 0.14
**5e**	3.35 ± 0.18	0.77 ± 0.07
**5f**	1.45 ± 0.22	0.37 ± 0.04
**5g**	6.15 ± 0.42	1.46 ± 0.12
**5h**	9.3 ± 0.15	1.23 ± 0.09
**5i**	2.6 ± 0.32	0.98 ± 0.11
**5j**	4.45 ± 0.24	1.25 ± 0.13
**5k**	5.75 ± 0.18	1.78 ± 0.21
**5l**	9.3 ± 0.16	2.32 ± 0.18
**5m**	7.95 ± 0.24	1.48 ± 0.09
**5n**	1.7 ± 0.12	0.74 ± 0.06
**5o**	9.9 ± 0.34	1.89 ± 0.16
**5p**	9.4 ± 0.44	1.15 ± 0.12
**5q**	8.8 ± 0.52	2.13 ± 0.19
**5r**	10.2 ± 0.18	4.08 ± 0.22
**5s**	6.65 ± 0.26	1.85 ± 0.16
**Sorafenib**	0.43 ± 0.09	0.16 ± 0.02

**Table 3:** Amino acid residues that show hydrogen bonding (residues in red color) and hydrophobic interactions (residues in purple color) with various motifs present in compound **5f** and sorafenib at the BAX binding pocket of B-Raf^V600E^ (PDB ID: 1UWJ) and VEGFR-2 (PDB ID: 4AGC).

	Sorafenib	Compound 5f
**B-RAF^V600E^**	Diaryl urea motif Lys482, Glu500, Val503, Leu504, Thr507, Ile512, Leu513, Ile526, Thr528, Leu566, Ile571, His573, Ile591, Gly592, Asp593, Phe594 and Lys600. *N*-methylpicolinamide motif Ile462, Val470, Ala480, Gln529, Thr530, Cys531, Glu532, Gly533, Ser534 and Phe582.	*N*-aryl cinnamamide motif Lys482, Asn499, Glu500, Val503, Leu504, Leu513, Ile526, Ile571, Ile572, His573, Arg574, Gly592, Asp593, Phe594 and Lys600. *N*-methylpicolinamide motif Ile462, Val470, Ala480, Thr528, Gln529, Trp530, Cys531, Glu532, Gly533, Ser534 and Phe582.
**VGEFR-2**	Diaryl urea motif Val848, Lys868, Glue885, Ile888, Leu889, Ile892, Val898, Val899, Val916, Leu1019, His1026, Ile1044, Cys1045, Asp1046 and Phe1047. *N*-methylpicolinamide motif Leu840, Ala866, Glu917, Phe918, Cys919, Lys920, Phe921, Gly922 and Leu1035.	*N*-aryl cinnamamide motif Lys868, Glu885, Ile888, Leu889, Ile892, Val899, Val916, Leu1019, Cyc1024, Ile1025, His1026, Arg1027, Ile1044, Cys1045, Asp1046 and Phe1047. *N*-methylpicolinamide motif Leu840, Val848, Ala866, Glu917, Phe918, Cys919, Lys920, Phe921, Gly922 and Leu1035.

## Data Availability

Data sharing is not applicable to this article as no new data were created or analyzed in this study.

## Supplementary Materials

The following supporting information can be downloaded at https://www.mdpi.com/article/10.3390/molecules31101757/s1: Table S1: Crystal data and structure refinement for 5f; Table S2: Atomic coordinates (x 104) and equivalent isotropic displacement parameters (Å2x 103) for 5f. U(eq) is defined as one third of the trace of the orthogonalized Uij tensor; Table S3: Bond lengths [Å] and angles [°] for 5f; and ^1^H, ^13^C NMR, MS, and HRMS spectra of the synthesized target compounds.
