# Supplementary material for: (E)-4-(4-Acrylamidophenoxy)-N-Methylpicolinamides as b-Raf/VEGFR-2 Inhibitors with Antiangiogenic Activity in HUVEC and Zebrafish Model
Source: Molecules. 2026 May 20;31(10):1757. doi: 10.3390/molecules31101757 (PMC13209728; doi:10.3390/molecules31101757)
Supplement: Supplementary file 1 [file molecules-31-01757-s001.zip › molecules-4178655-supplementary/molecules-4178655-supplementary materials/Supporting Information.pdf]

Supporting information

(*E*)-4-(4-Acrylamidophenoxy)-*N*-methylpicolinamides as b-Raf/VEGFR-2 Inhibitors with Antiangiogenic Activity in HUVEC and Zebrafish Model

Ganga Reddy Velma,[a,b,c] Srinivasa Reddy Telukutla,*[a,d] Jayaram Vankudoth,[e] Ajmer Singh Grewal,[f] Steven Priver,[a] Y. Poornachandra,[c] Ravikumar Akunuri,[c] Donald Wlodkowic,[g] P. Srihari,[b,c] Suresh K. Bhargava,[a] Magdalena Plebanski,*[d] Ahmed Kamal*[b,c,h]

[a]Centre for Advanced Materials & Industrial Chemistry (CAMIC), School of Science, RMIT University, GPO BOX 2476, Melbourne 3001, Australia

[b]Academy of Scientific and Innovative Research (AcSIR), Anusandhan Bhawan, 2-Rafi Marg, New Delhi 110 001, India

[c]Department of Organic Synthesis & Process Chemistry, CSIR-Indian Institute of Chemical Technology (IICT), Hyderabad 500 007, India

[d]Accelerator for Translational Research in Clinical Trials (ATRACT) Centre, School of Health and Biomedical Sciences, STEM College, RMIT University, Victoria, 3083, Australia

[e]Department of Biochemistry, Chemical Synthesis Core Facility, Albert Einstein College of Medicine, Gruss MRRC-314, Bronx, New York,10461, USA.

[f]Guru Gobind Singh College of Pharmacy, Yamuna Nagar, 135001, Haryana, India

[g]Phenomics Laboratory, School of Science, RMIT University, Plenty Road, P.O. Box 71, Bundoora, Victoria 3083, Australia

[h]Department of Chemistry, Osmania University, Hyderabad500 007, India

Corresponding authors: *e-mail addresses*:

srinivasareddy.telukutla@rmit.edu.au (Dr. Srinivasa Reddy Telukutla)

magdalena.plebanski@rmit.edu.au (Prof. Magdalena Plebanski)

ahmedkamal@iict.res.in; ahmedkamal915@gmail.com (Prof. Ahmed Kamal)

Contents **Page No**

1. Crystal refinement data, selected bond lengths, and bond angles: Table S1-S3 S2-S7
2. ^{1}H NMR and ^{13}C NMR spectra S8-S23
3. LC-MS and HRMS spectra S24-S42

Crystallography

Molecular structure of **5f**. Ellipsoids show 50% probability levels. The thin plate crystals diffracted poorly using both Mo and Cu radiation and, combined with the long b cell dimension, resulted in data with low Friedel pair coverage (73%). Consequently, a meaningful analysis of the Flack parameter could not be achieved and was omitted from the refinement. Carbon-bound hydrogen atoms were included in idealised positions and refined using a riding model. The amide hydrogen atoms were located in the difference Fourier map and their U values set to 1.5 times the equivalent isotropic U of the atom to which they are attached. Crystals were obtained from CH_2Cl_2/hexane.

Table S1. Crystal data and structure refinement for **5f**.

Identification code	**5f**	
Empirical formula	C23 H18 F3 N3 O3	
Moiety formula	C23 H18 F3 N3 O3	
Formula weight	441.40	
Temperature	100(2) K	
Wavelength	0.71073 Å	
Crystal system	Monoclinic	
Space group	C c	
Unit cell dimensions	a = 4.965(7) Å<br>b = 50.69(7) Å<br>c = 8.330(10) Å	α= 90°.<br>β= 105.39(2)°.<br>γ = 90°.
Volume	2021(4) Å^3	
Z	4	
Density (calculated)	1.451 Mg/m^3	
Absorption coefficient	0.116 mm^{-1}	
F(000)	912	
Crystal size	0.401 x 0.208 x 0.044 mm^3	
Theta range for data collection	2.411 to 26.998°.	
Index ranges	-6<=h<=6, -64<=k<=64, -10<=l<=10	
Reflections collected	14299	
Independent reflections	3836 [R(int) = 0.0800]	
Observed reflections	2915	
Completeness to theta = 26.998°	99.9 %	
Absorption correction	Semi-empirical from equivalents	

Max. and min. transmission	0.746 and 0.661	
Refinement method	Full-matrix least-squares on F^2	
Data / restraints / parameters	3836 / 2 / 296	
Goodness-of-fit on F^2	1.039	
Final R indices [I>2sigma(I)]	R1 = 0.0577, wR2 = 0.1324	
R indices (all data)	R1 = 0.0860, wR2 = 0.1457	
Absolute structure parameter	?	
Extinction coefficient	n/a	
Largest diff. peak and hole	0.291 and -0.225 e.Å^{-3}	

Table S2. Atomic coordinates (x 10^4) and equivalent isotropic displacement parameters (Å^{2}x 10^3) for **5f**. U(eq) is defined as one third of the trace of the orthogonalized U^{ij} tensor.

	x	y	z	U(eq)
C(1)	9486(13)	7768(1)	15469(8)	41(1)
C(2)	9075(11)	7892(1)	13771(7)	35(1)
C(3)	10672(12)	7801(1)	12745(7)	40(1)
C(4)	10444(12)	7924(1)	11225(7)	36(1)
C(5)	8633(11)	8137(1)	10711(7)	32(1)
C(6)	7027(11)	8224(1)	11753(7)	35(1)
C(7)	7273(11)	8103(1)	13287(7)	36(1)
C(8)	8582(11)	8273(1)	9140(7)	33(1)
C(9)	6668(11)	8440(1)	8278(7)	32(1)
C(10)	7109(11)	8568(1)	6772(7)	31(1)
C(11)	4685(10)	8791(1)	4155(6)	28(1)
C(12)	6728(11)	8972(1)	3997(7)	33(1)
C(13)	6658(11)	9077(1)	2443(7)	34(1)
C(14)	4594(11)	8996(1)	1072(6)	32(1)
C(15)	2486(12)	8827(1)	1209(7)	36(1)
C(16)	2556(11)	8724(1)	2764(7)	32(1)
C(17)	2971(10)	9263(1)	-1349(7)	29(1)
C(18)	1211(10)	9412(1)	-666(6)	27(1)
C(19)	-414(10)	9602(1)	-1648(6)	26(1)
C(20)	1230(11)	9502(1)	-3881(6)	30(1)
C(21)	2960(10)	9309(1)	-3001(6)	30(1)
C(22)	-2316(10)	9770(1)	-915(6)	25(1)
C(23)	-5582(10)	10144(1)	-1452(6)	31(1)

F(1)	7210(8)	7792(1)	16027(5)	61(1)
F(2)	11567(7)	7881(1)	16625(4)	55(1)
F(3)	10086(8)	7511(1)	15488(5)	57(1)
N(1)	4756(9)	8673(1)	5722(5)	30(1)
N(2)	-472(9)	9650(1)	-3251(5)	30(1)
N(3)	-3567(8)	9969(1)	-1878(5)	28(1)
O(1)	9423(8)	8583(1)	6508(5)	39(1)
O(2)	4785(8)	9075(1)	-509(5)	37(1)
O(3)	-2642(7)	9716(1)	476(4)	31(1)

Table S3. Bond lengths [Å] and angles [°] for **5f**.

C(1)-F(3)	1.335(7)	C(1)-F(1)	1.337(7)
C(1)-F(2)	1.340(7)	C(1)-C(2)	1.512(8)
C(2)-C(7)	1.382(8)	C(2)-C(3)	1.390(8)
C(3)-C(4)	1.387(8)	C(3)-H(3)	0.9500
C(4)-C(5)	1.400(8)	C(4)-H(4)	0.9500
C(5)-C(6)	1.396(7)	C(5)-C(8)	1.472(8)
C(6)-C(7)	1.393(8)	C(6)-H(6)	0.9500
C(7)-H(7)	0.9500	C(8)-C(9)	1.331(7)
C(8)-H(8)	0.9500	C(9)-C(10)	1.481(7)
C(9)-H(9)	0.9500	C(10)-O(1)	1.228(6)
C(10)-N(1)	1.366(7)	C(11)-C(16)	1.388(7)
C(11)-C(12)	1.398(7)	C(11)-N(1)	1.428(7)
C(12)-C(13)	1.392(8)	C(12)-H(12)	0.9500
C(13)-C(14)	1.378(8)	C(13)-H(13)	0.9500
C(14)-C(15)	1.382(8)	C(14)-O(2)	1.403(6)
C(15)-C(16)	1.388(8)	C(15)-H(15)	0.9500
C(16)-H(16)	0.9500	C(17)-O(2)	1.369(6)
C(17)-C(18)	1.385(7)	C(17)-C(21)	1.395(7)
C(18)-C(19)	1.377(7)	C(18)-H(18)	0.9500
C(19)-N(2)	1.350(6)	C(19)-C(22)	1.515(7)
C(20)-N(2)	1.340(6)	C(20)-C(21)	1.376(7)
C(20)-H(20)	0.9500	C(21)-H(21)	0.9500
C(22)-O(3)	1.242(6)	C(22)-N(3)	1.337(6)

C(23)-N(3)	1.449(6)	C(23)-H(23A)	0.9800
C(23)-H(23B)	0.9800	C(23)-H(23C)	0.9800
N(1)-H(1N)	0.99(6)	N(3)-H(3N)	0.89(6)
		F(3)-C(1)-F(1)	107.0(5)
F(3)-C(1)-F(2)	106.4(5)	F(1)-C(1)-F(2)	105.8(5)
F(3)-C(1)-C(2)	113.1(5)	F(1)-C(1)-C(2)	111.7(5)
F(2)-C(1)-C(2)	112.4(5)	C(7)-C(2)-C(3)	120.5(5)
C(7)-C(2)-C(1)	120.7(5)	C(3)-C(2)-C(1)	118.6(5)
C(4)-C(3)-C(2)	119.4(5)	C(4)-C(3)-H(3)	120.3
C(2)-C(3)-H(3)	120.3	C(3)-C(4)-C(5)	121.0(5)
C(3)-C(4)-H(4)	119.5	C(5)-C(4)-H(4)	119.5
C(6)-C(5)-C(4)	118.6(5)	C(6)-C(5)-C(8)	121.9(5)
C(4)-C(5)-C(8)	119.4(5)	C(7)-C(6)-C(5)	120.4(5)
C(7)-C(6)-H(6)	119.8	C(5)-C(6)-H(6)	119.8
C(2)-C(7)-C(6)	120.0(5)	C(2)-C(7)-H(7)	120.0
C(6)-C(7)-H(7)	120.0	C(9)-C(8)-C(5)	128.1(5)
C(9)-C(8)-H(8)	115.9	C(5)-C(8)-H(8)	115.9
C(8)-C(9)-C(10)	119.5(5)	C(8)-C(9)-H(9)	120.3
C(10)-C(9)-H(9)	120.3	O(1)-C(10)-N(1)	123.0(5)
O(1)-C(10)-C(9)	122.2(5)	N(1)-C(10)-C(9)	114.8(4)
C(16)-C(11)-C(12)	119.5(5)	C(16)-C(11)-N(1)	119.2(5)
C(12)-C(11)-N(1)	121.3(5)	C(13)-C(12)-C(11)	119.9(5)
C(13)-C(12)-H(12)	120.0	C(11)-C(12)-H(12)	120.0
C(14)-C(13)-C(12)	119.2(5)	C(14)-C(13)-H(13)	120.4

C(12)-C(13)-H(13)	120.4	C(13)-C(14)-C(15)	121.9(5)
C(13)-C(14)-O(2)	118.3(5)	C(15)-C(14)-O(2)	119.7(5)
C(14)-C(15)-C(16)	118.6(5)	C(14)-C(15)-H(15)	120.7
C(16)-C(15)-H(15)	120.7	C(11)-C(16)-C(15)	120.8(5)
C(11)-C(16)-H(16)	119.6	C(15)-C(16)-H(16)	119.6
O(2)-C(17)-C(18)	124.8(5)	O(2)-C(17)-C(21)	116.8(4)
C(18)-C(17)-C(21)	118.5(5)	C(19)-C(18)-C(17)	118.1(5)
C(19)-C(18)-H(18)	121.0	C(17)-C(18)-H(18)	121.0
N(2)-C(19)-C(18)	124.8(5)	N(2)-C(19)-C(22)	116.3(4)
C(18)-C(19)-C(22)	118.9(4)	N(2)-C(20)-C(21)	124.1(4)
N(2)-C(20)-H(20)	118.0	C(21)-C(20)-H(20)	118.0
C(20)-C(21)-C(17)	118.9(5)	C(20)-C(21)-H(21)	120.6
C(17)-C(21)-H(21)	120.6	O(3)-C(22)-N(3)	124.8(4)
O(3)-C(22)-C(19)	120.1(4)	N(3)-C(22)-C(19)	115.1(4)
N(3)-C(23)-H(23A)	109.5	N(3)-C(23)-H(23B)	109.5
H(23A)-C(23)-H(23B)	109.5	N(3)-C(23)-H(23C)	109.5
H(23A)-C(23)-H(23C)	109.5	H(23B)-C(23)-H(23C)	109.5
C(10)-N(1)-C(11)	123.9(4)	C(10)-N(1)-H(1N)	118(4)
C(11)-N(1)-H(1N)	117(4)	C(20)-N(2)-C(19)	115.7(4)
C(22)-N(3)-C(23)	123.8(4)	C(22)-N(3)-H(3N)	110(4)
C(23)-N(3)-H(3N)	126(4)	C(17)-O(2)-C(14)	118.5(4)

NMR spectra

Compound **5a**

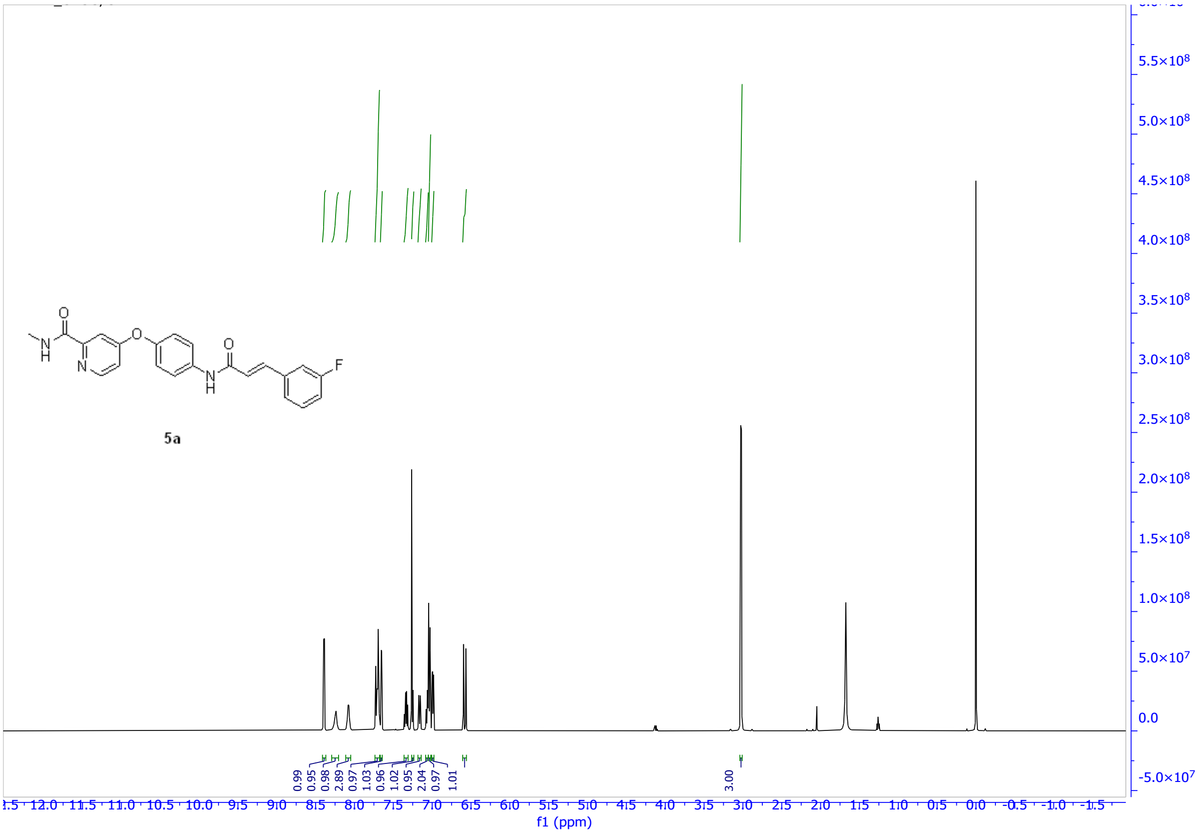

Compound **5c**

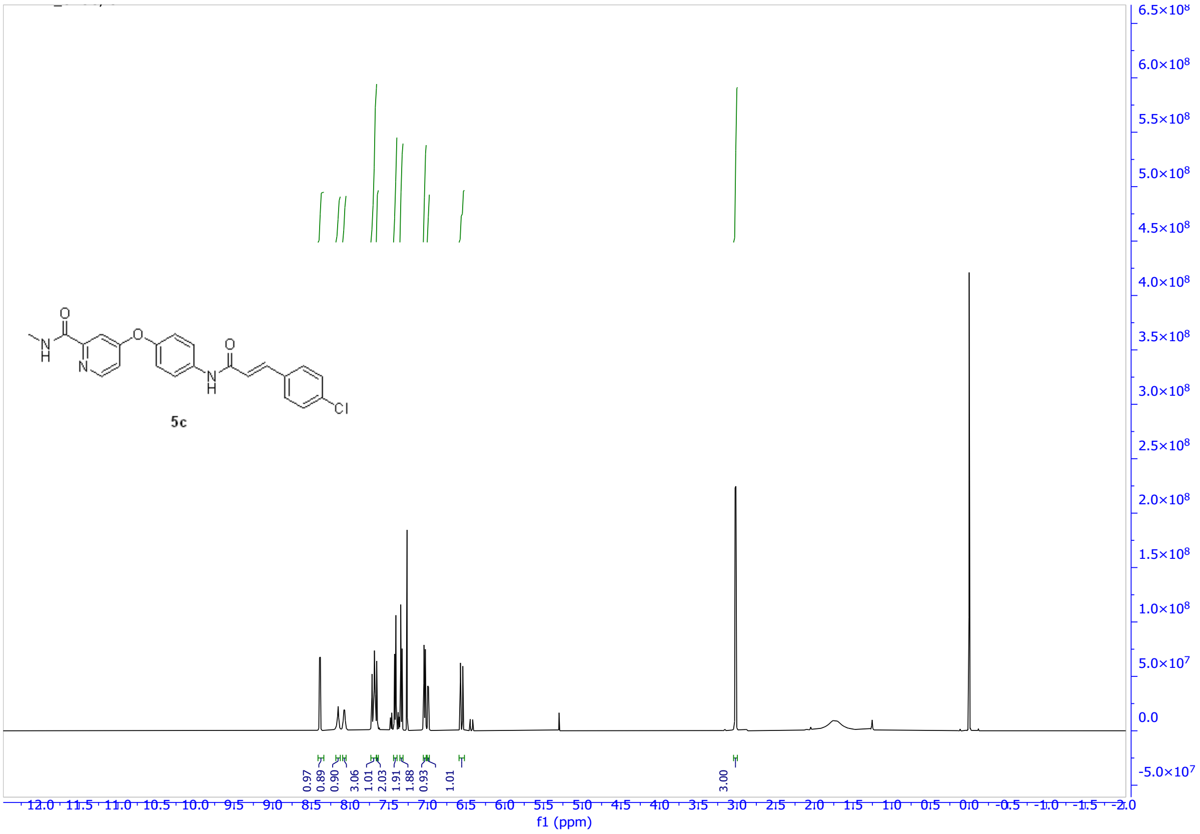

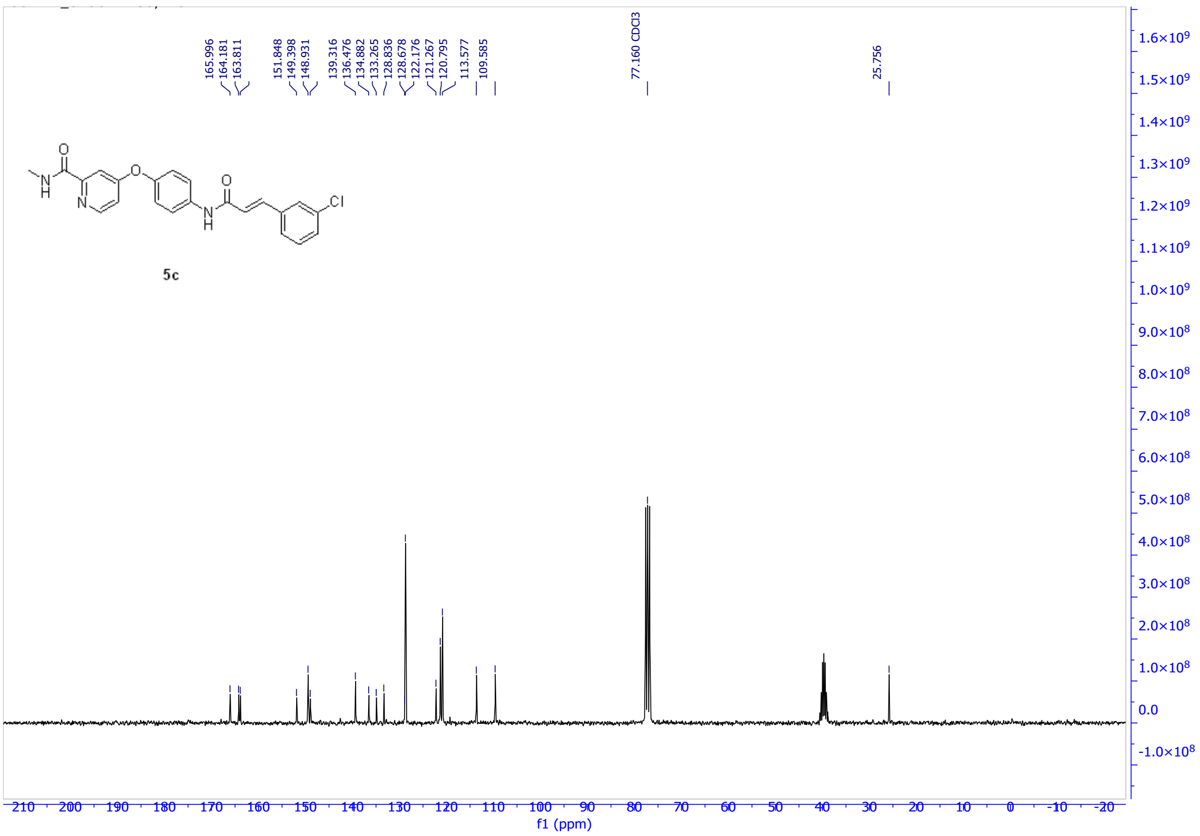
165.996
164.181
163.811
151.848
149.398
148.931
139.316
136.476
134.882
133.265
128.836
128.678
122.176
121.267
120.795
113.577
109.585
77.160 CDCl3
25.756
5c
f1 (ppm)

Compound **5d**

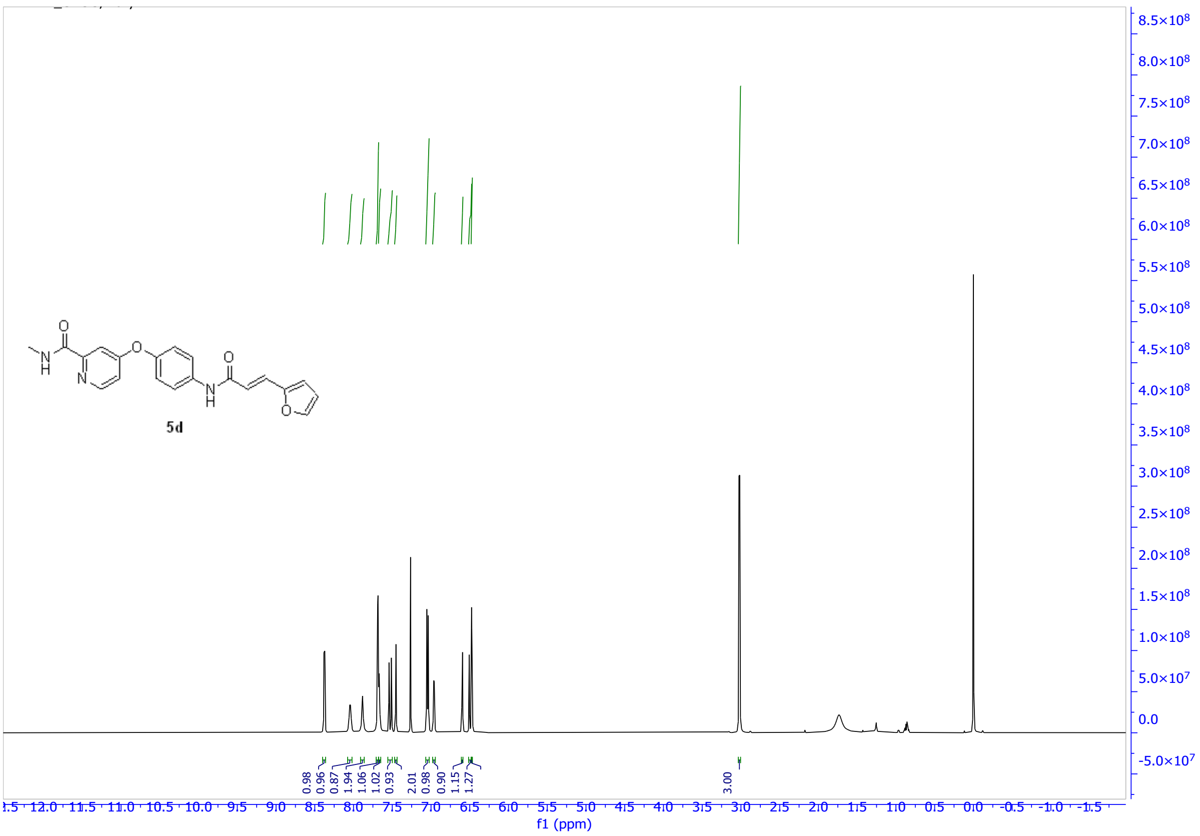
5d
0.98
0.96
0.87
1.94
1.06
1.02
0.93
2.01
0.98
0.90
1.15
1.27
3.00
f1 (ppm)

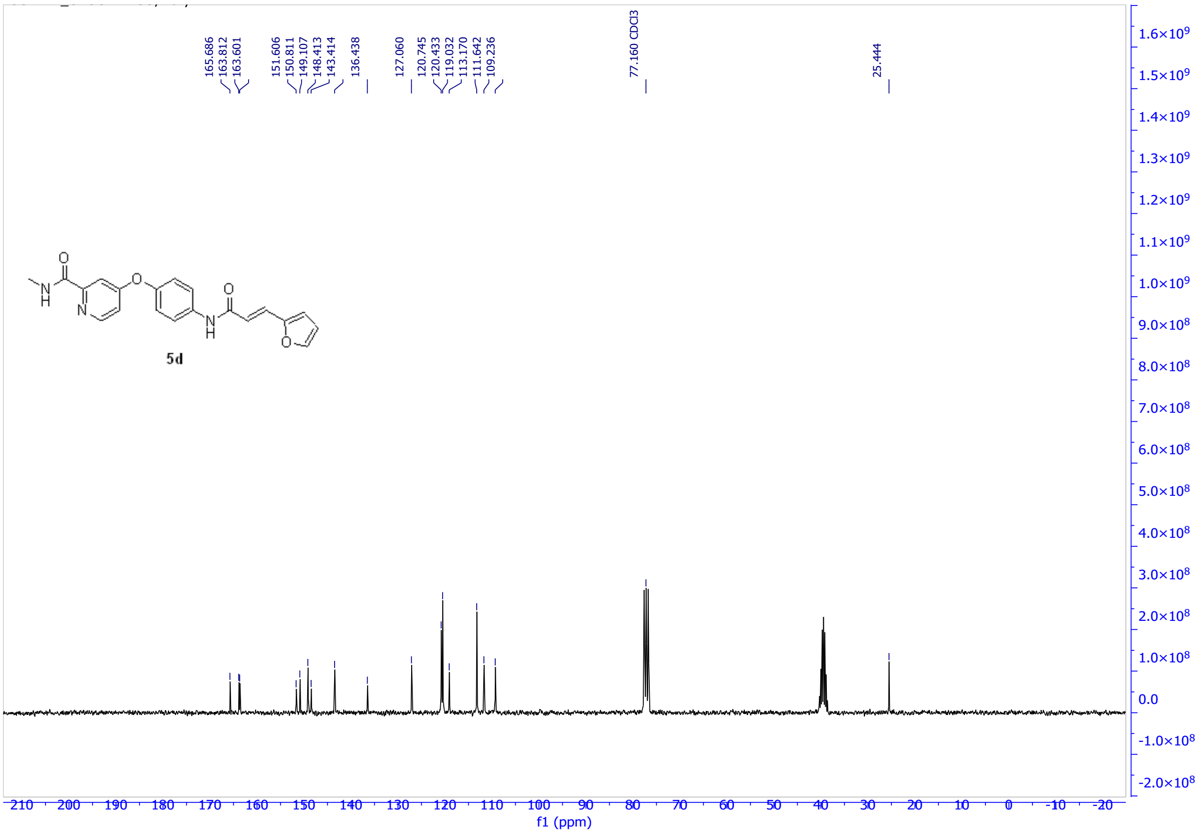
165.686
163.812
163.601
151.606
150.811
149.107
148.413
143.414
136.438
127.060
120.745
120.433
119.032
113.170
111.642
109.236
77.160 CDCl3
25.444
5d
f1 (ppm)

Compound **5e**

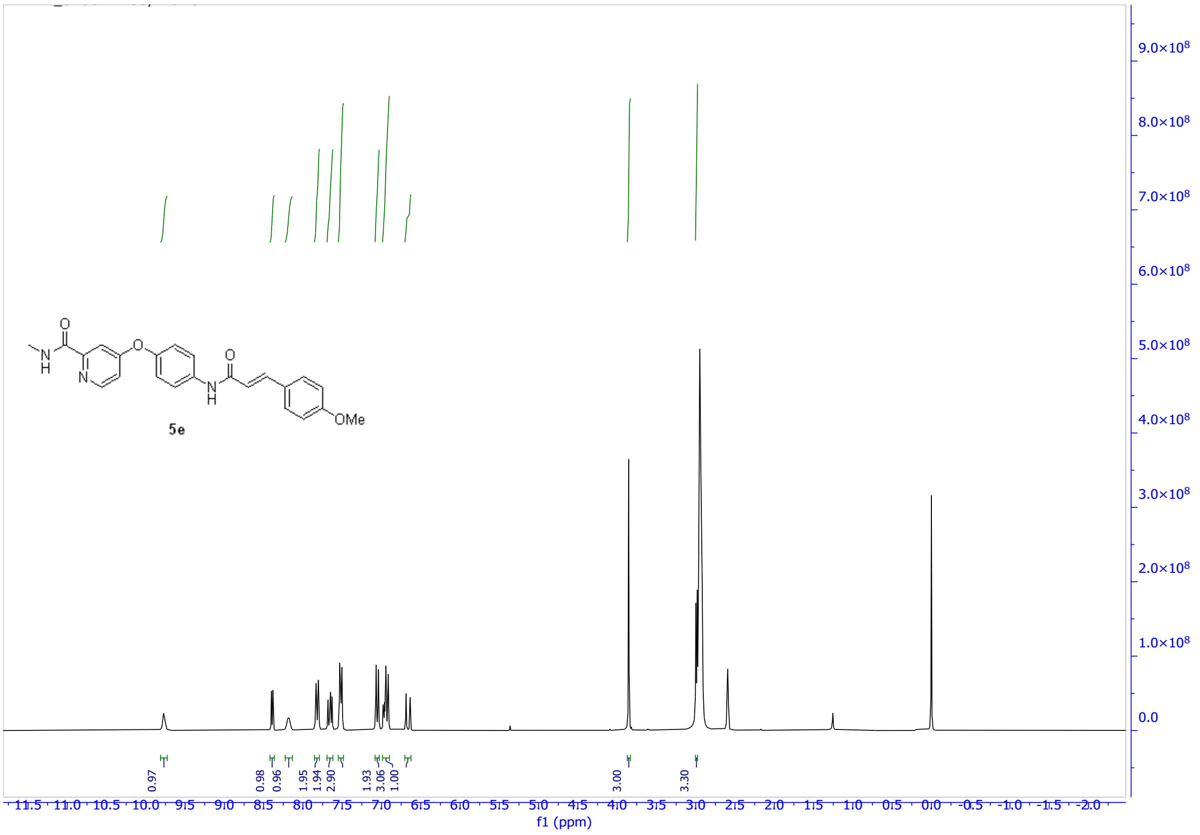
0.97
0.98
0.96
1.95
1.94
2.90
1.93
3.06
1.00
3.00
3.30
5e
OMe
f1 (ppm)

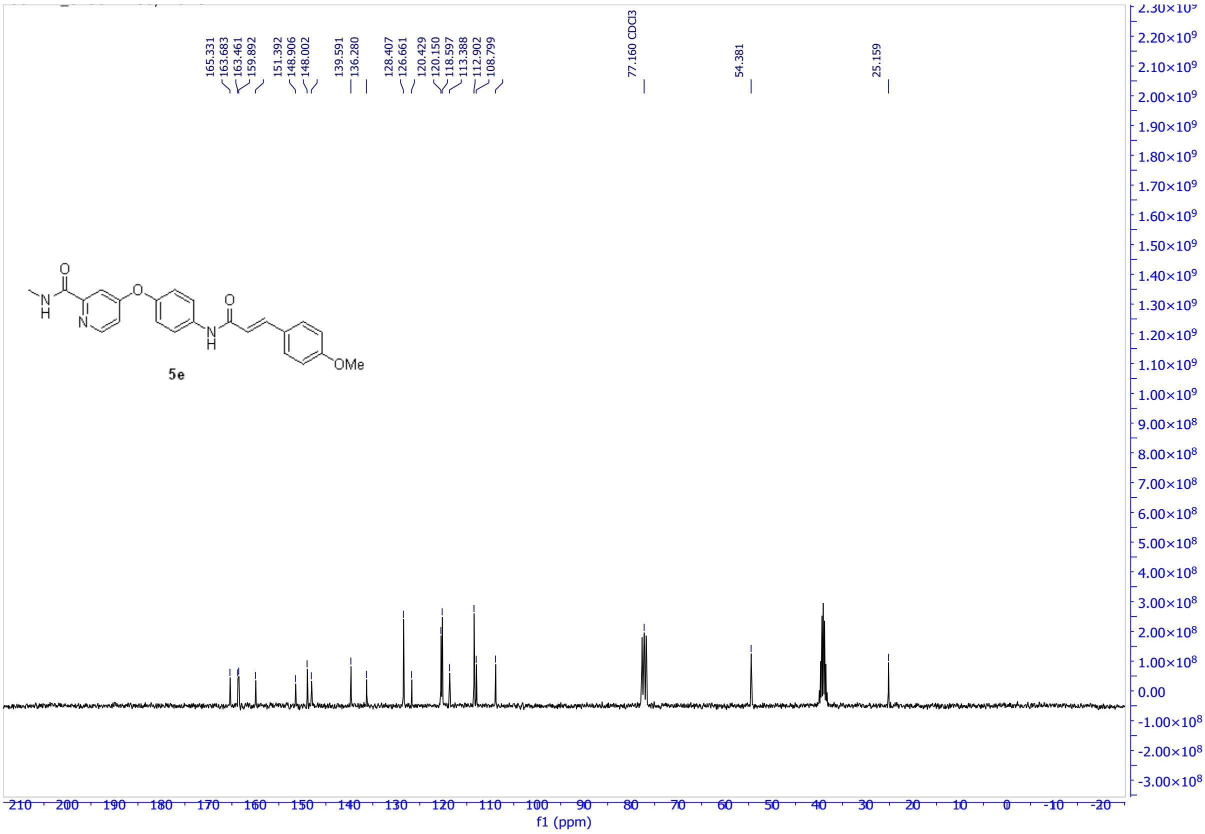
165.331
163.683
163.461
159.892
151.392
148.906
148.002
139.591
136.280
128.407
126.661
120.429
120.150
118.597
113.388
112.902
108.799
77.160 CDCl3
54.381
25.159
5e
OMe
f1 (ppm)

Compound **5f**

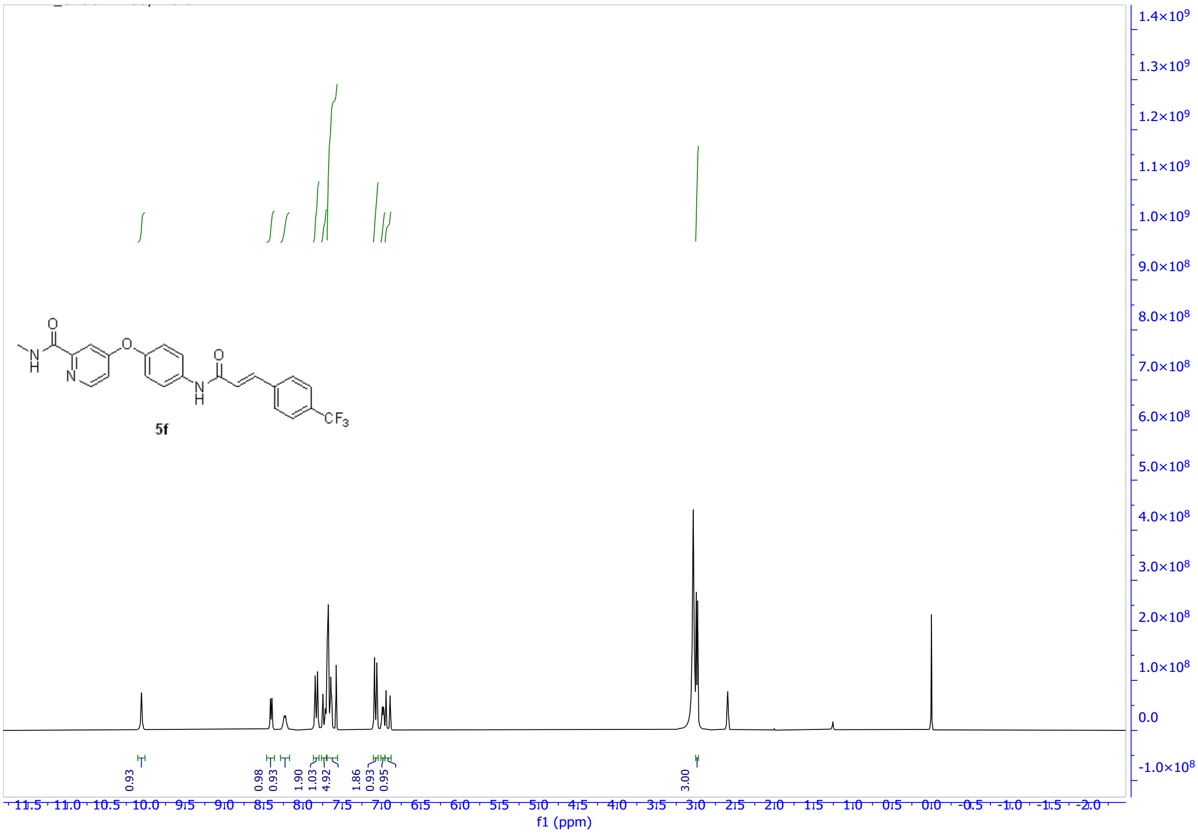
5f
CF_3
0.93
0.98
0.93
1.90
1.03
4.92
1.86
0.93
0.95
3.00
f1 (ppm)

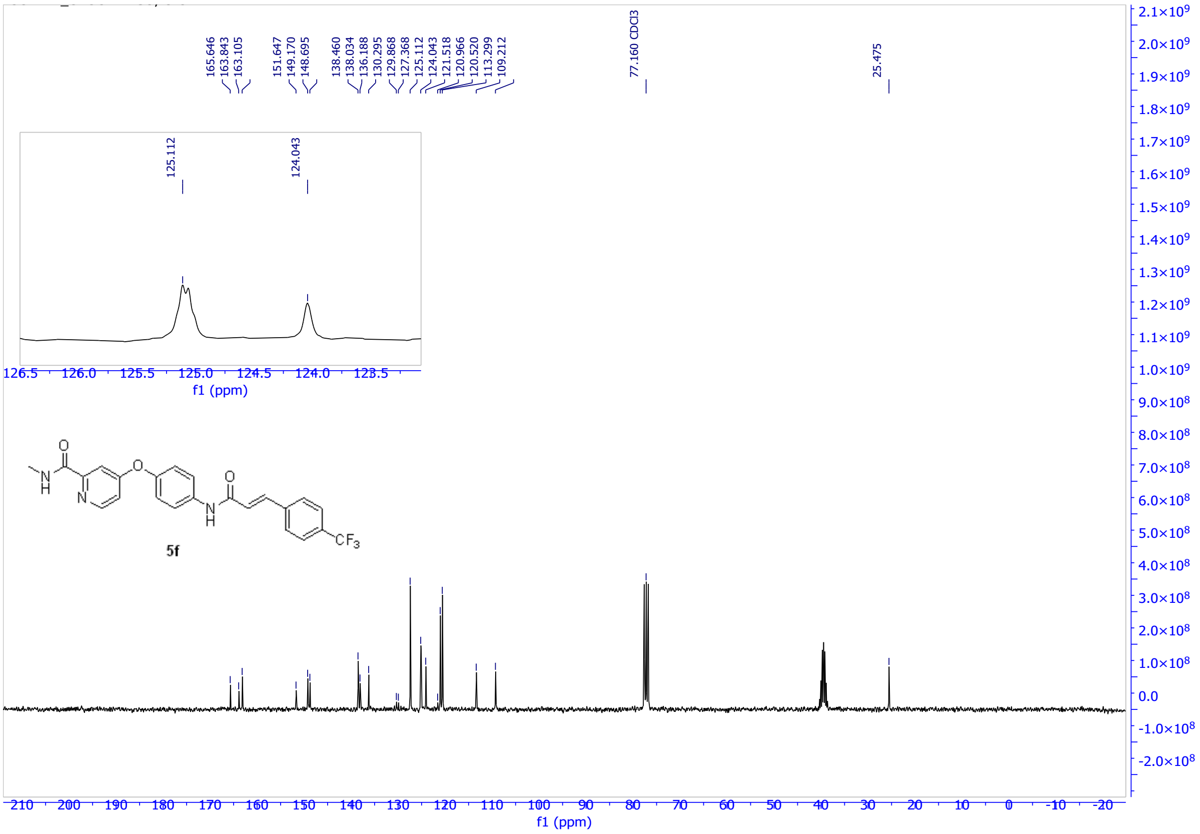

Compound **5g**

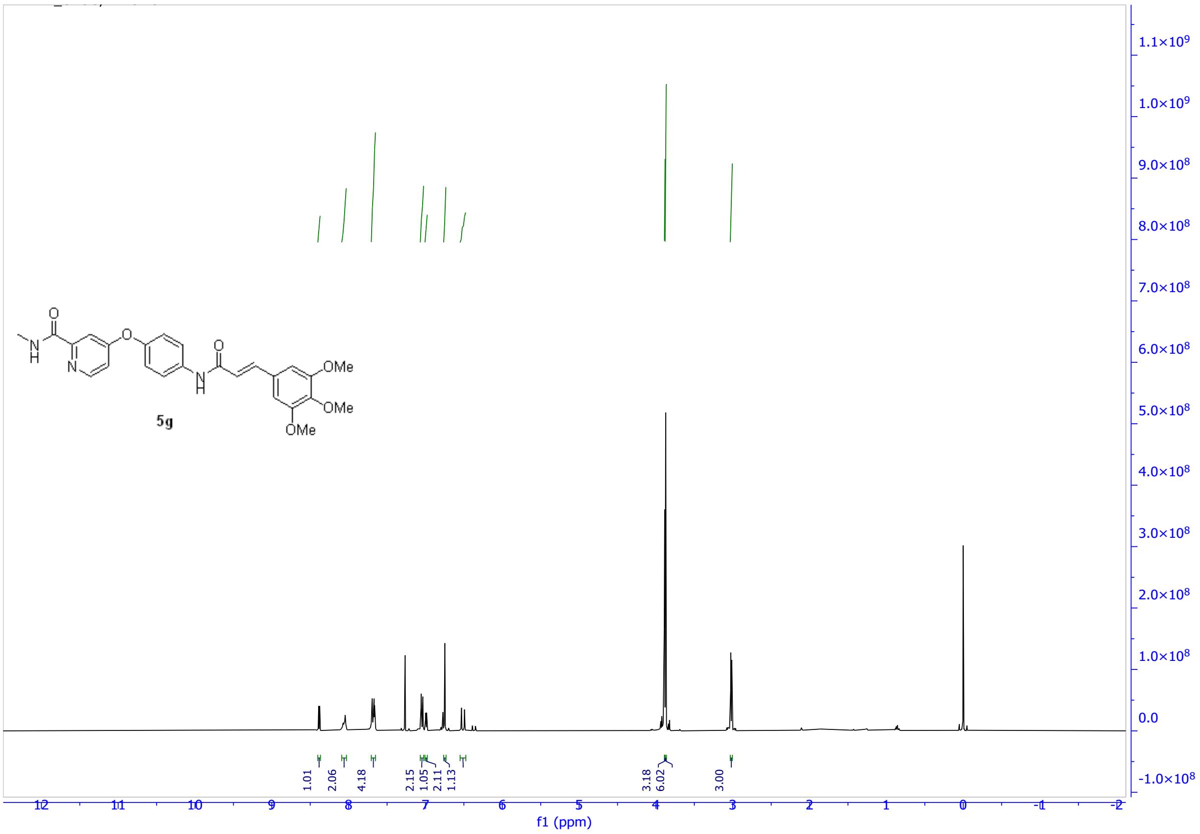

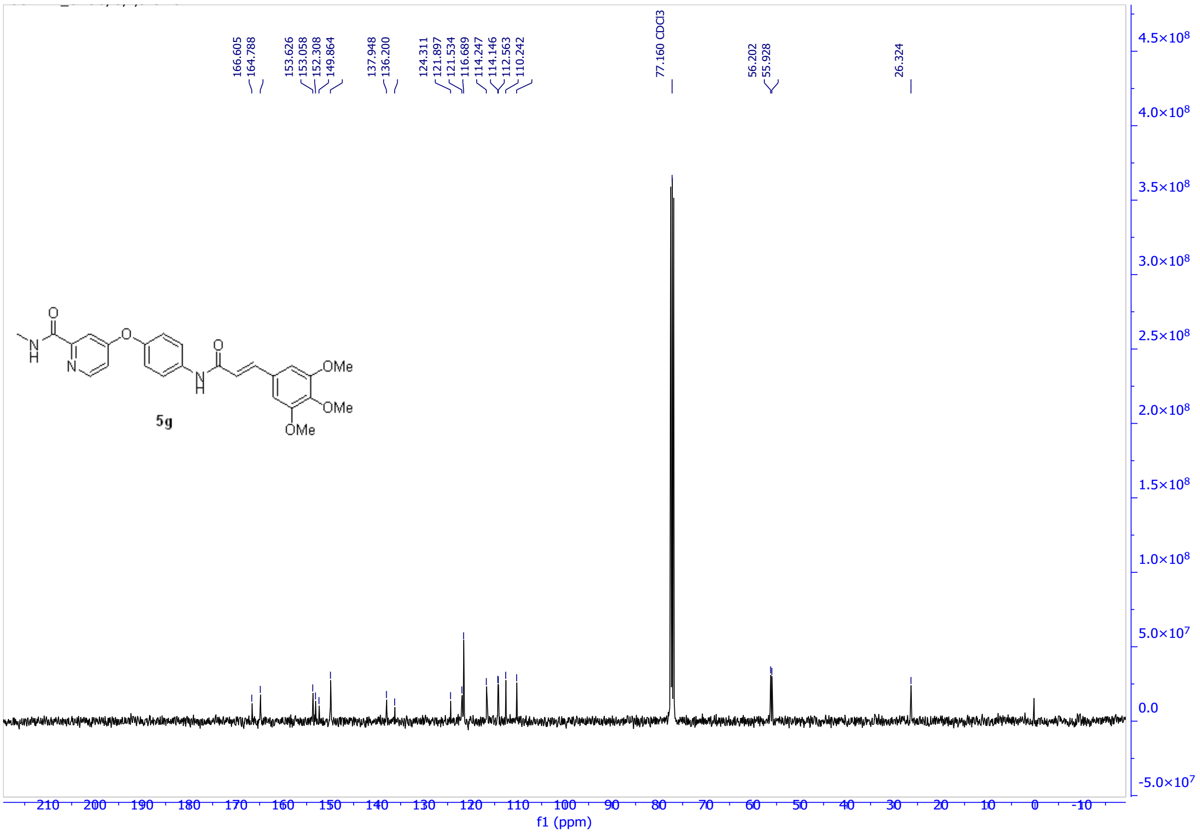

Compound **5h**

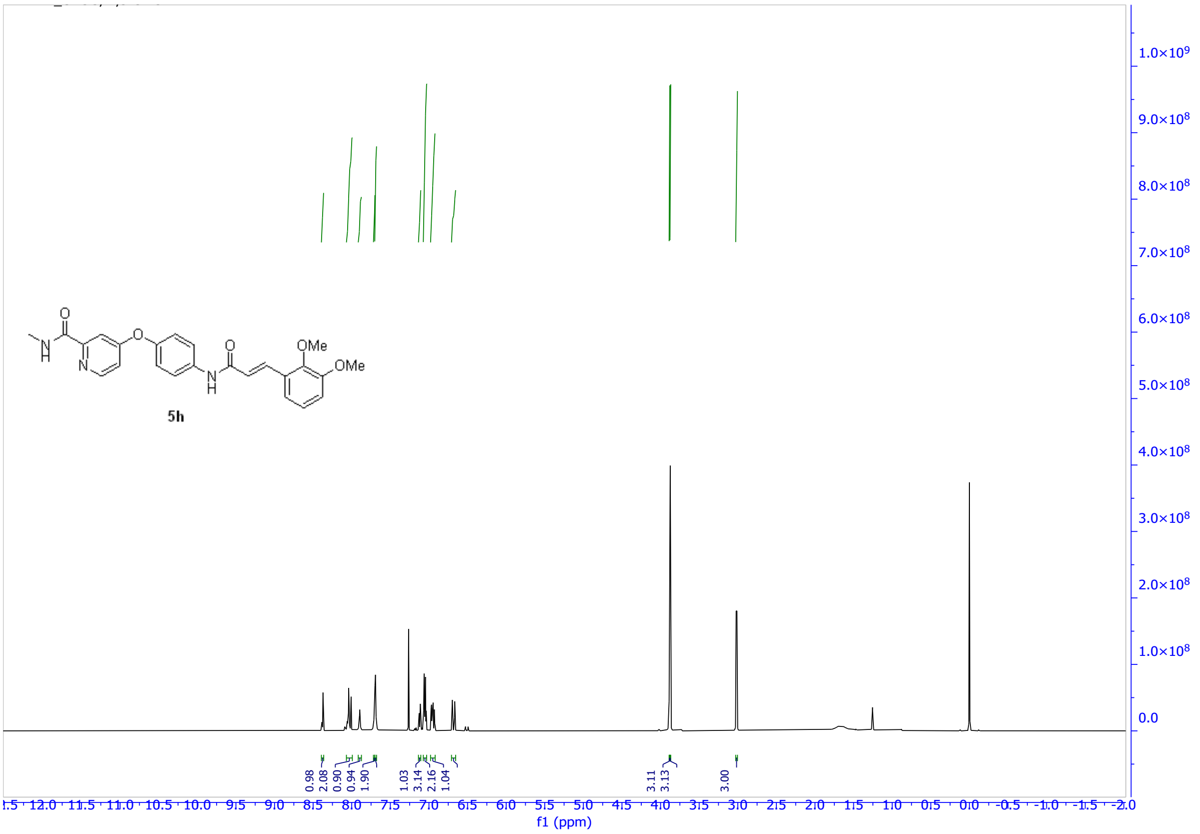

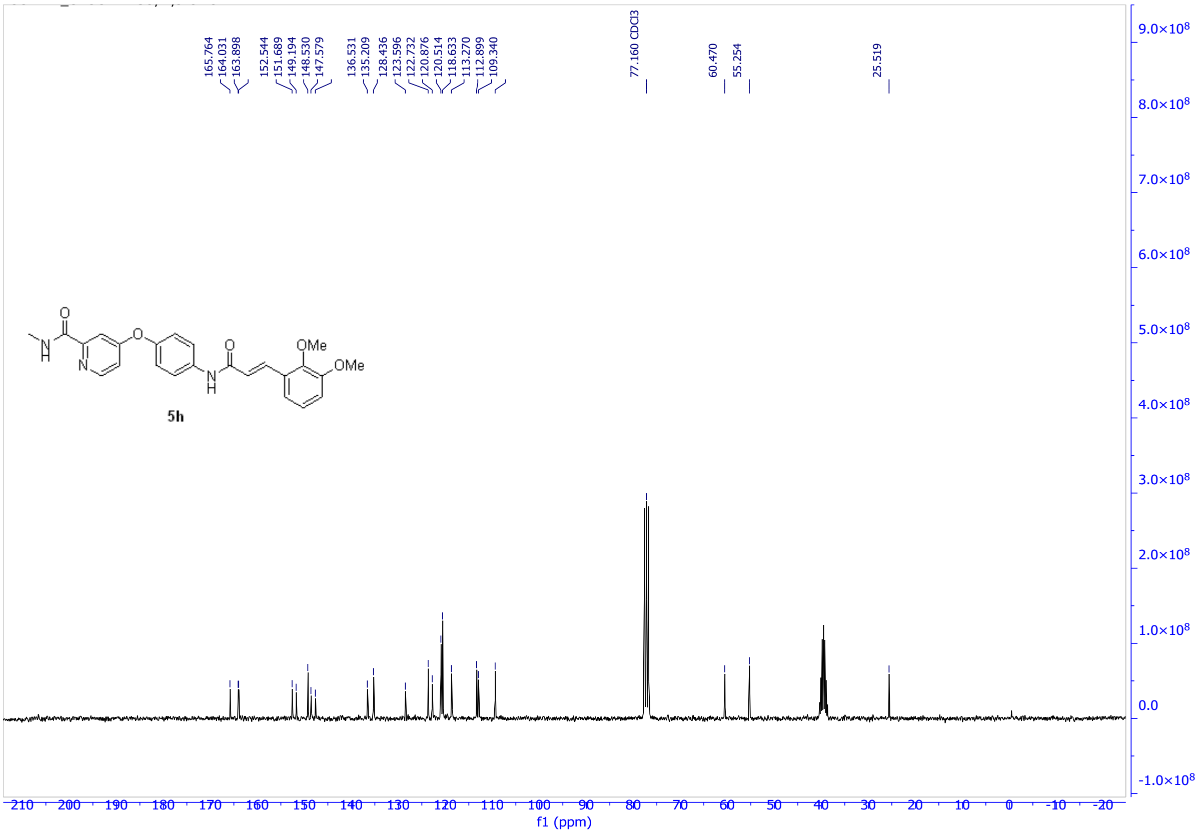

165.764
164.031
163.898
152.544
151.689
149.194
148.530
147.579
136.531
135.209
128.436
123.596
122.732
120.876
120.514
118.633
113.270
112.899
109.340
77.160 CDCl3
60.470
55.254
25.519
OMe
OMe
5h
f1 (ppm)

Compound **5i**

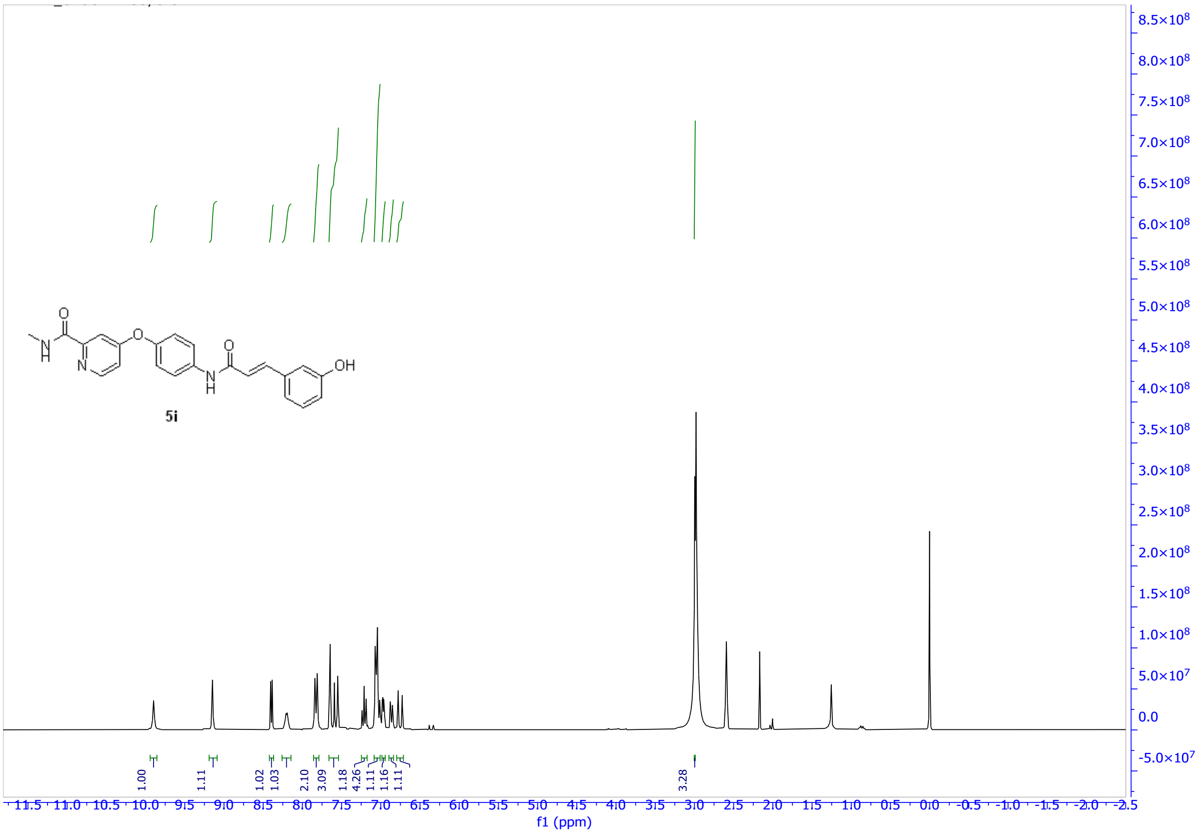

OH
5i
1.00
1.11
1.02
1.03
2.10
3.09
1.18
4.26
1.11
1.16
1.11
3.28
f1 (ppm)

Compound **5j**

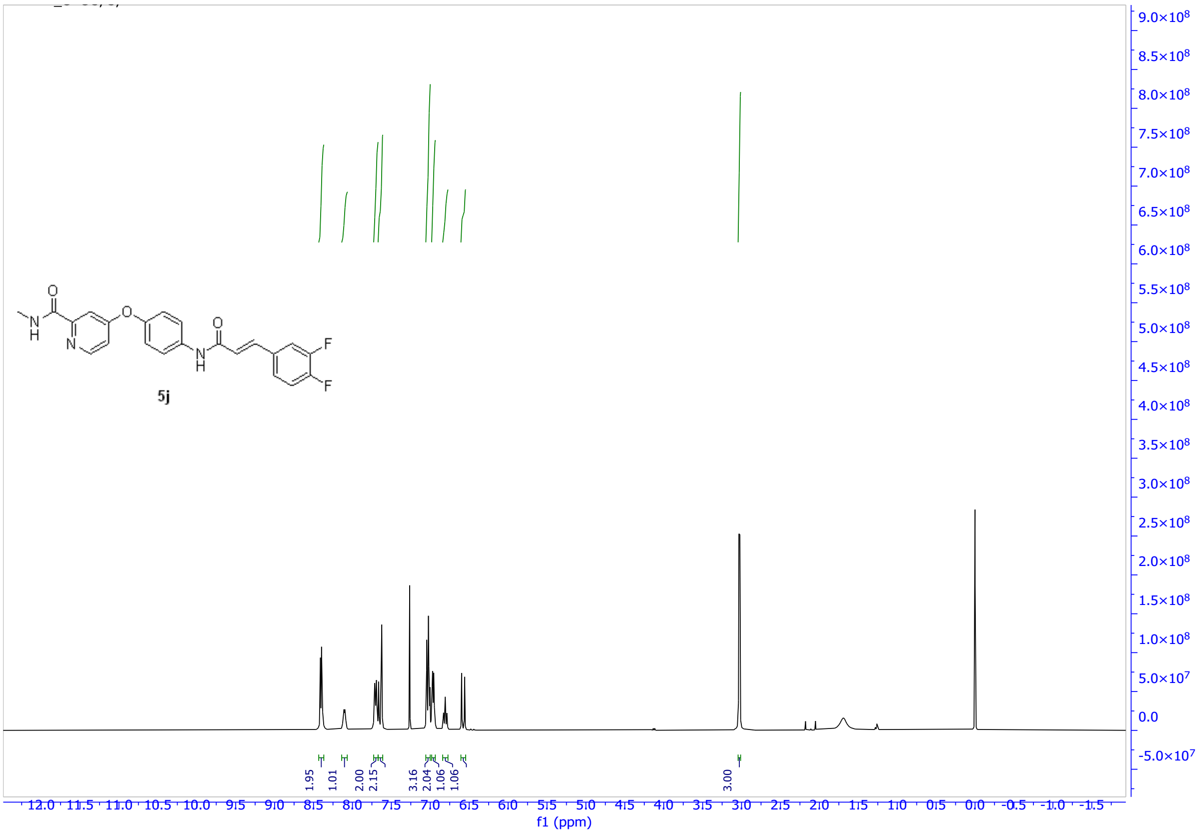

5j
1.95
1.01
2.00
2.15
3.16
2.04
1.06
1.06
3.00
f1 (ppm)

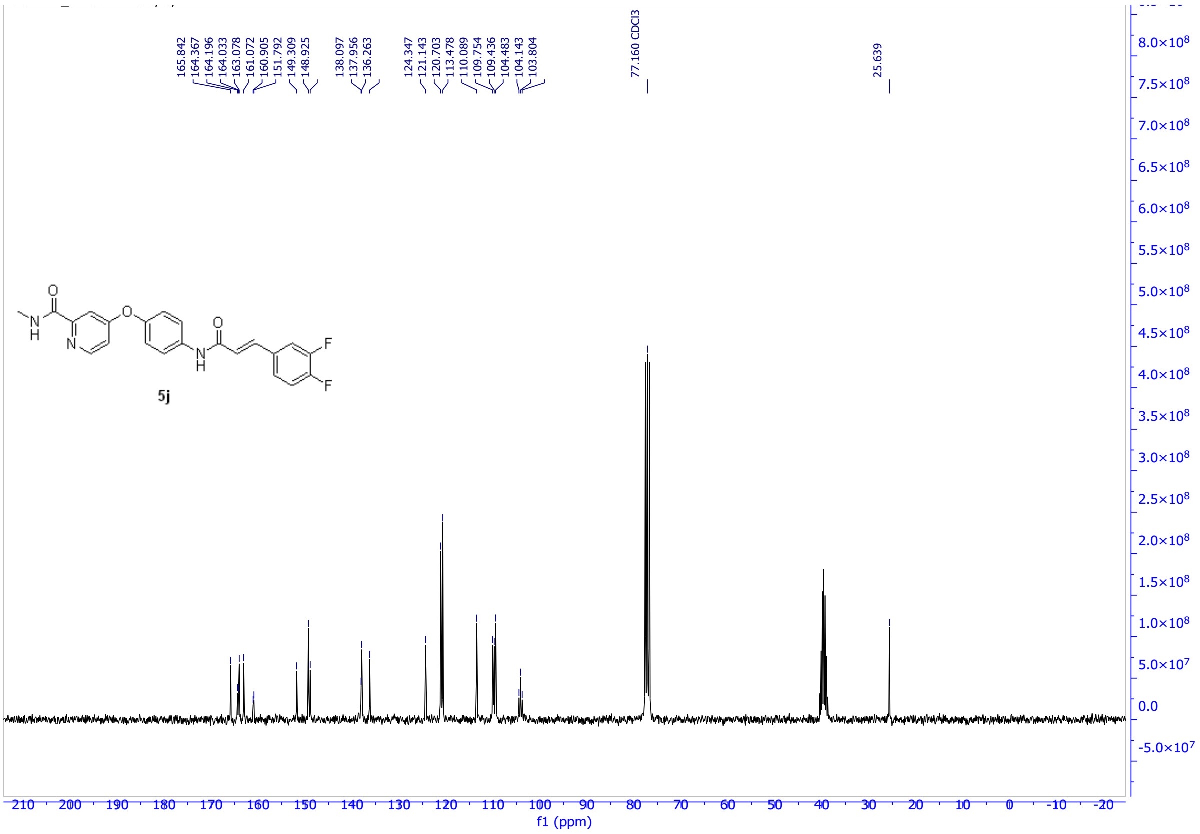

165.842
164.367
164.196
164.033
163.078
161.072
160.905
151.792
149.309
148.925
138.097
137.956
136.263
124.347
121.143
120.703
113.478
110.089
109.754
109.436
104.483
104.143
103.804
77.160 CDCl3
25.639
5j
f1 (ppm)

Compound **5k**

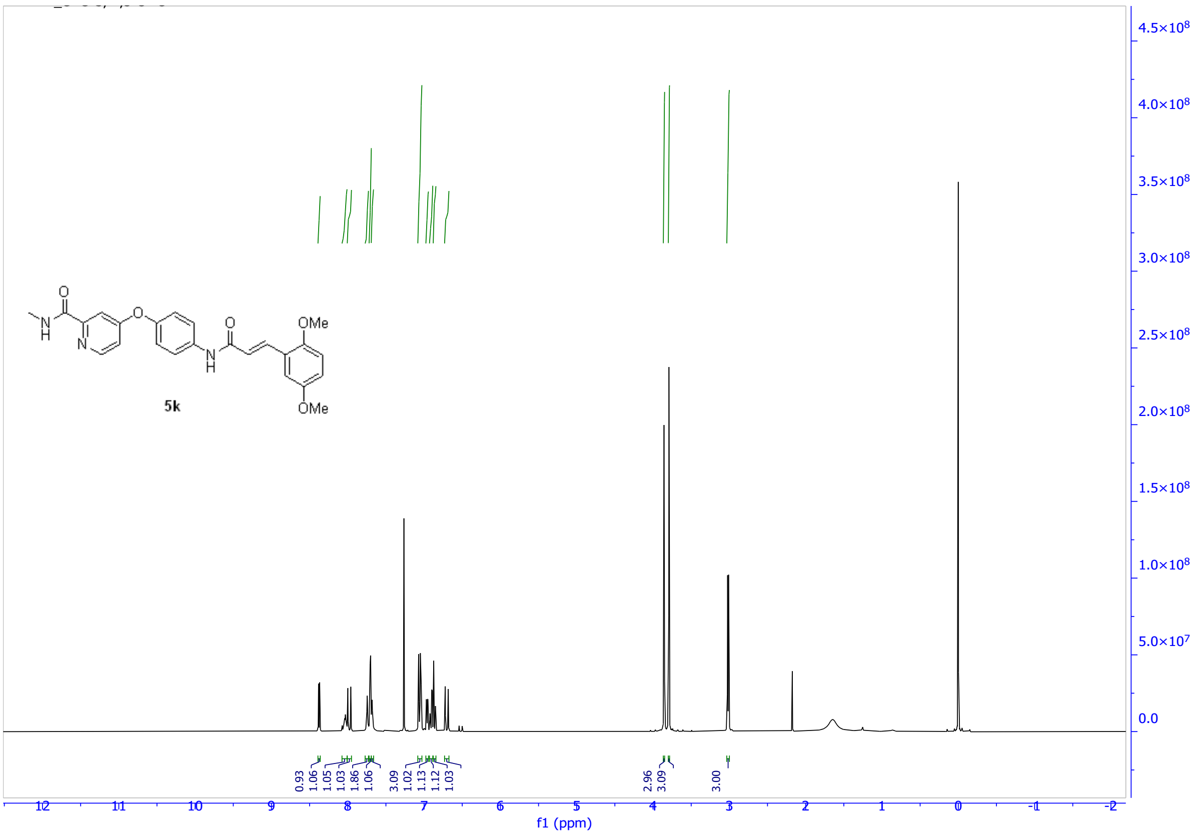

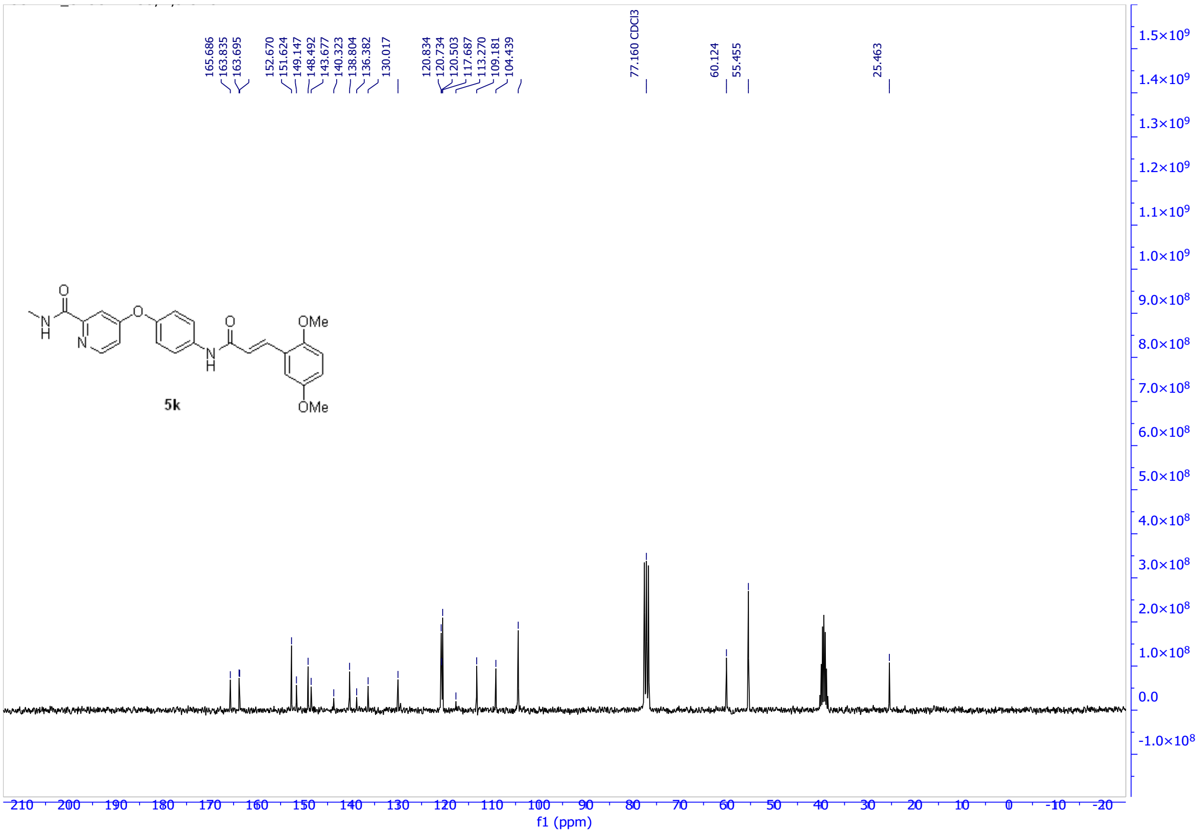

Compound **5m**

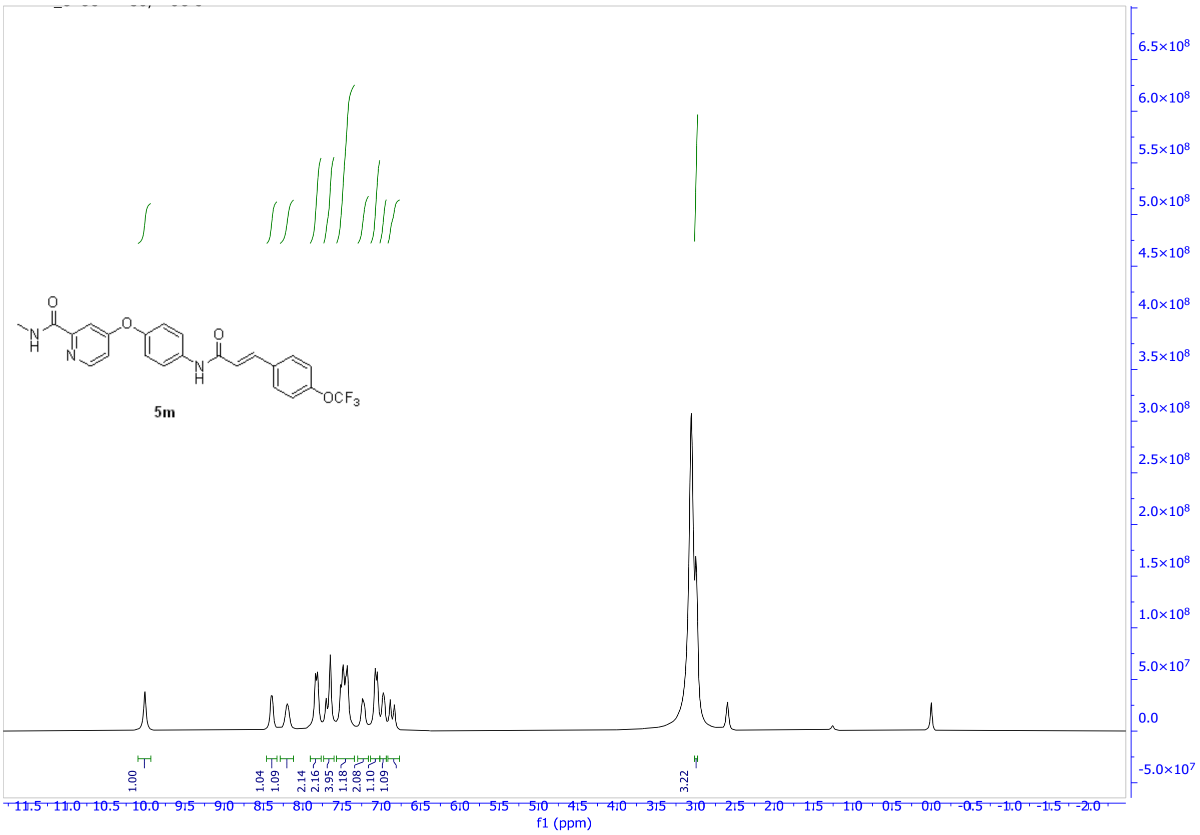

5m
1.00
1.04
1.09
2.14
2.16
3.95
1.18
2.08
1.10
1.09
3.22
f1 (ppm)

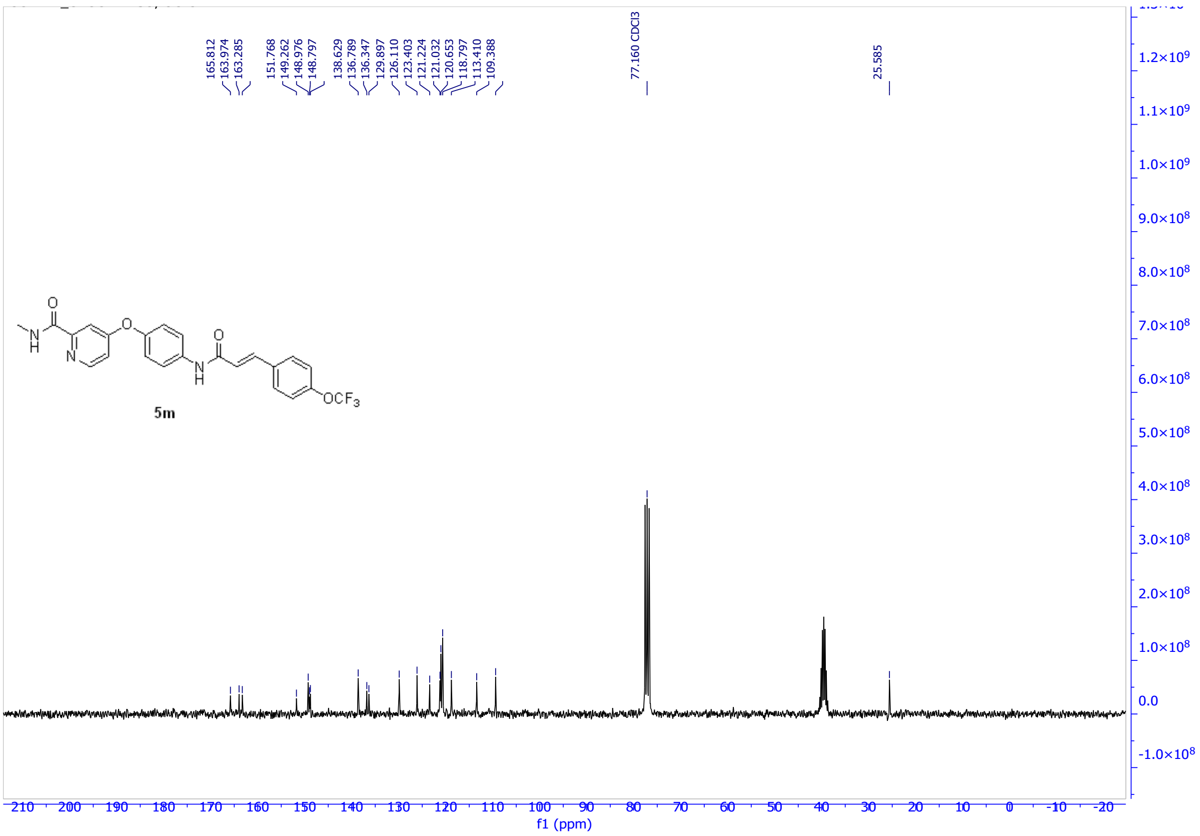

165.812
163.974
163.285
151.768
149.262
148.976
148.797
138.629
136.789
136.347
129.897
126.110
123.403
121.224
121.032
120.653
118.797
113.410
109.388
77.160 CDCl3
25.585
5m
f1 (ppm)

Compound **5n**

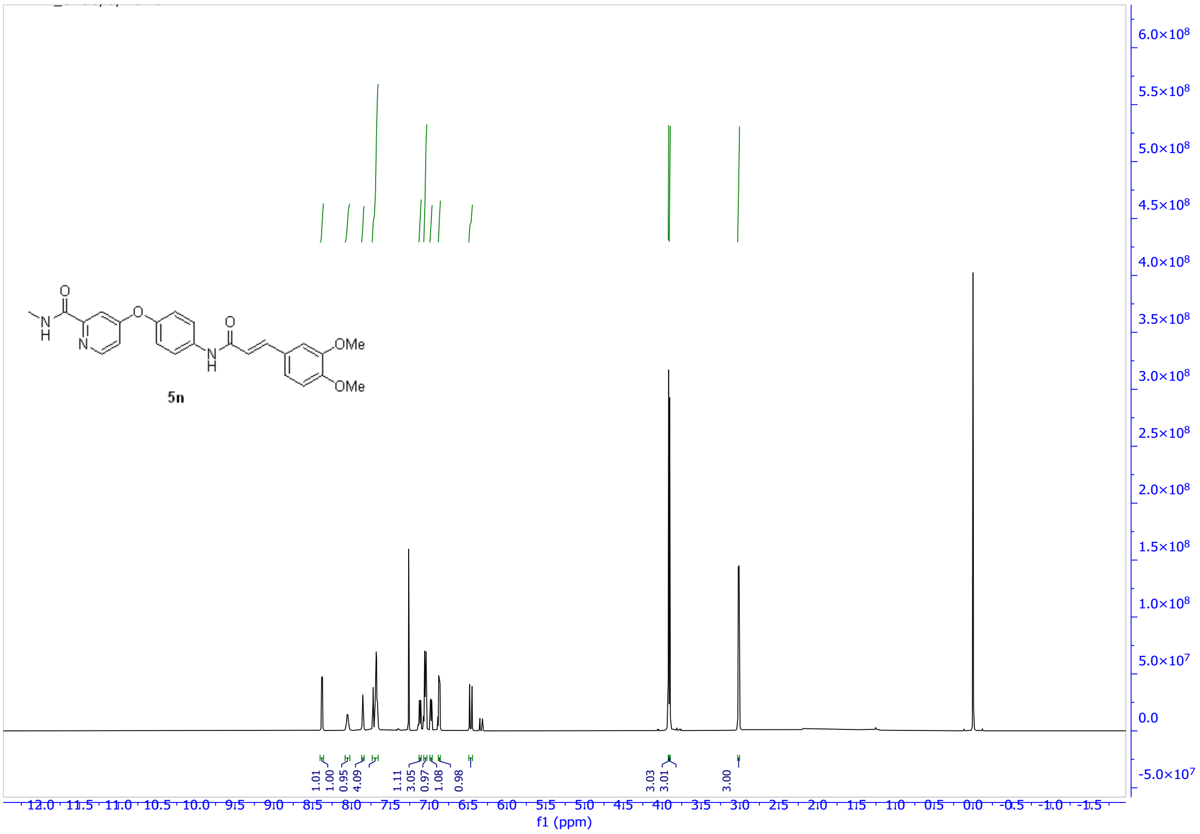

Compound **5o**

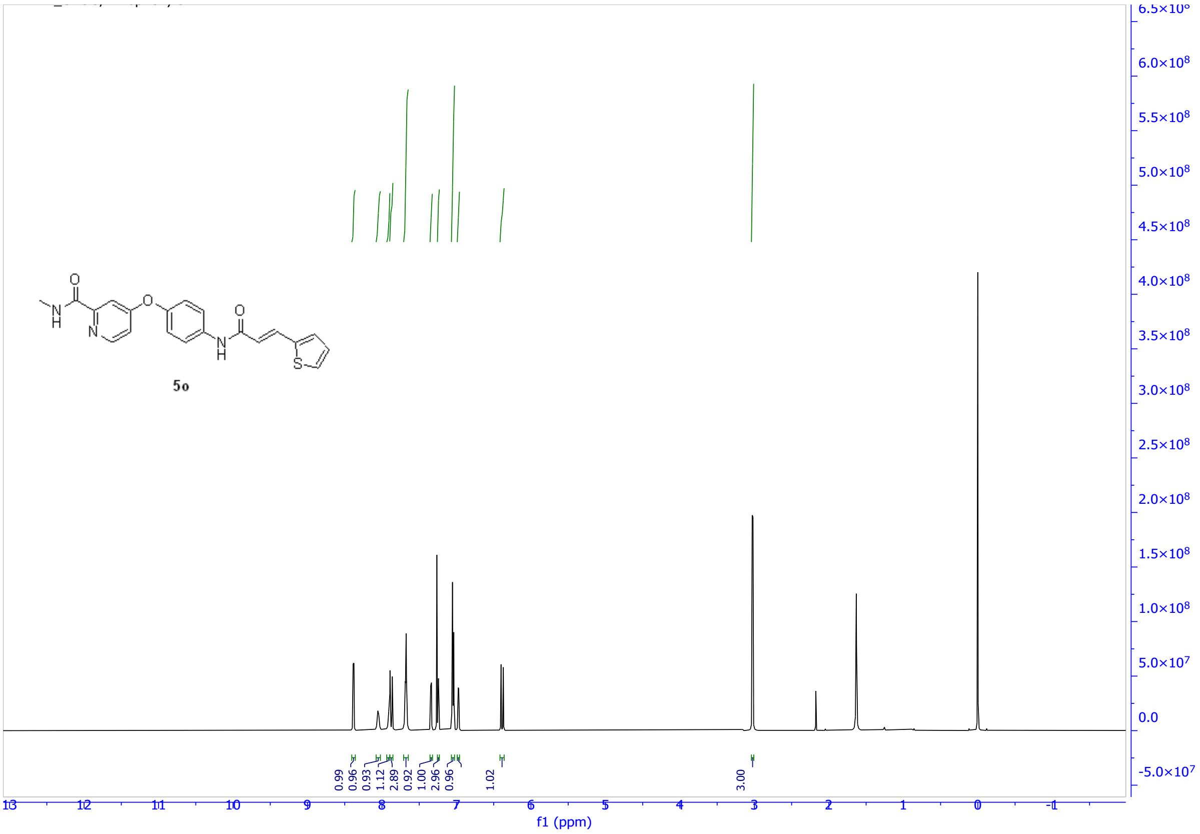

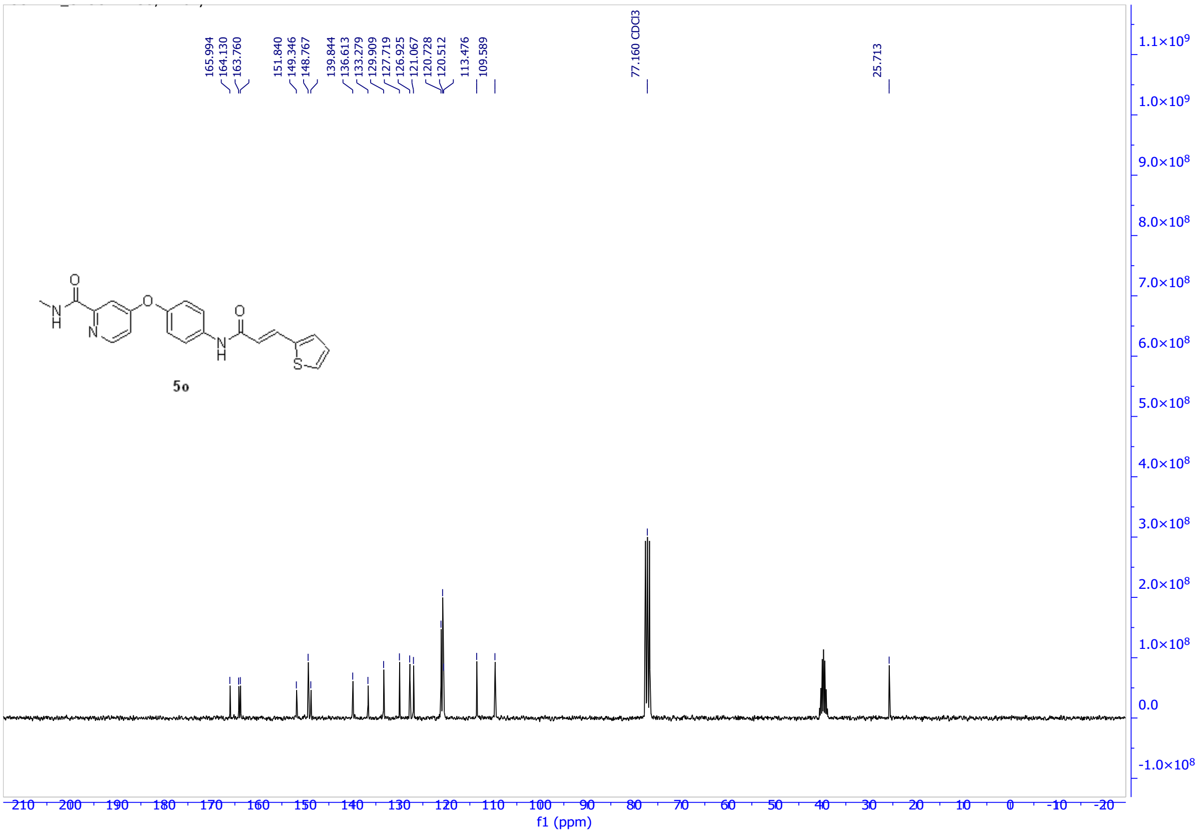
165.994
164.130
163.760
151.840
149.346
148.767
139.844
136.613
133.279
129.909
127.719
126.925
121.067
120.728
120.512
113.476
109.589
77.160 CDCl3
25.713
5o
f1 (ppm)

Compound **5p**

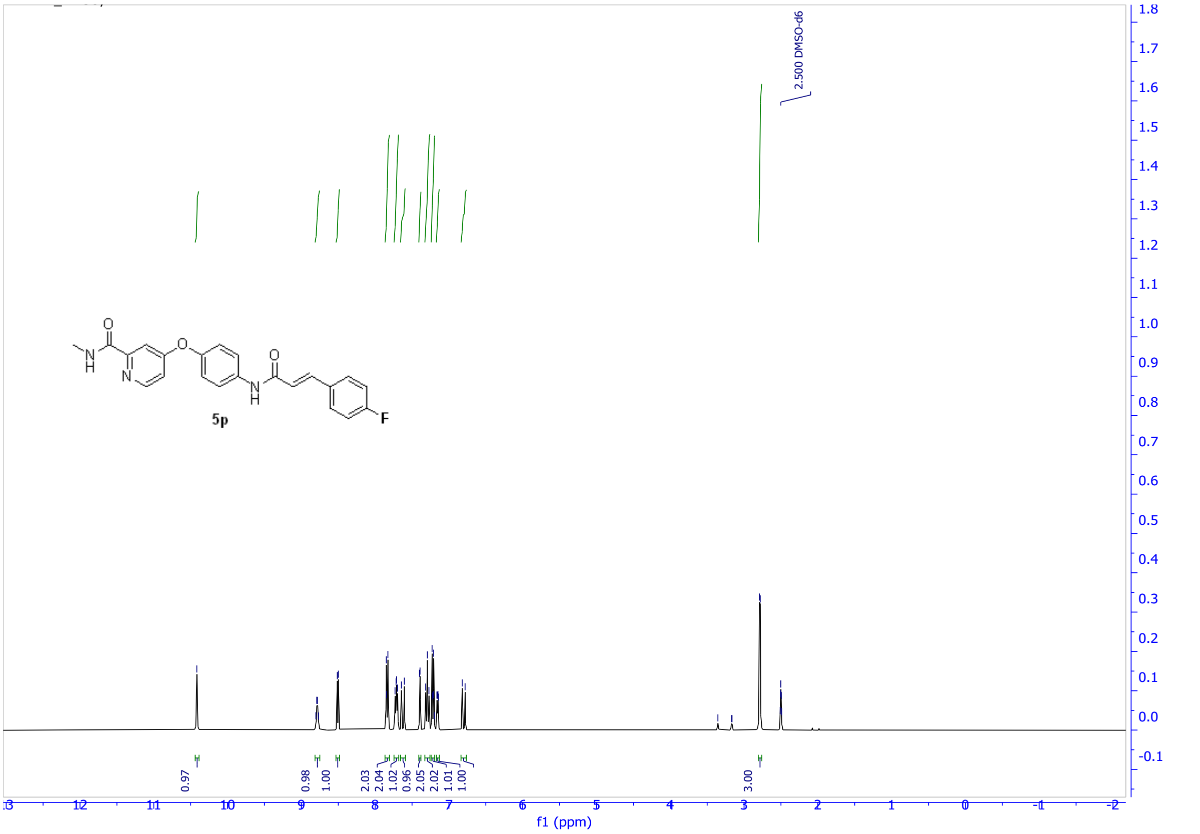
2.500 DMSO-d6
5p
0.97
0.98
1.00
2.03
2.04
1.02
0.96
2.05
2.02
1.01
1.00
3.00
f1 (ppm)

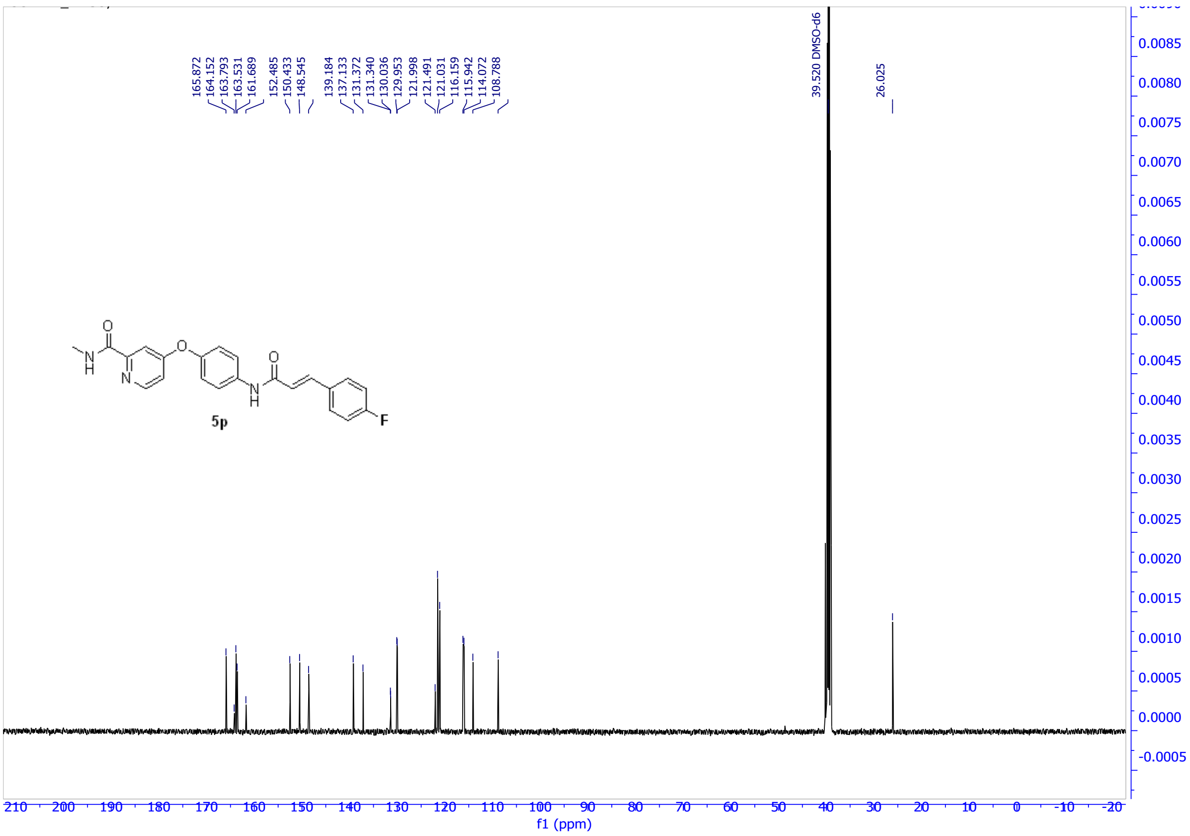
165.872
164.152
163.793
163.531
161.689
152.485
150.433
148.545
139.184
137.133
131.372
131.340
130.036
129.953
121.998
121.491
121.031
116.159
115.942
114.072
108.788
39.520 DMSO-d6
26.025
5p
f1 (ppm)

Compound **5q**

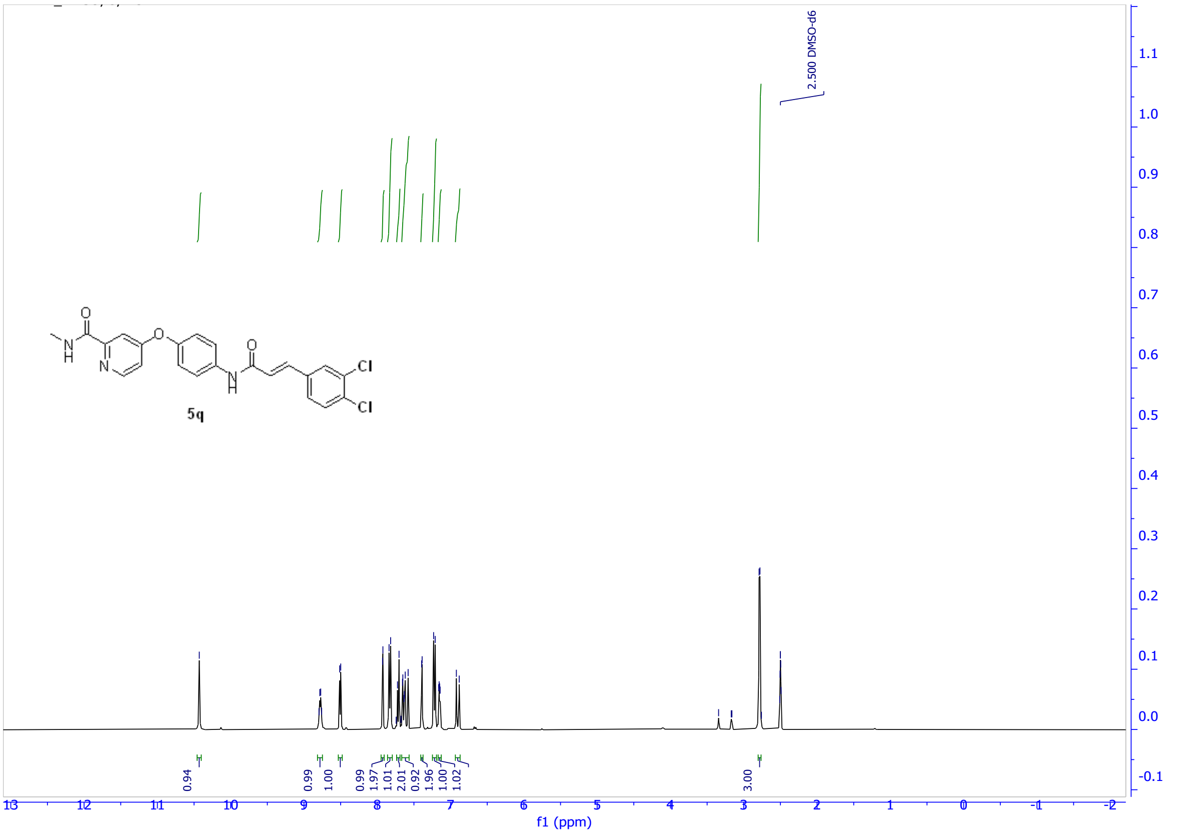
2.500 DMSO-d6
5q
0.94
0.99
1.00
0.99
1.97
1.01
2.01
0.92
1.96
1.00
1.02
3.00
f1 (ppm)

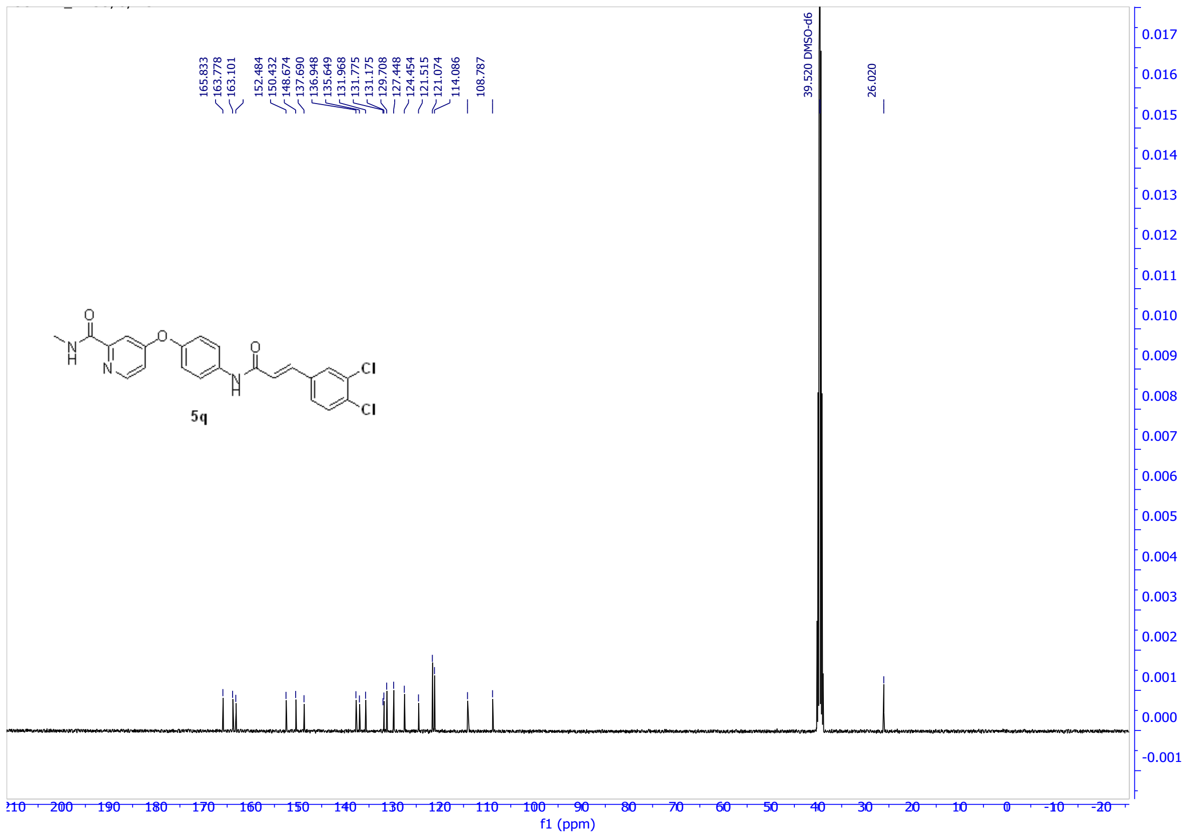
165.833
163.778
163.101
152.484
150.432
148.674
137.690
136.948
135.649
131.968
131.775
131.175
129.708
127.448
124.454
121.515
121.074
114.086
108.787
39.520 DMSO-d6
26.020
5q
f1 (ppm)

Compound **5r**

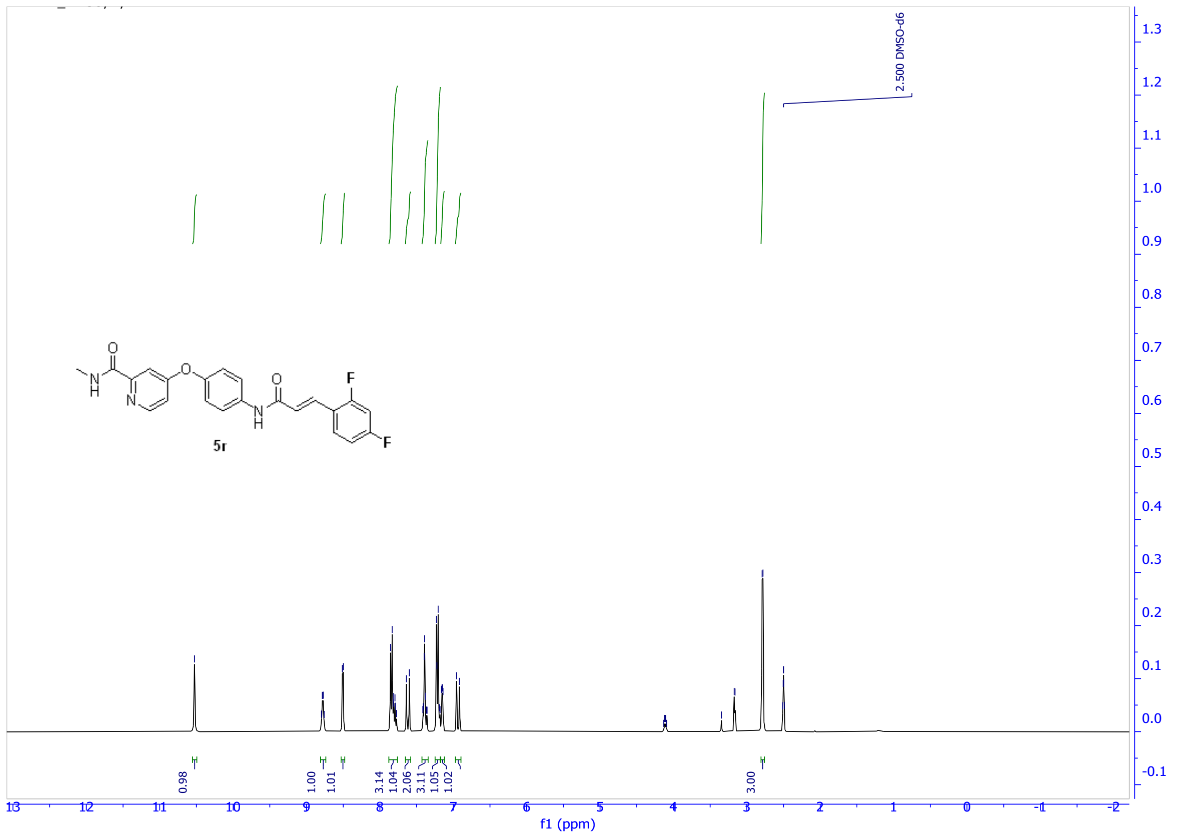
2.500 DMSO-d6
5r
0.98
1.00
1.01
3.14
1.04
2.06
3.11
1.05
1.02
3.00
f1 (ppm)

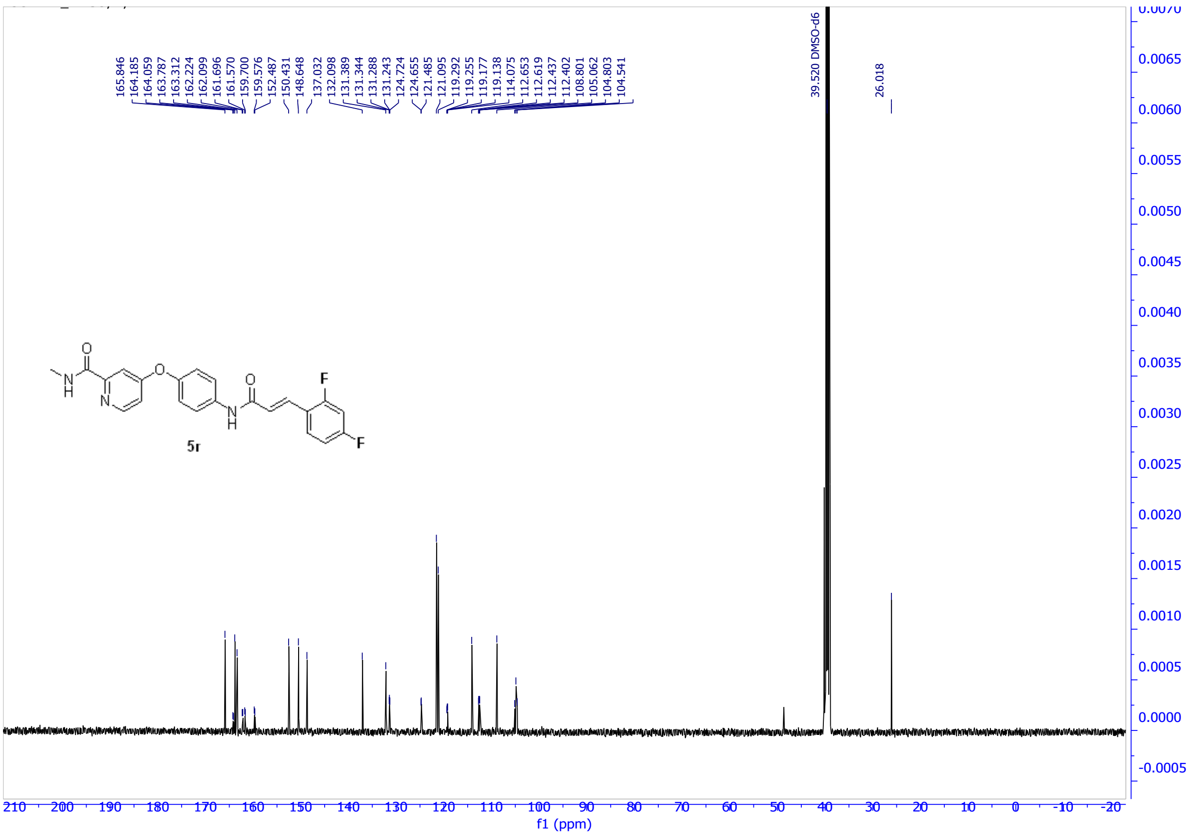
165.846
164.185
164.059
163.787
163.312
162.224
162.099
161.696
161.570
159.700
159.576
152.487
150.431
148.648
137.032
132.098
131.389
131.344
131.288
131.243
124.724
124.655
121.485
121.095
119.292
119.255
119.177
119.138
114.075
112.653
112.619
112.437
112.402
108.801
105.062
104.803
104.541
39.520 DMSO-d6
26.018
5r
f1 (ppm)

Compound **5s**

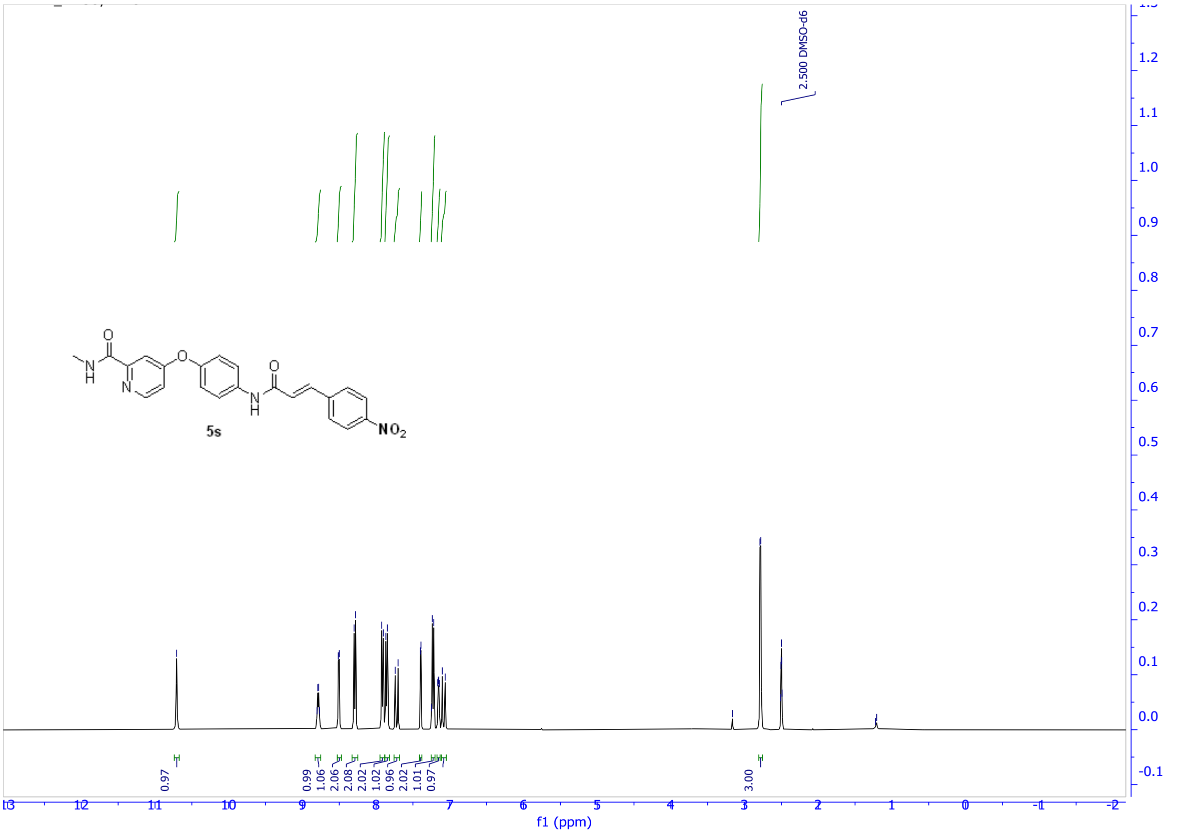
2.500 DMSO-d6
5s
0.97
0.99
1.06
2.06
2.08
2.02
1.02
0.96
2.02
1.01
0.97
3.00
f1 (ppm)

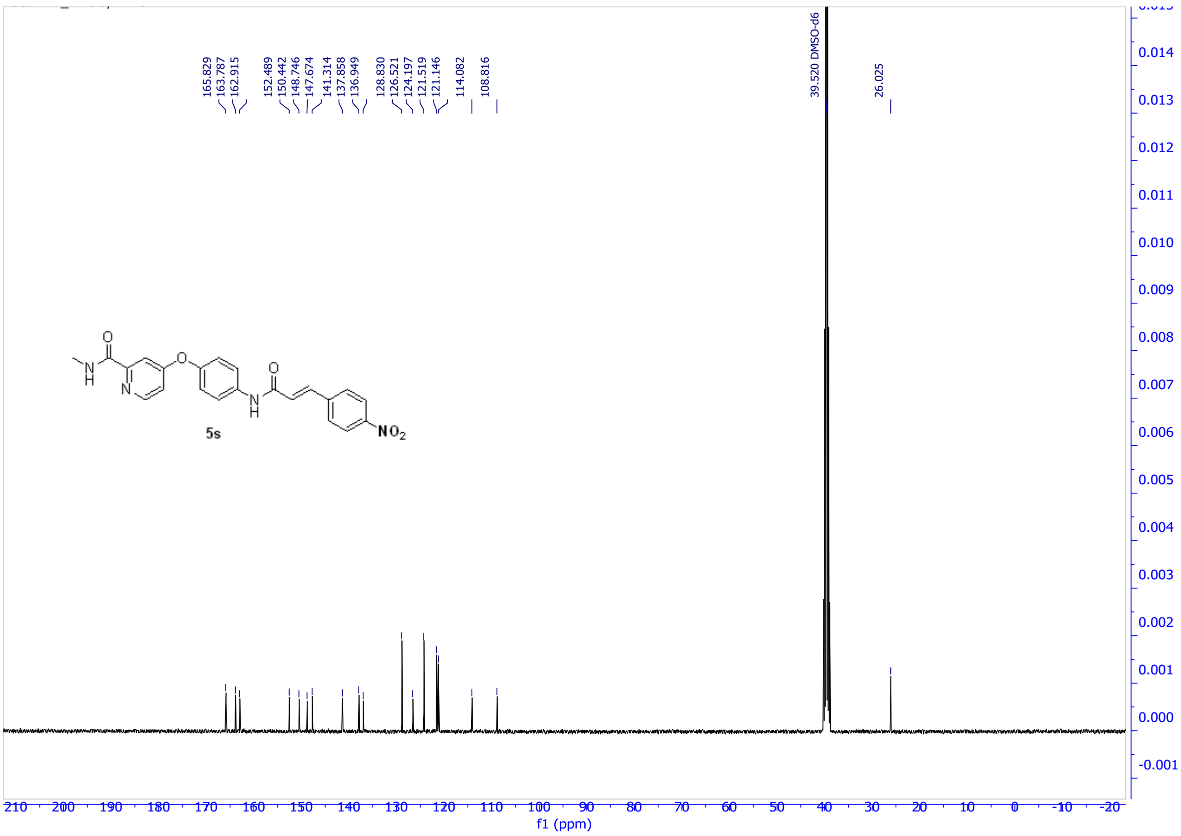
165.829
163.787
162.915
152.489
150.442
148.746
147.674
141.314
137.858
136.949
128.830
126.521
124.197
121.519
121.146
114.082
108.816
39.520 DMSO-d6
26.025
5s
NO2
f1 (ppm)

Mass and HRMS Spectra

Compound **5a**_Mass

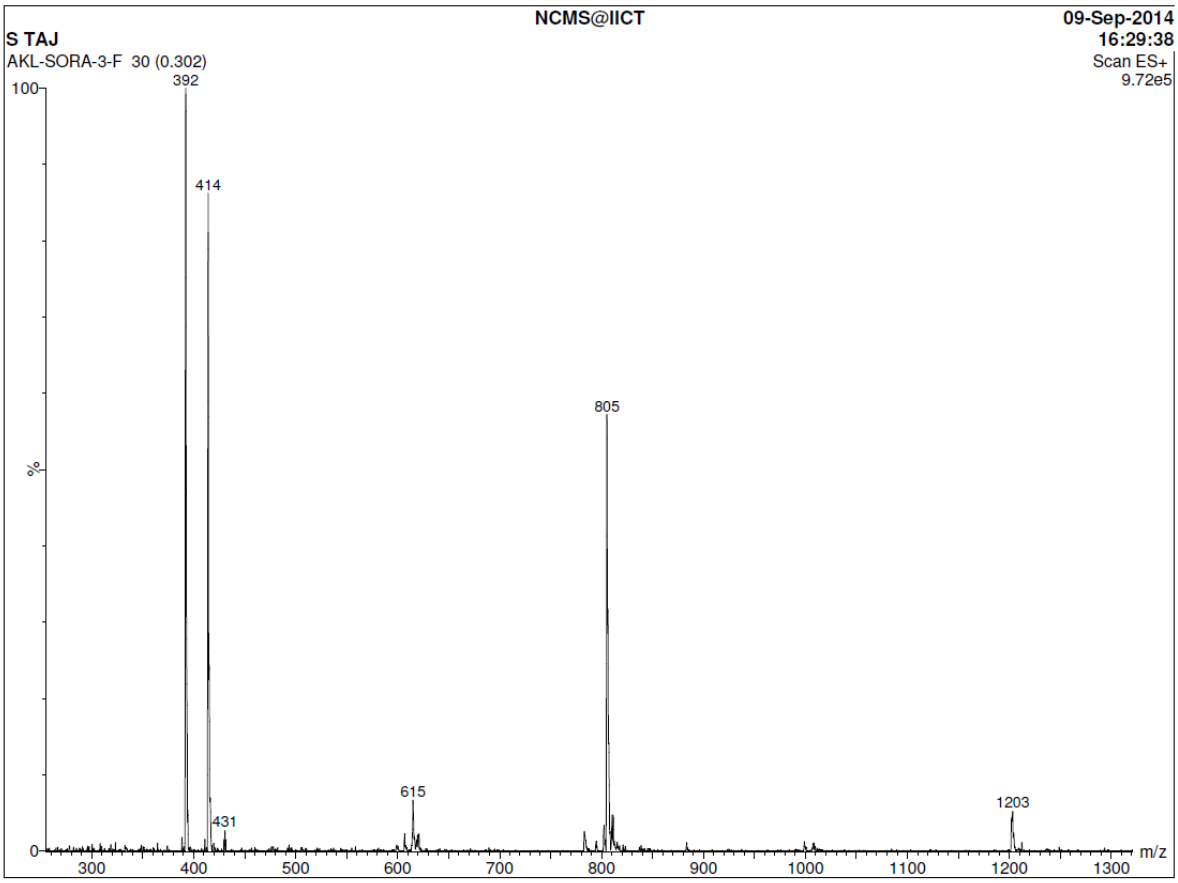

Compound **5a**_HRMS

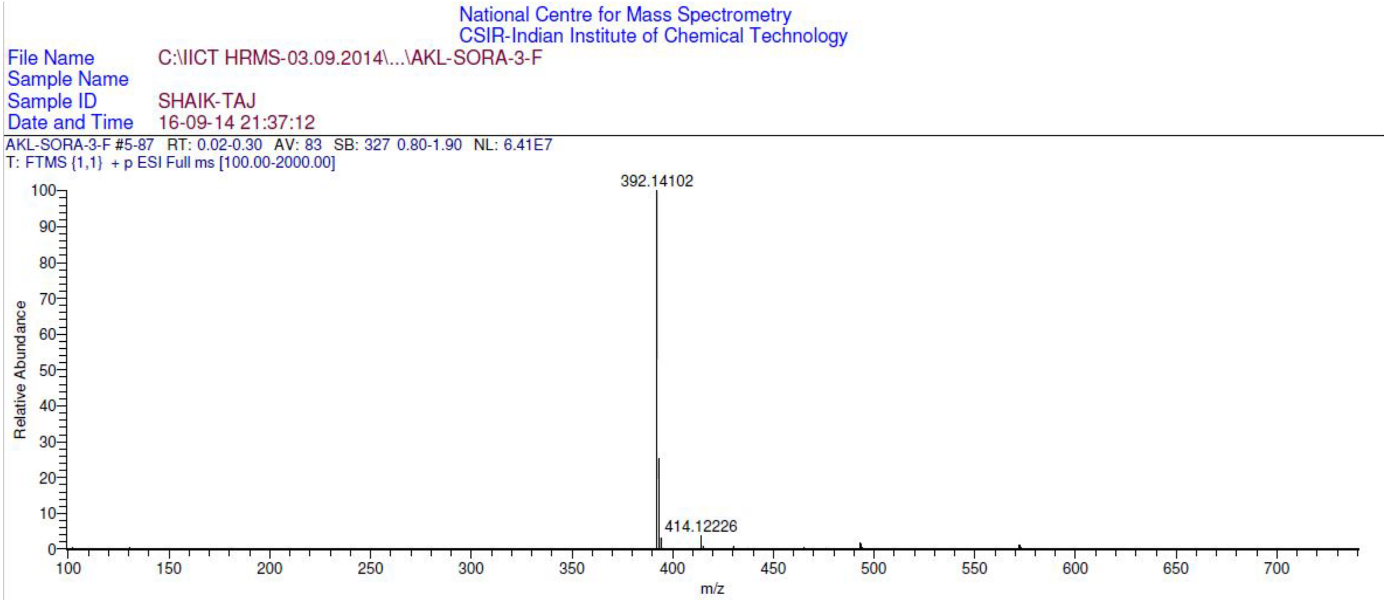

AKL-SORA-3-F#8-30 RT: 0.03-0.11 AV: 23
T: FTMS {1,1} + p ESI Full ms [100.00-2000.00]
m/z= 366.38-429.80

m/z	Intensity	Relative	Theo. Mass	Delta (ppm)	RDB equiv.	Composition
392.14089	55654464.0	100.00	392.14050	0.99	14.5	$C_{22}H_{19}O_3N_3F$

Compound **5b**

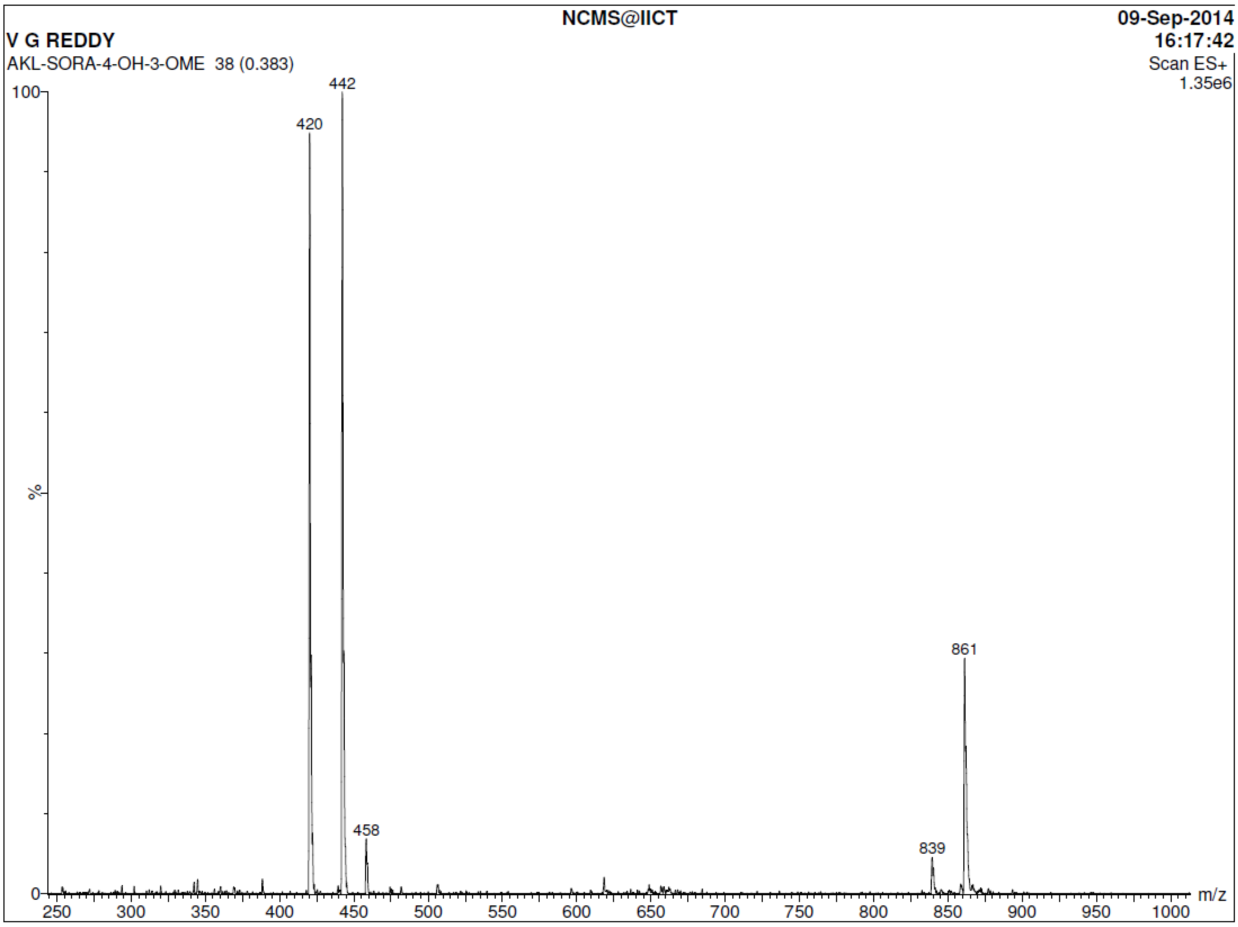

Compound **5b**

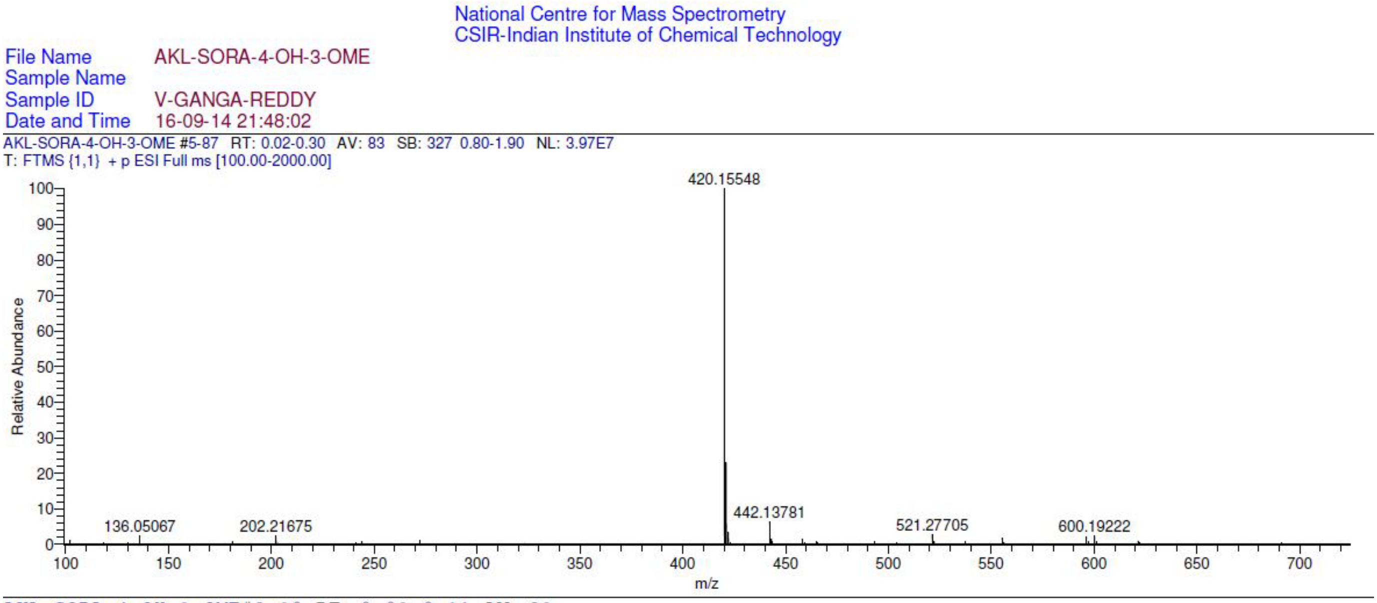

AKL-SORA-4-OH-3-OME#8-30 RT: 0.03-0.11 AV: 23
T: FTMS {1,1} + p ESI Full ms [100.00-2000.00]
m/z= 410.36-434.65

m/z	Intensity	Relative	Theo. Mass	Delta (ppm)	RDB equiv.	Composition
420.15547	44700872.0	100.00	420.15540	0.17	14.5	$C_{23}H_{22}O_5N_3$

Compound **5c**

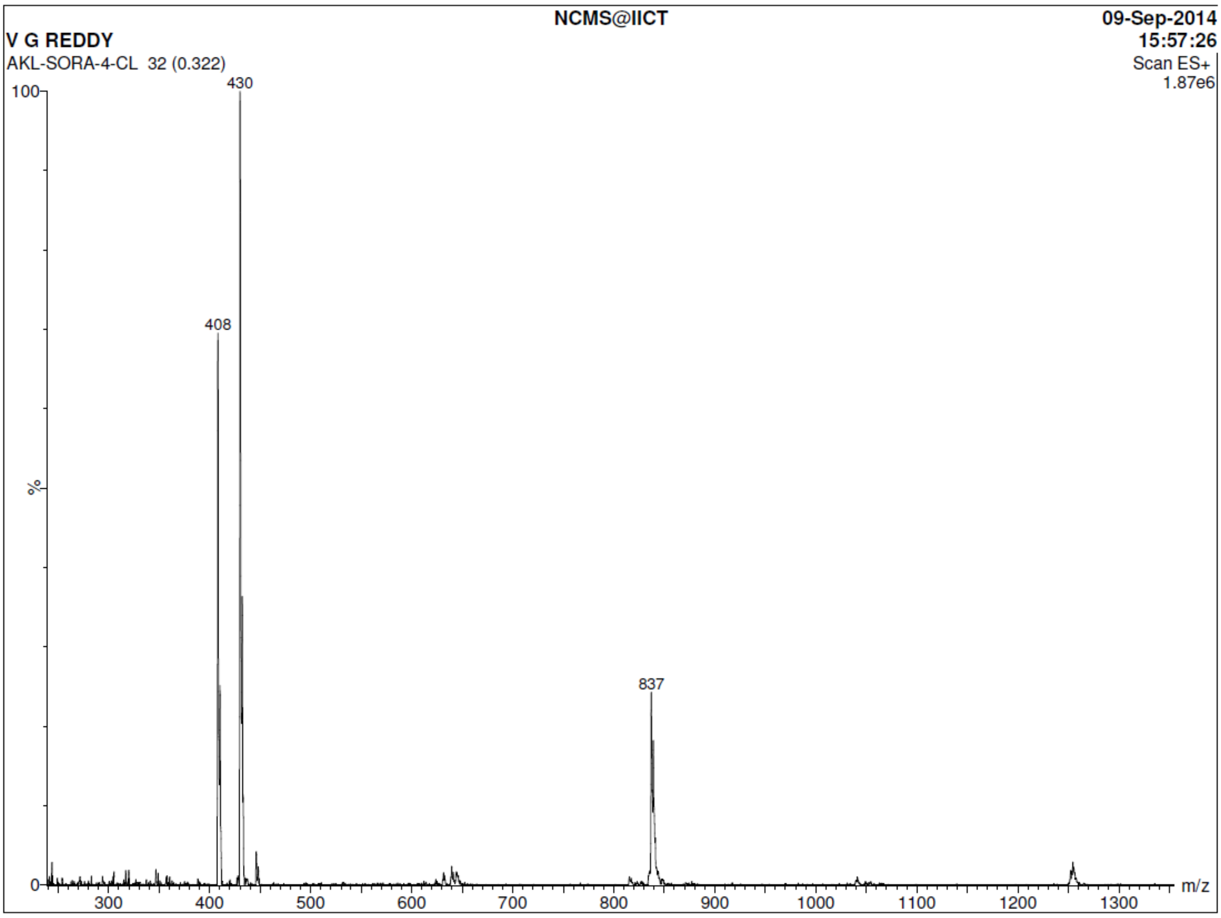

Compound **5c**

National Centre for Mass Spectrometry
CSIR-Indian Institute of Chemical Technology

File Name AKL-SORA-4-CL
Sample Name
Sample ID V-GANGA-REDDY
Date and Time 16-09-14 21:56:13

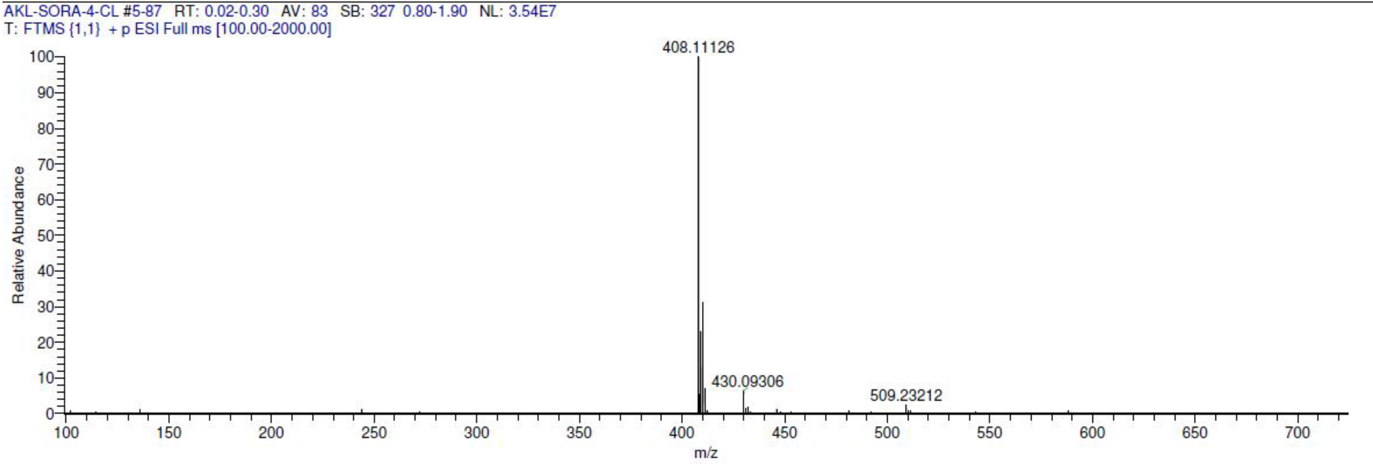

AKL-SORA-4-CL#8-30 RT: 0.03-0.11 AV: 23
T: FTMS {1,1} + p ESI Full ms [100.00-2000.00]
m/z= 383.23-427.77

m/z	Intensity	Relative	Theo. Mass	Delta (ppm)	RDB equiv.	Composition
408.11118	36779492.0	100.00	408.11095	0.57	14.5	$C_{22}H_{19}O_3N_3Cl$

Compound **5d**_Mass

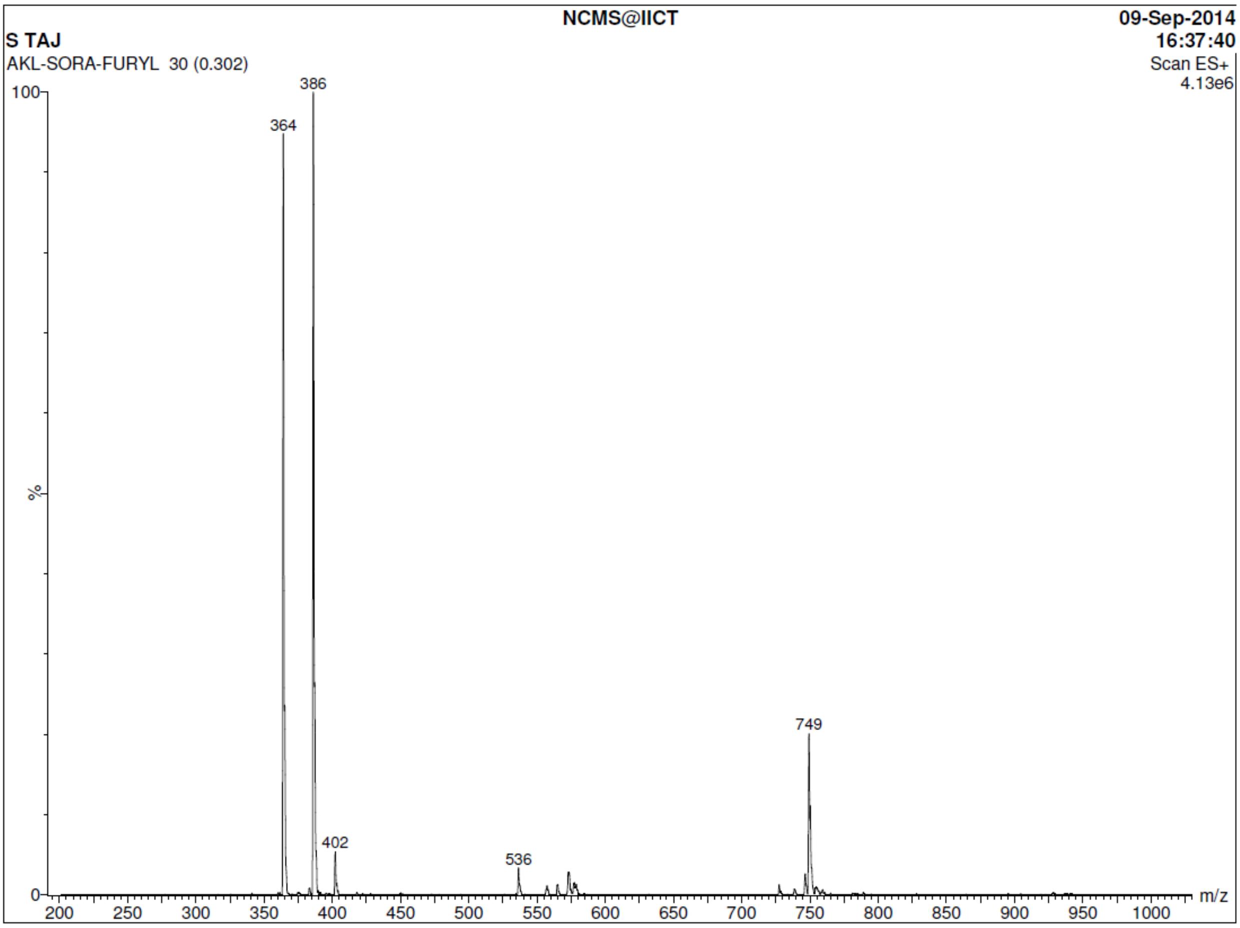

Compound **5d**_HRMS

National Centre for Mass Spectrometry
CSIR-Indian Institute of Chemical Technology

File Name AKL-SORA-FURYL
Sample Name
Sample ID SHAIK-TAJ
Date and Time 16-09-14 19:33:12

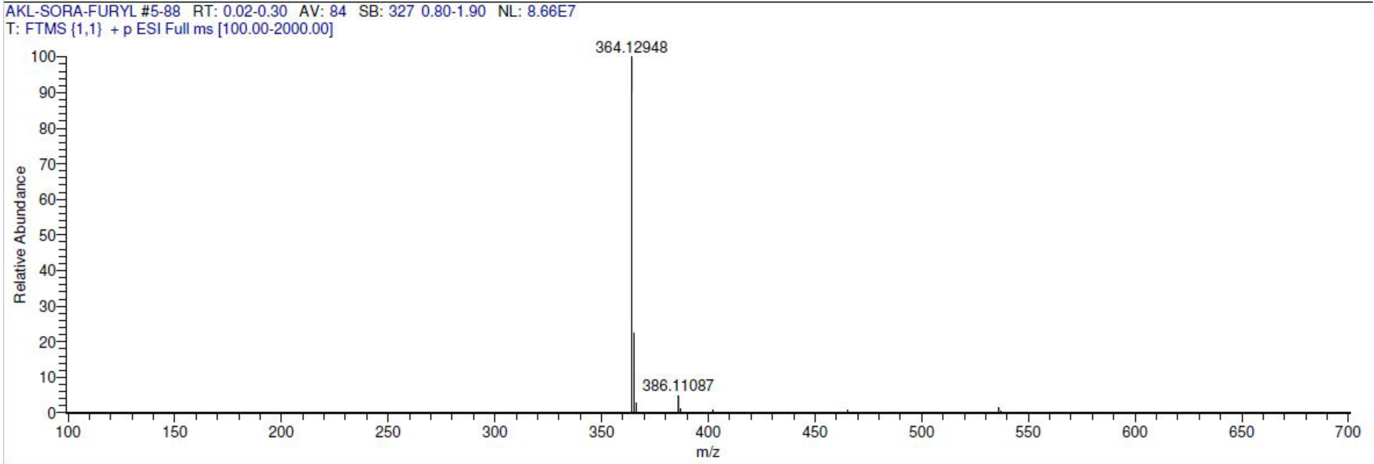

AKL-SORA-FURYL#8-30 RT: 0.03-0.11 AV: 23
T: FTMS {1,1} + p ESI Full ms [100.00-2000.00]
m/z= 351.45-376.38

m/z	Intensity	Relative	Theo. Mass	Delta (ppm)	RDB equiv.	Composition
364.12921	71227512.0	100.00	364.12918	0.09	13.5	$C_{20}H_{18}O_4N_3$

Compound **5e**_Mass

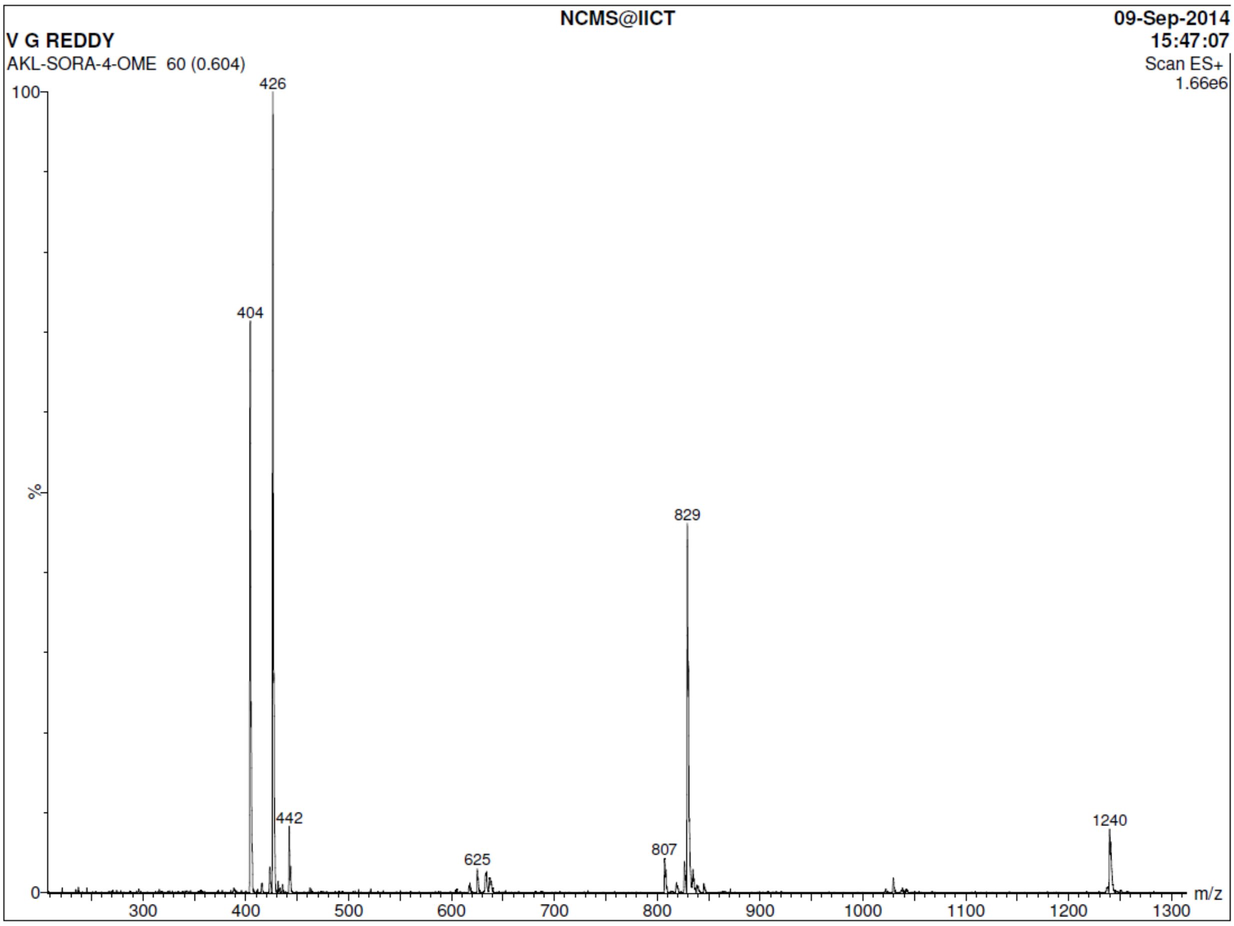

Compound **5e**_HRMS

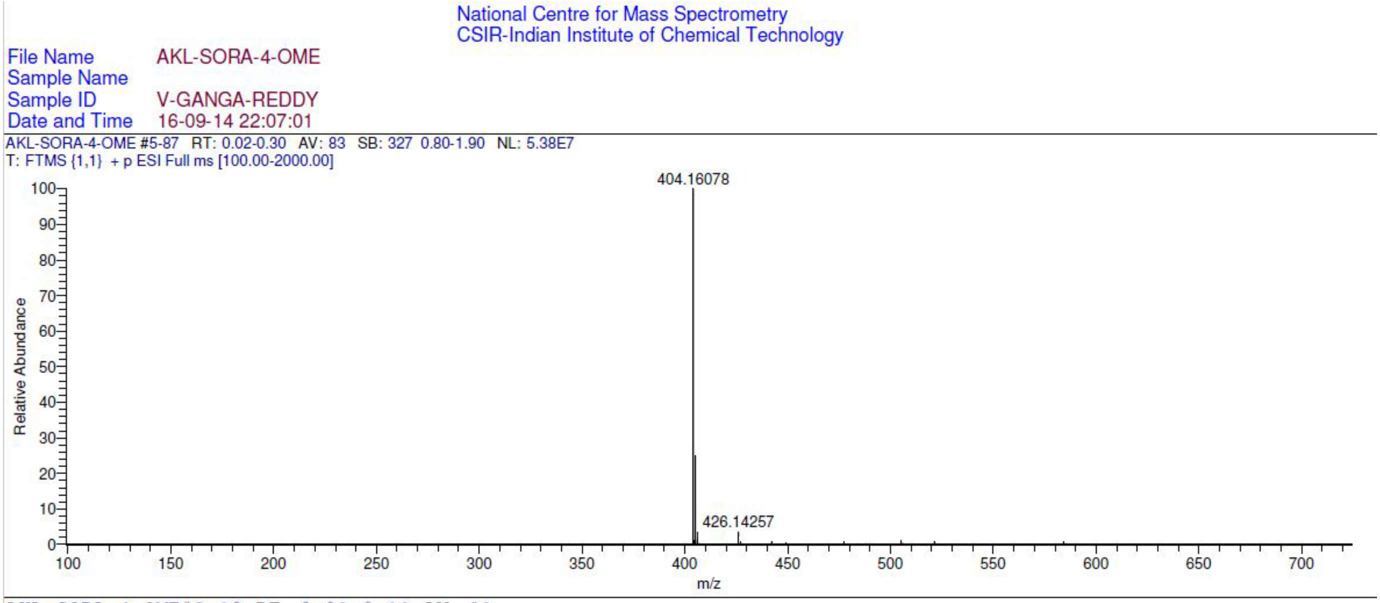

AKL-SORA-4-OME#8-30 RT: 0.03-0.11 AV: 23
T: FTMS {1,1} + p ESI Full ms [100.00-2000.00]
m/z= 387.28-415.62

m/z	Intensity	Relative	Theo. Mass	Delta (ppm)	RDB equiv.	Composition
404.16073	38441436.0	100.00	404.16048	0.60	14.5	$C_{23}H_{22}O_4N_3$

Compound **5f**_Mass

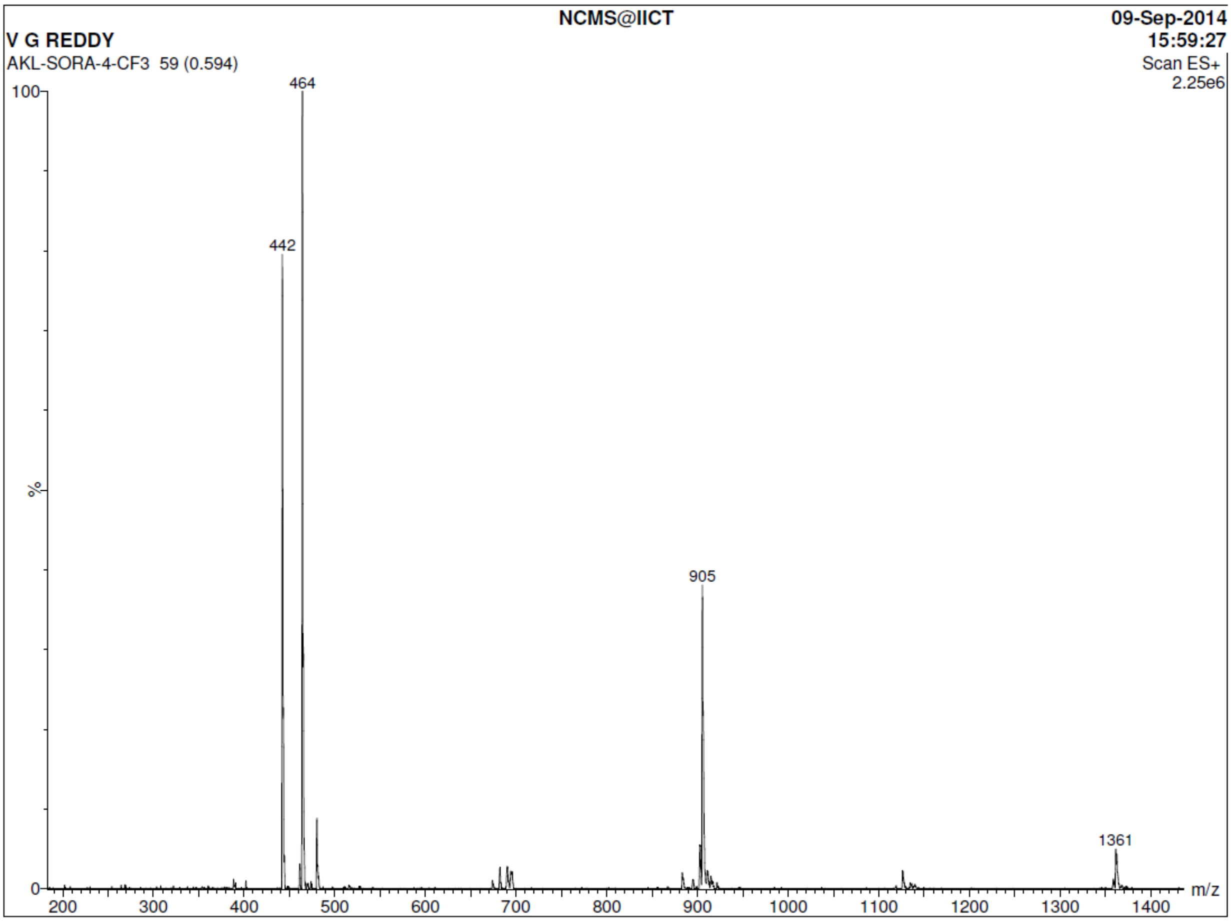

Compound **5f**_HRMS

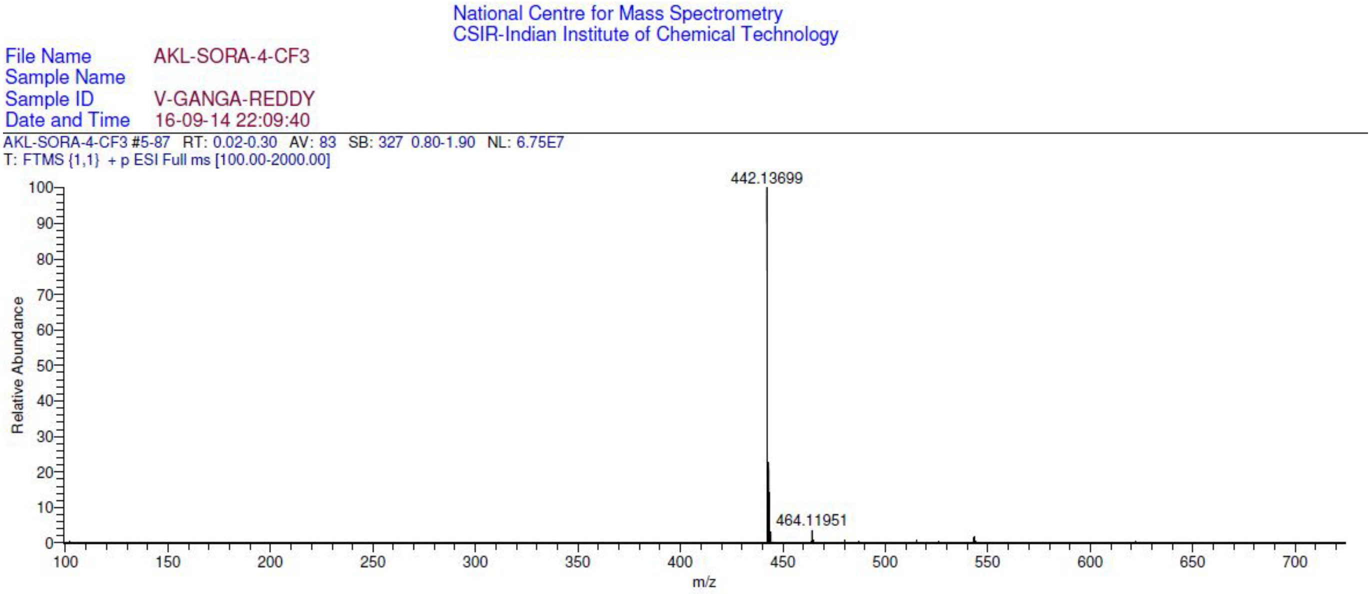

AKL-SORA-4-CF3#8-30 RT: 0.03-0.11 AV: 23
T: FTMS {1,1} + p ESI Full ms [100.00-2000.00]

m/z	Intensity	Relative	Theo. Mass	Delta (ppm)	RDB equiv.	Composition
442.13691	58738184.0	100.00	442.13730	-0.90	14.5	$C_{23}H_{19}O_3N_3F_3$

Compound **5g**_Mass

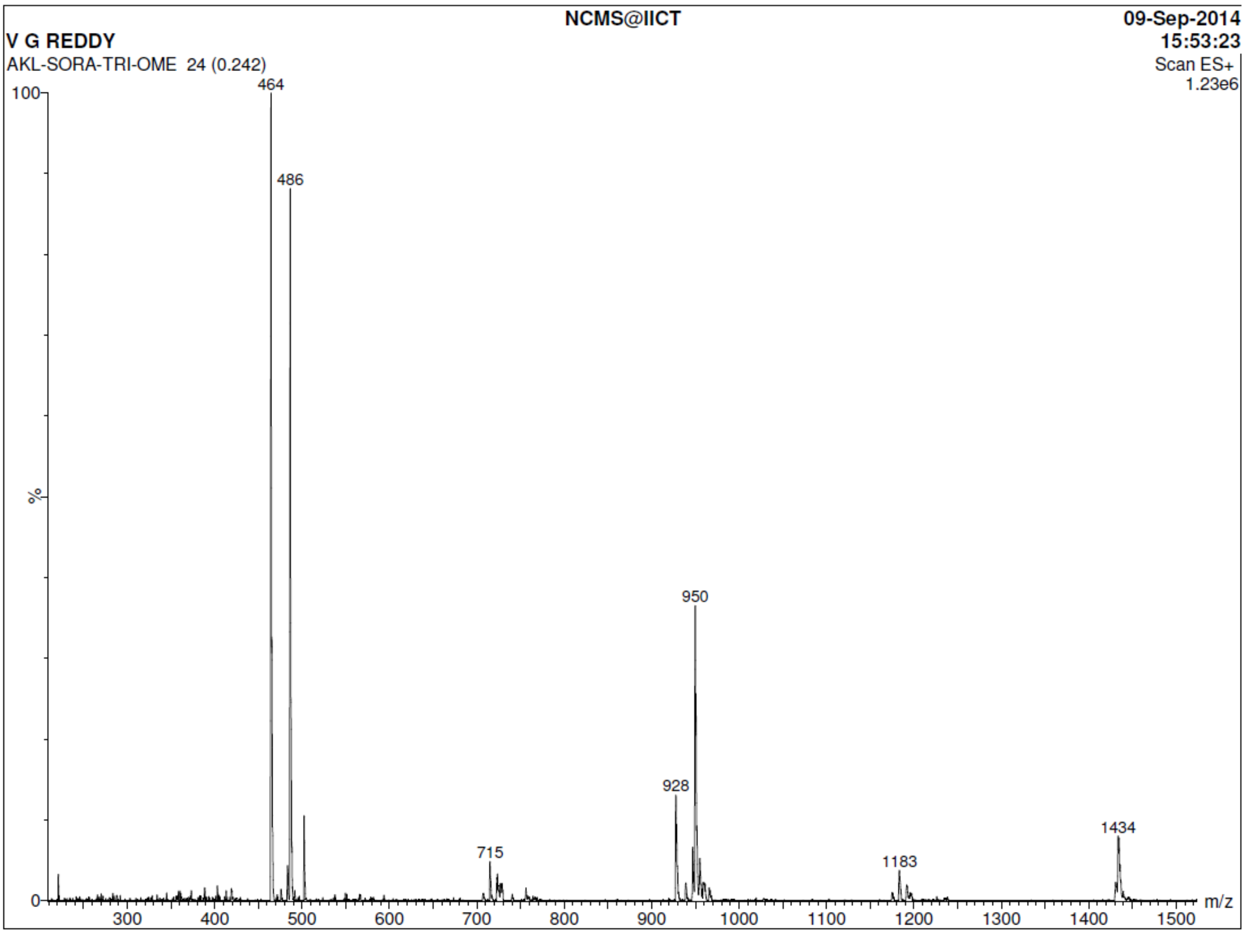

Compound **5g**_HRMS

National Centre for Mass Spectrometry
CSIR-Indian Institute of Chemical Technology

File Name AKL-SORA-TRI-OME
Sample Name
Sample ID V-GANGA-REDDY
Date and Time 16-09-14 21:45:19

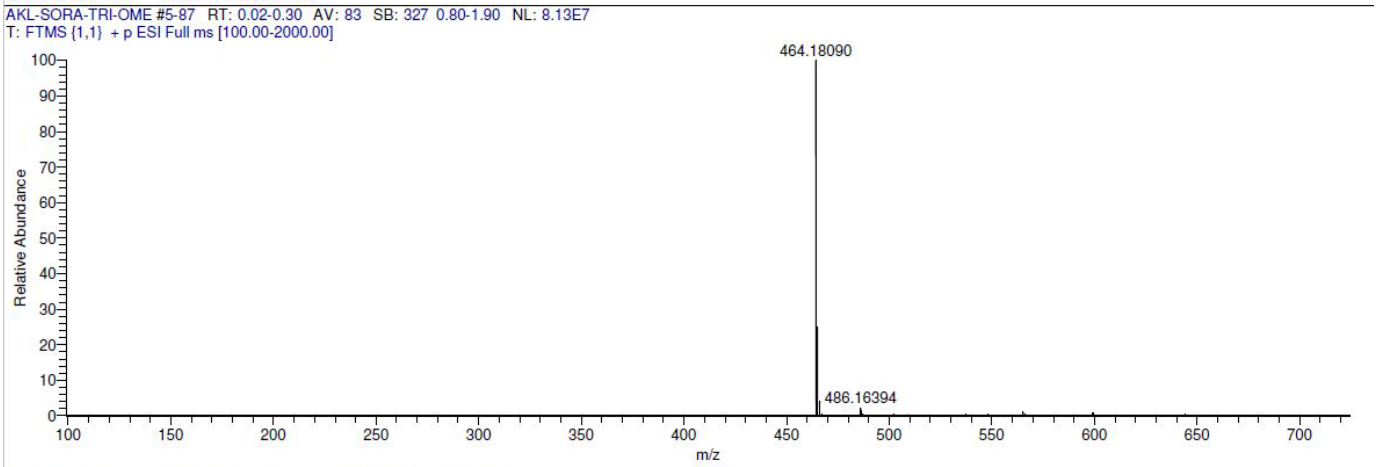

AKL-SORA-TRI-OME#8-30 RT: 0.03-0.11 AV: 23
T: FTMS {1,1} + p ESI Full ms [100.00-2000.00]
m/z= 441.53-485.66

m/z	Intensity	Relative	Theo. Mass	Delta (ppm)	RDB equiv.	Composition
464.18082	77838232.0	100.00	464.18161	-1.70	14.5	$C_{25}H_{26}O_6N_3$

Compound **5h**_Mass

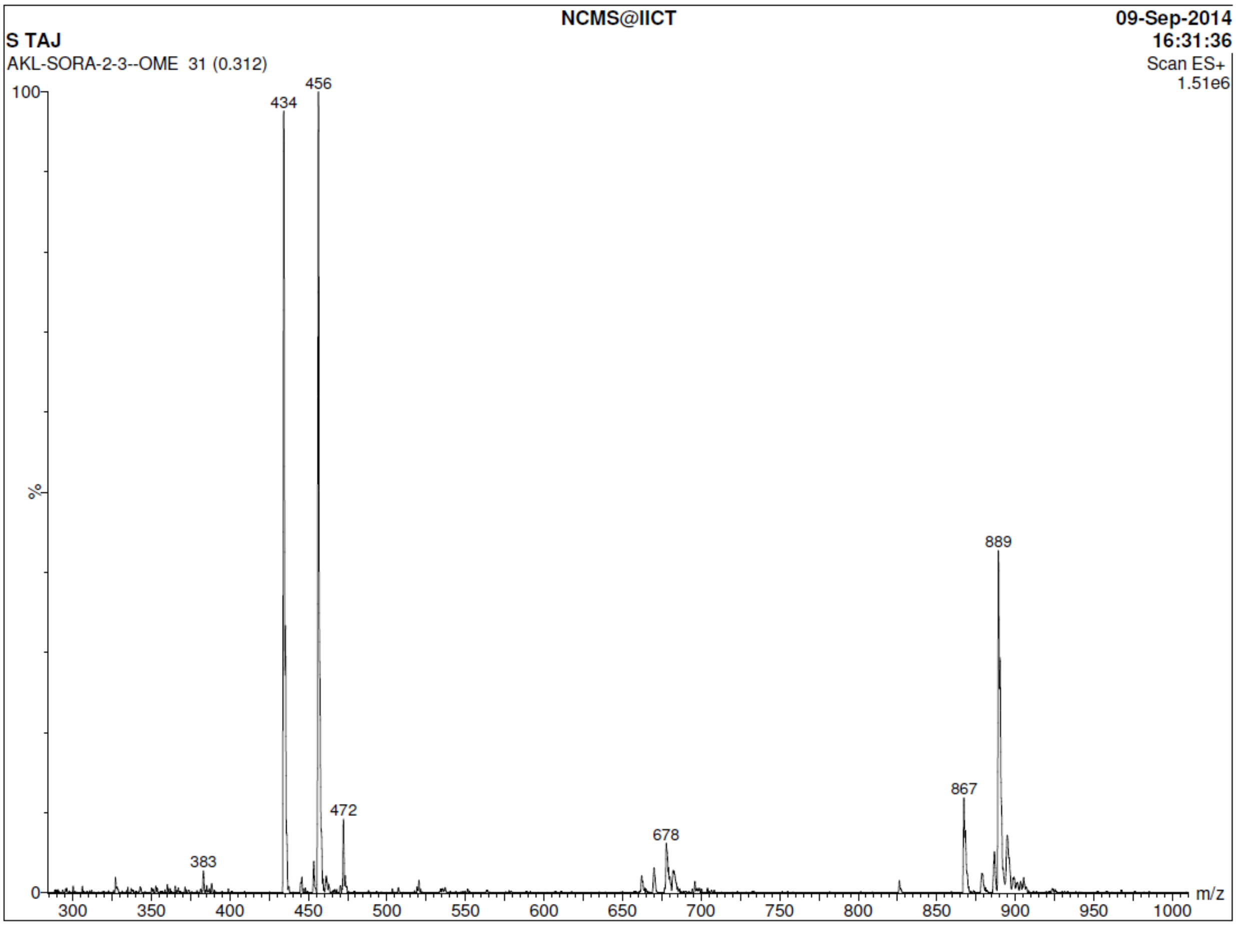

Compound **5h**_HRMS

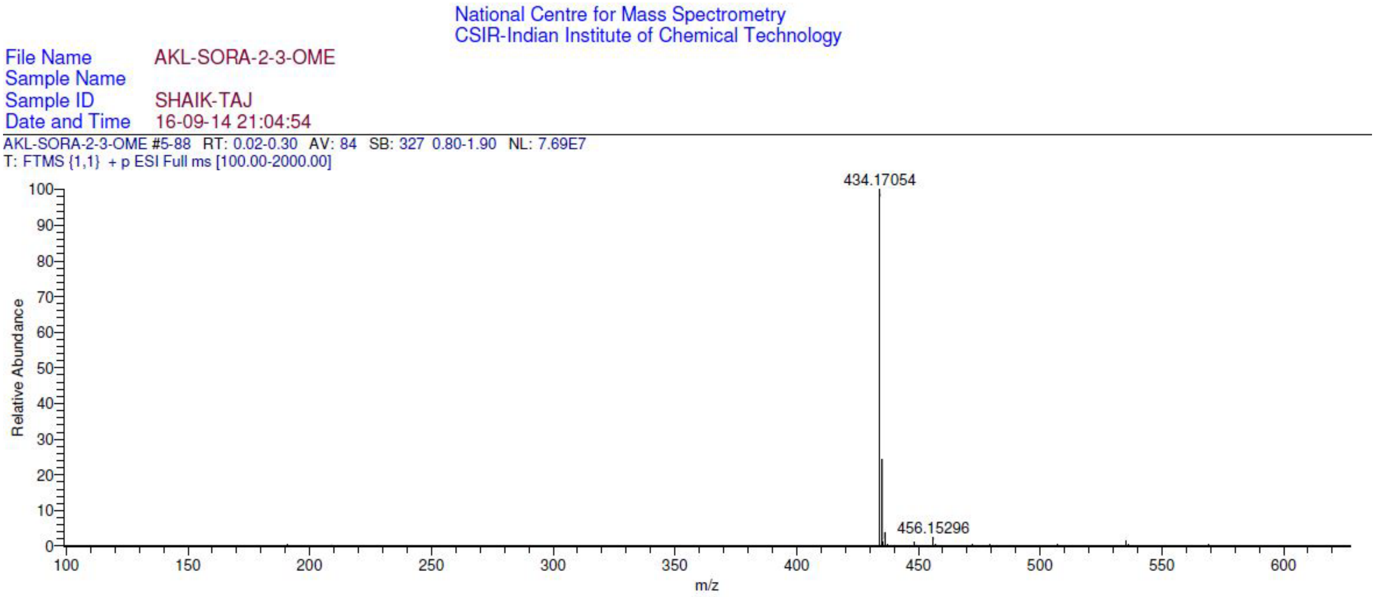

AKL-SORA-2-3-OME#8-30 RT: 0.03-0.11 AV: 23
T: FTMS {1,1} + p ESI Full ms [100.00-2000.00]
m/z= 406.20-448.28

m/z	Intensity	Relative	Theo. Mass	Delta (ppm)	RDB equiv.	Composition
434.17043	59056708.0	100.00	434.17105	-1.42	14.5	$C_{24}H_{24}O_5N_3$

Compound **5i**_Mass

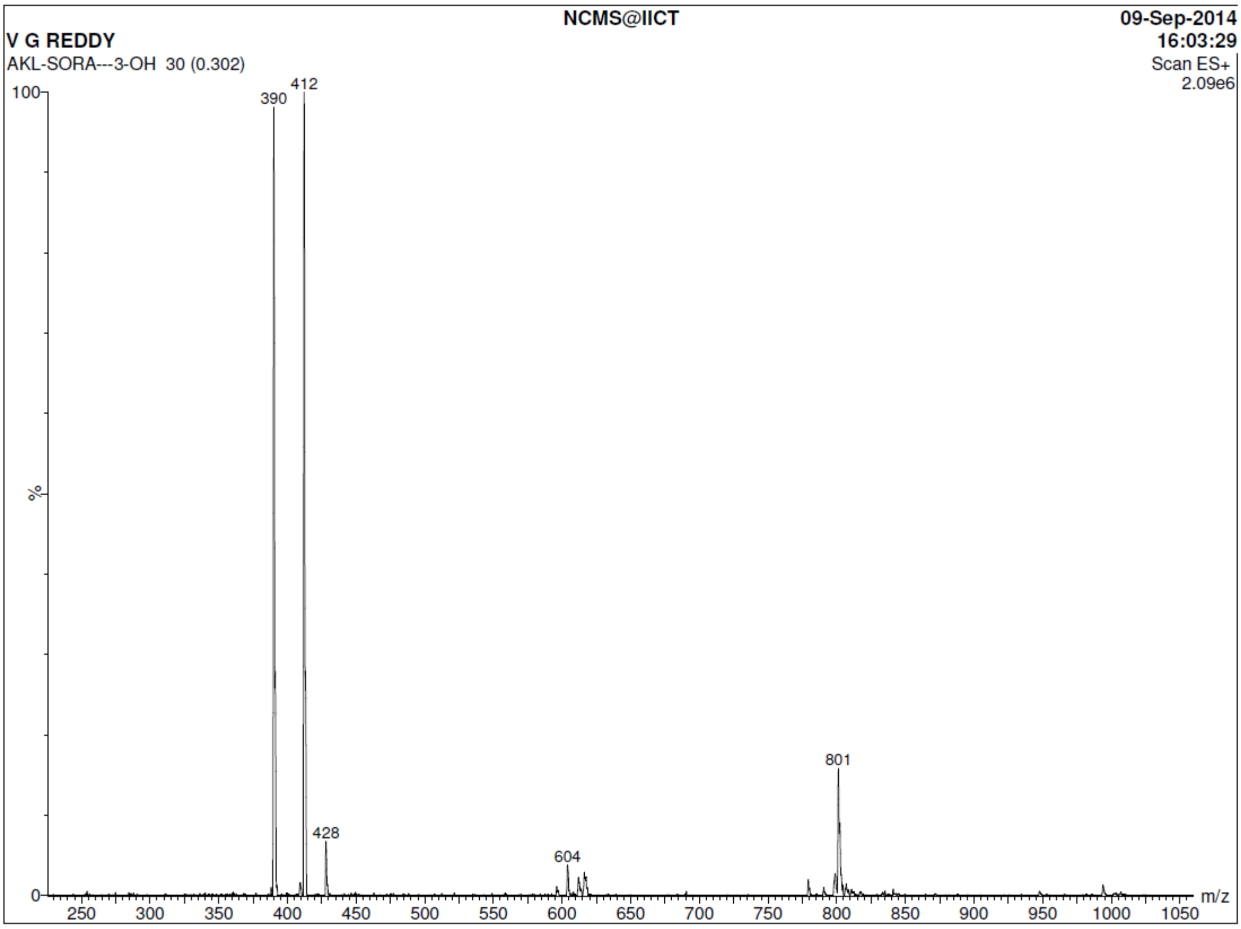

Compound **5i**_HRMS

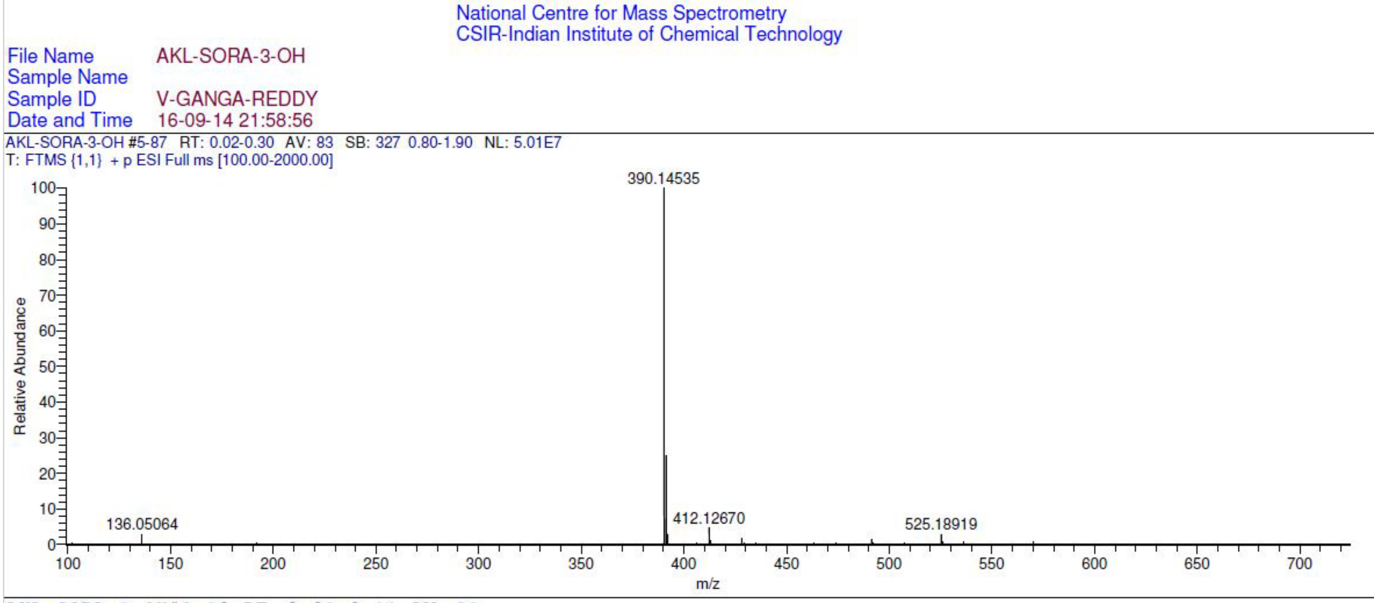

AKL-SORA-3-OH#8-30 RT: 0.03-0.11 AV: 23
T: FTMS {1,1} + p ESI Full ms [100.00-2000.00]

m/z	Intensity	Relative	Theo. Mass	Delta (ppm)	RDB equiv.	Composition
390.14519	48580428.0	100.00	390.14483	0.90	14.5	$C_{22}H_{20}O_4N_3$

Compound **5j**_Mass

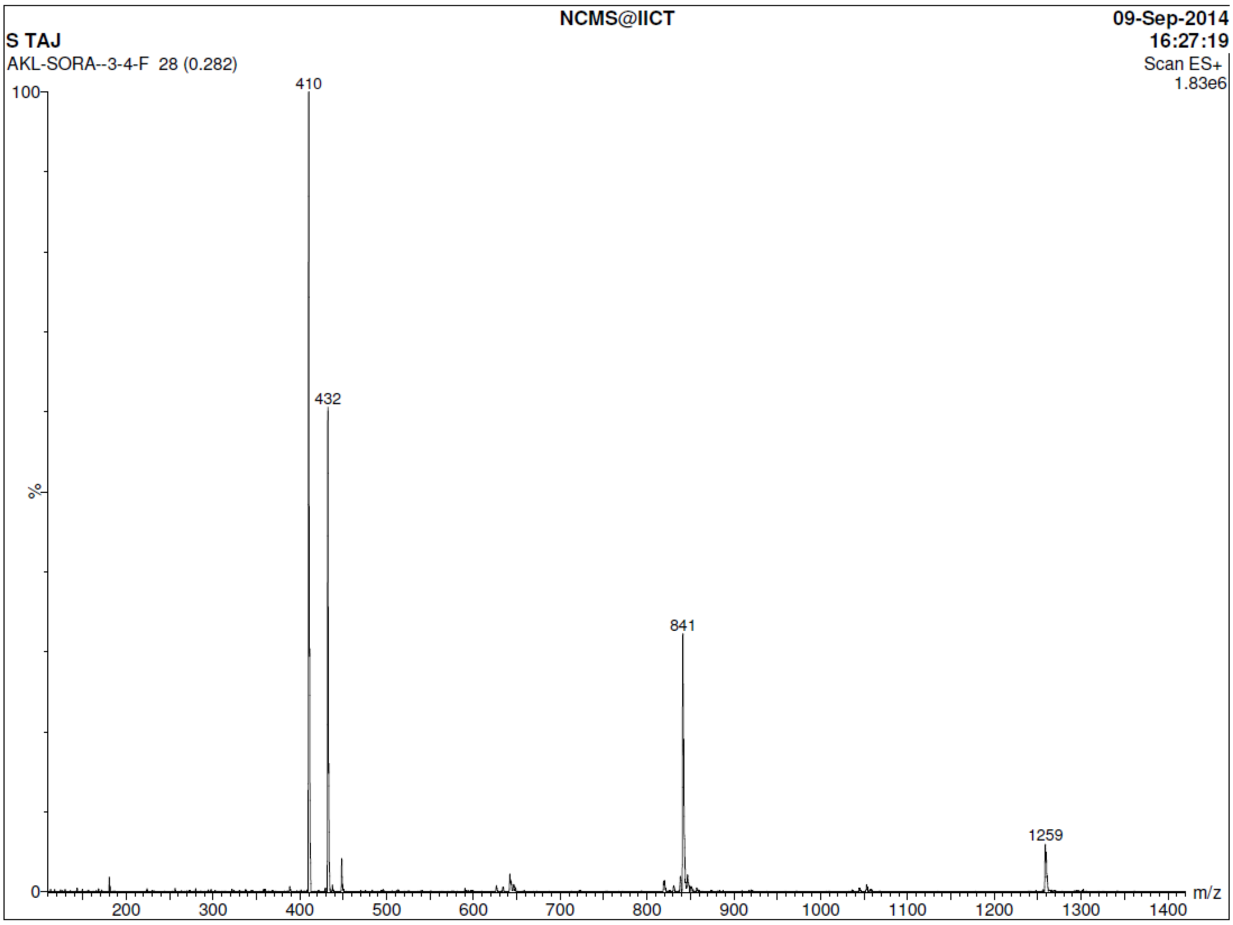

Compound **5j**_HRMS

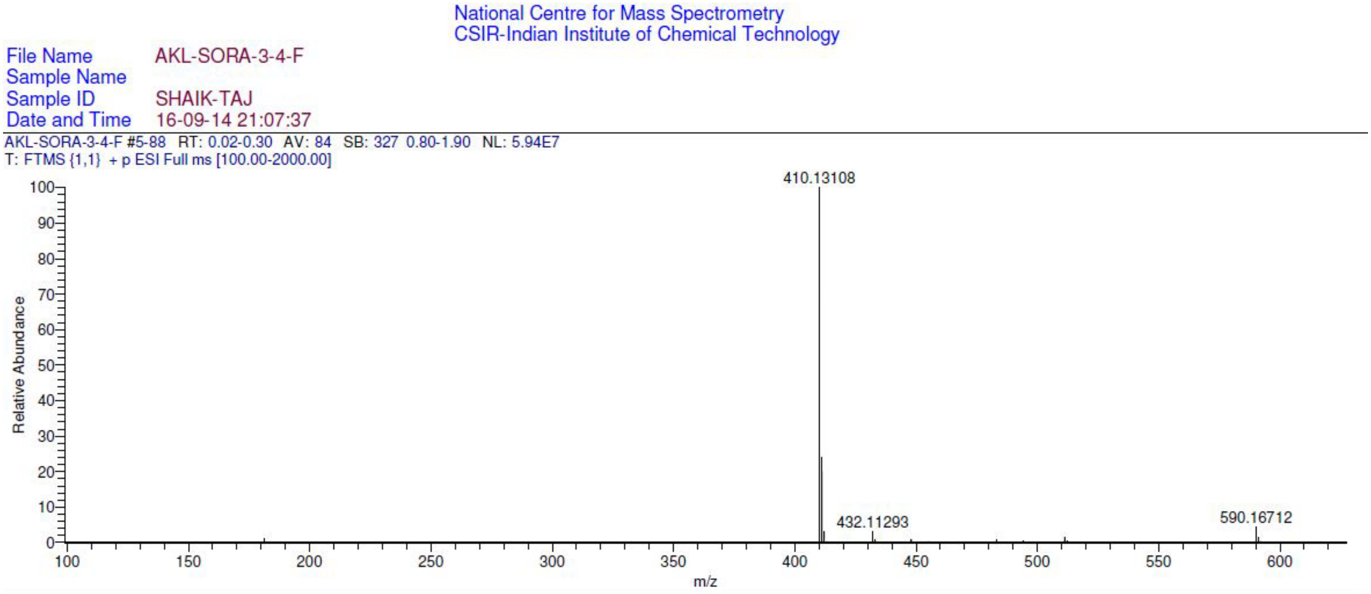

AKL-SORA-3-4-F#8-30 RT: 0.03-0.11 AV: 23

T: FTMS {1,1} + p ESI Full ms [100.00-2000.00]

m/z= 392.52-422.62

m/z	Intensity	Relative	Theo. Mass	Delta (ppm)	RDB equiv.	Composition
410.13092	45598428.0	100.00	410.13107	-0.39	14.5	$C_{22}H_{18}O_3N_3F_2$

Compound **5k**_Mass

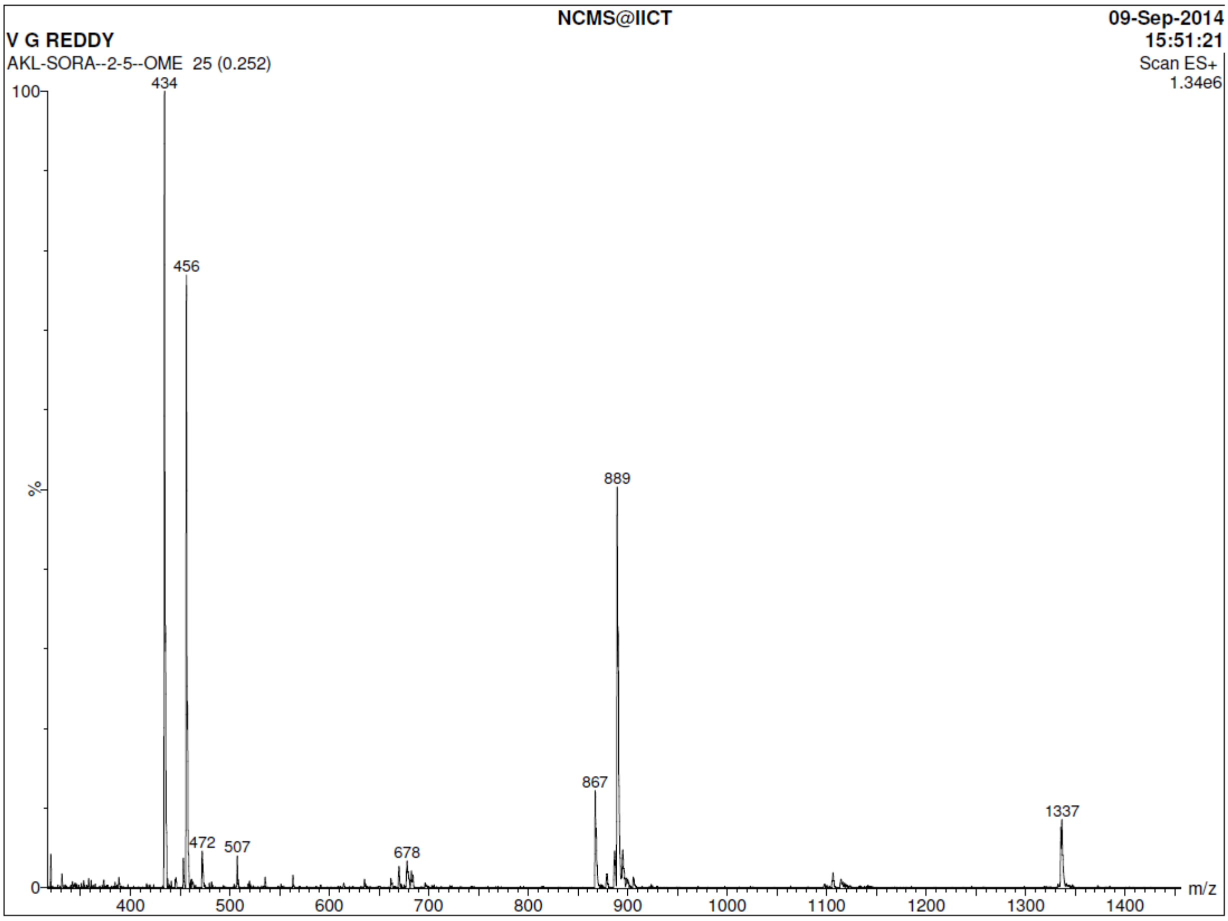

Compound **5k**_HRMS

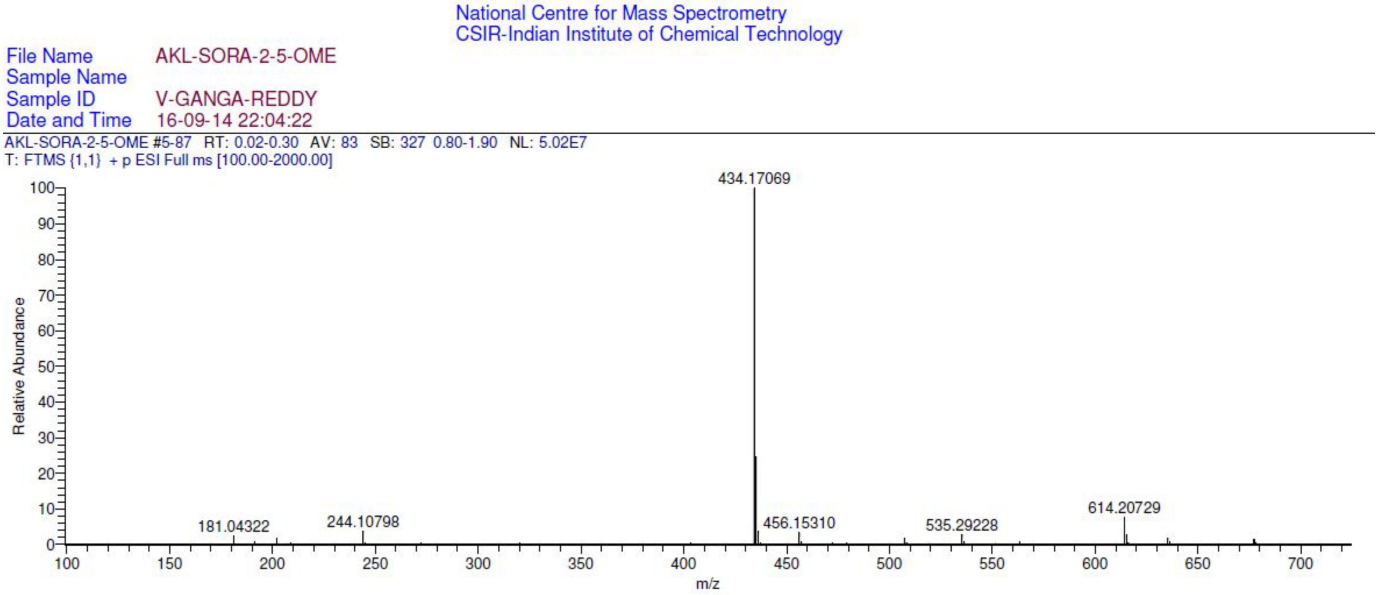

AKL-SORA-2-5-OME#8-30 RT: 0.03-0.11 AV: 23
T: FTMS {1,1} + p ESI Full ms [100.00-2000.00]
m/z= 395.37-462.58

m/z	Intensity	Relative	Theo. Mass	Delta (ppm)	RDB equiv.	Composition
434.17073	36381256.0	100.00	434.17105	-0.73	14.5	$C_{24}H_{24}O_5N_3$

Compound **5l**_Mass

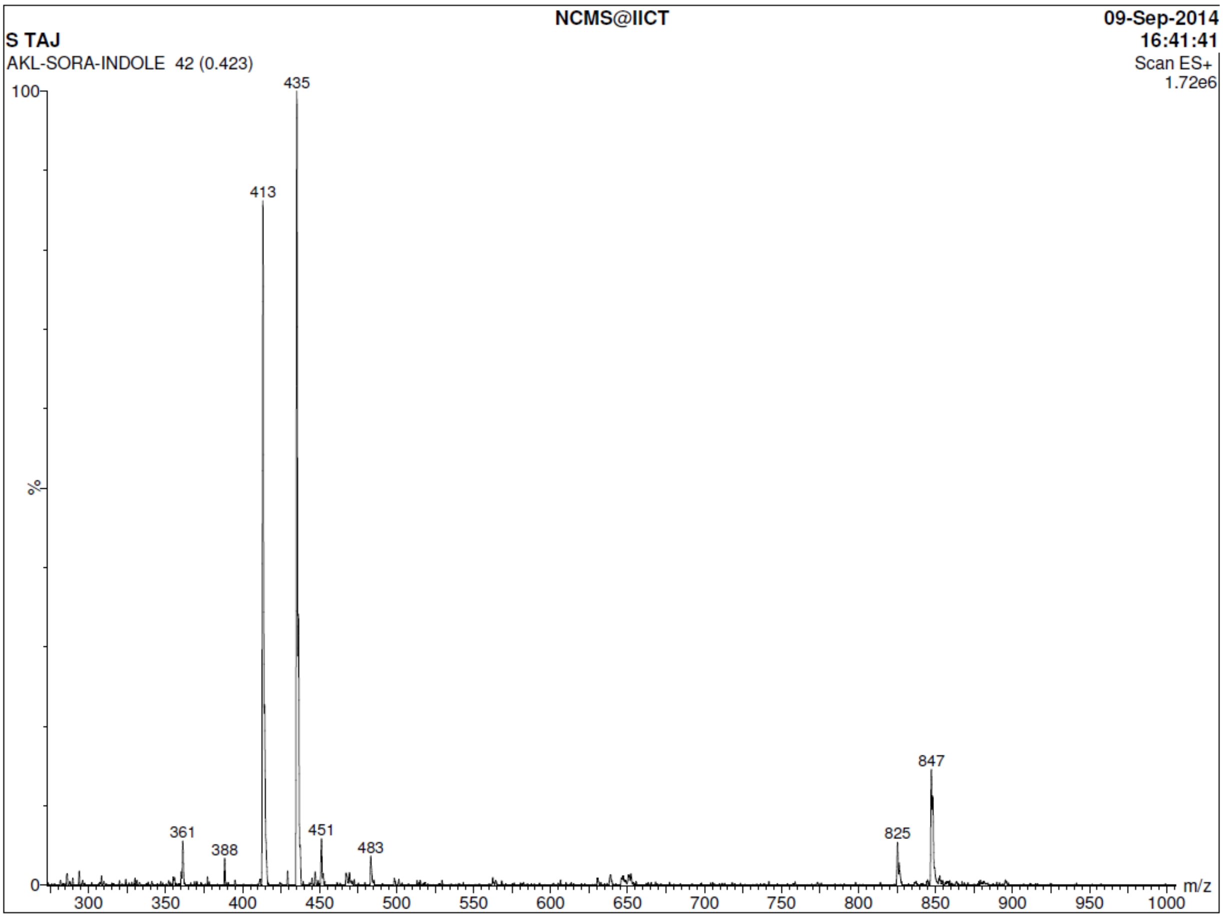

Compound **5l**_HRMS

National Centre for Mass Spectrometry
CSIR-Indian Institute of Chemical Technology

File Name AKL-SORA-INDOLE
Sample Name
Sample ID SHAIK-TAJ
Date and Time 16-09-14 21:42:36

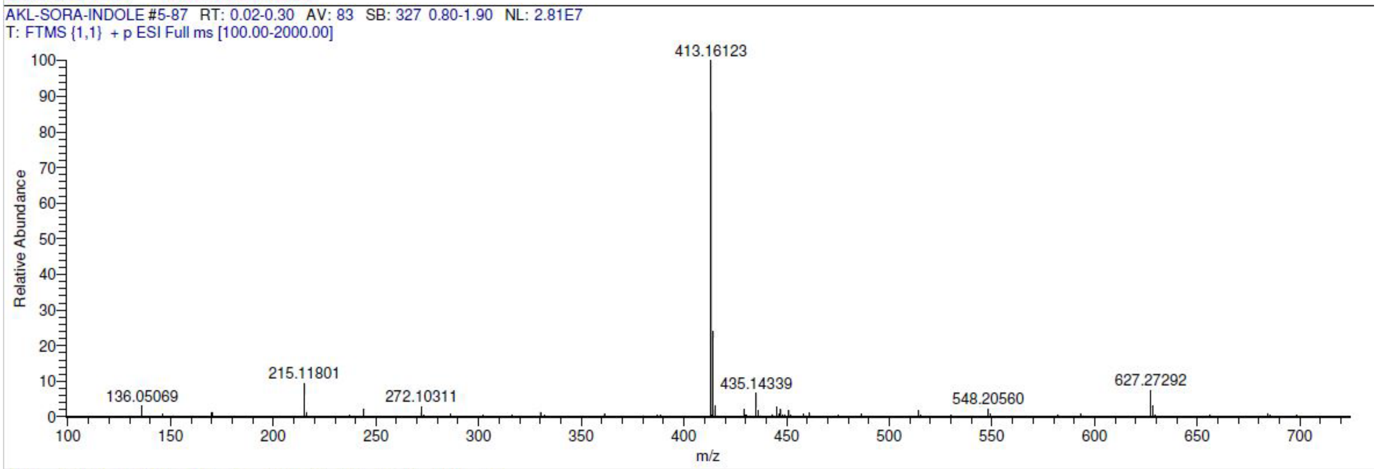

AKL-SORA-INDOLE#8-30 RT: 0.03-0.11 AV: 23
T: FTMS {1,1} + p ESI Full ms [100.00-2000.00]
m/z= 403.07-430.19

m/z	Intensity	Relative	Theo. Mass	Delta (ppm)	RDB equiv.	Composition
413.16121	30873490.0	100.00	413.16082	0.95	16.5	$C_{24}H_{21}O_3N_4$

Compound **5m**_Mass

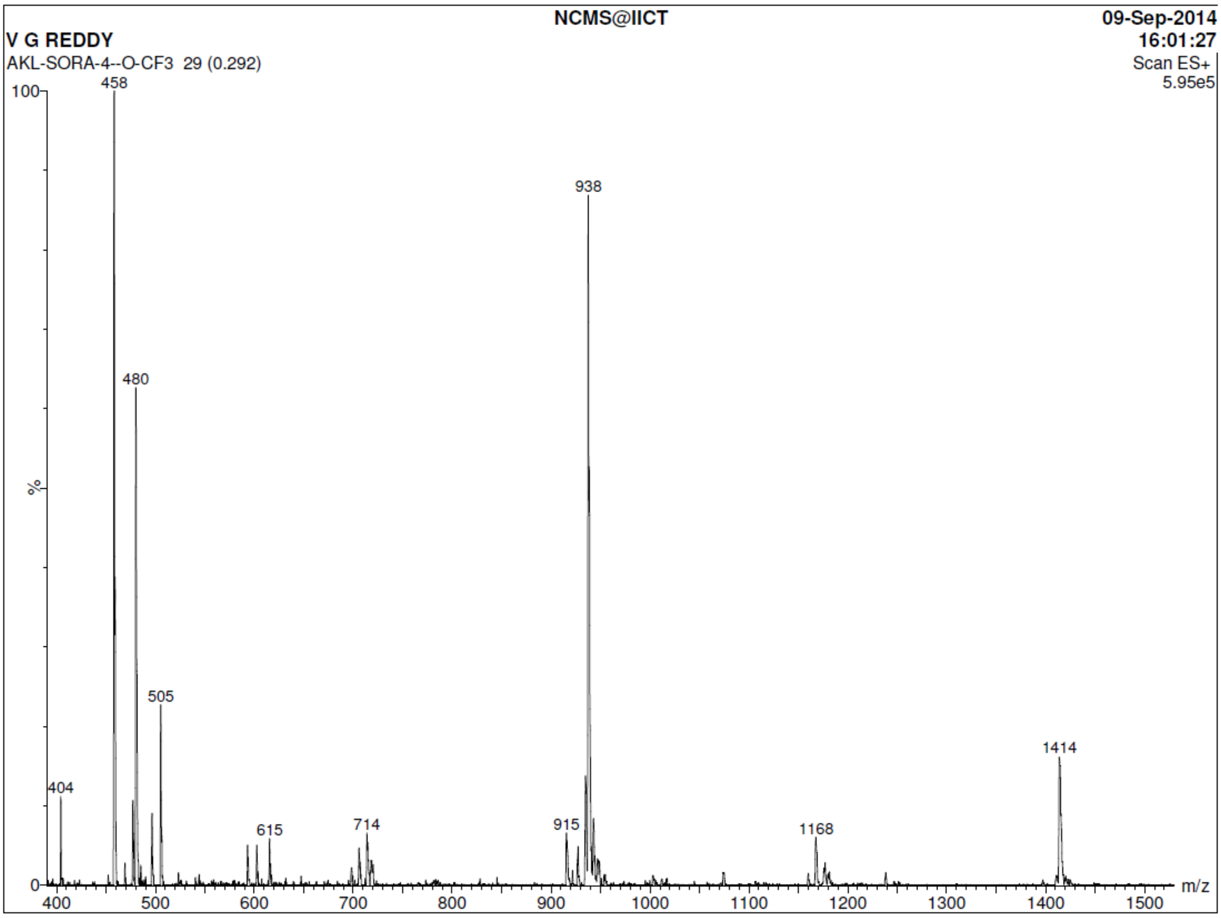

Compound **5m**_HRMS

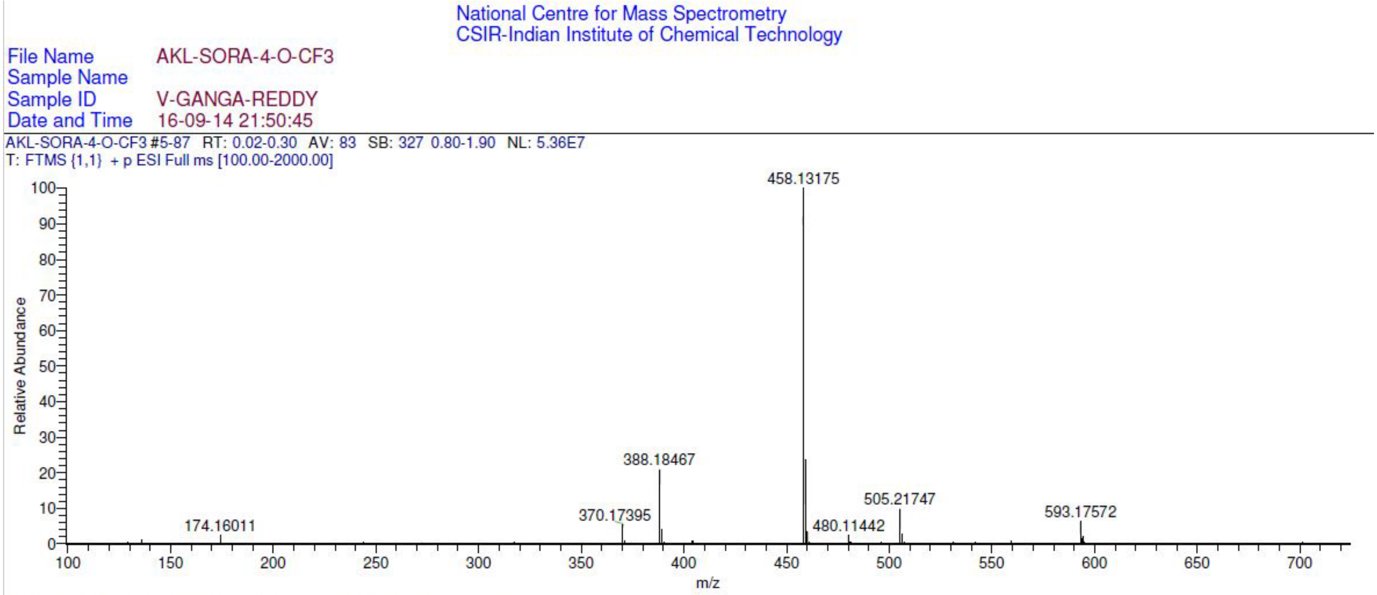

AKL-SORA-4-O-CF3#8-30 RT: 0.03-0.11 AV: 23
T: FTMS {1,1} + p ESI Full ms [100.00-2000.00]
m/z= 424.93-474.33

m/z	Intensity	Relative	Theo. Mass	Delta (ppm)	RDB equiv.	Composition
458.13205	35102960.0	100.00	458.13222	-0.36	14.5	$C_{23}H_{19}O_4N_3F_3$

Compound **5n**_Mass

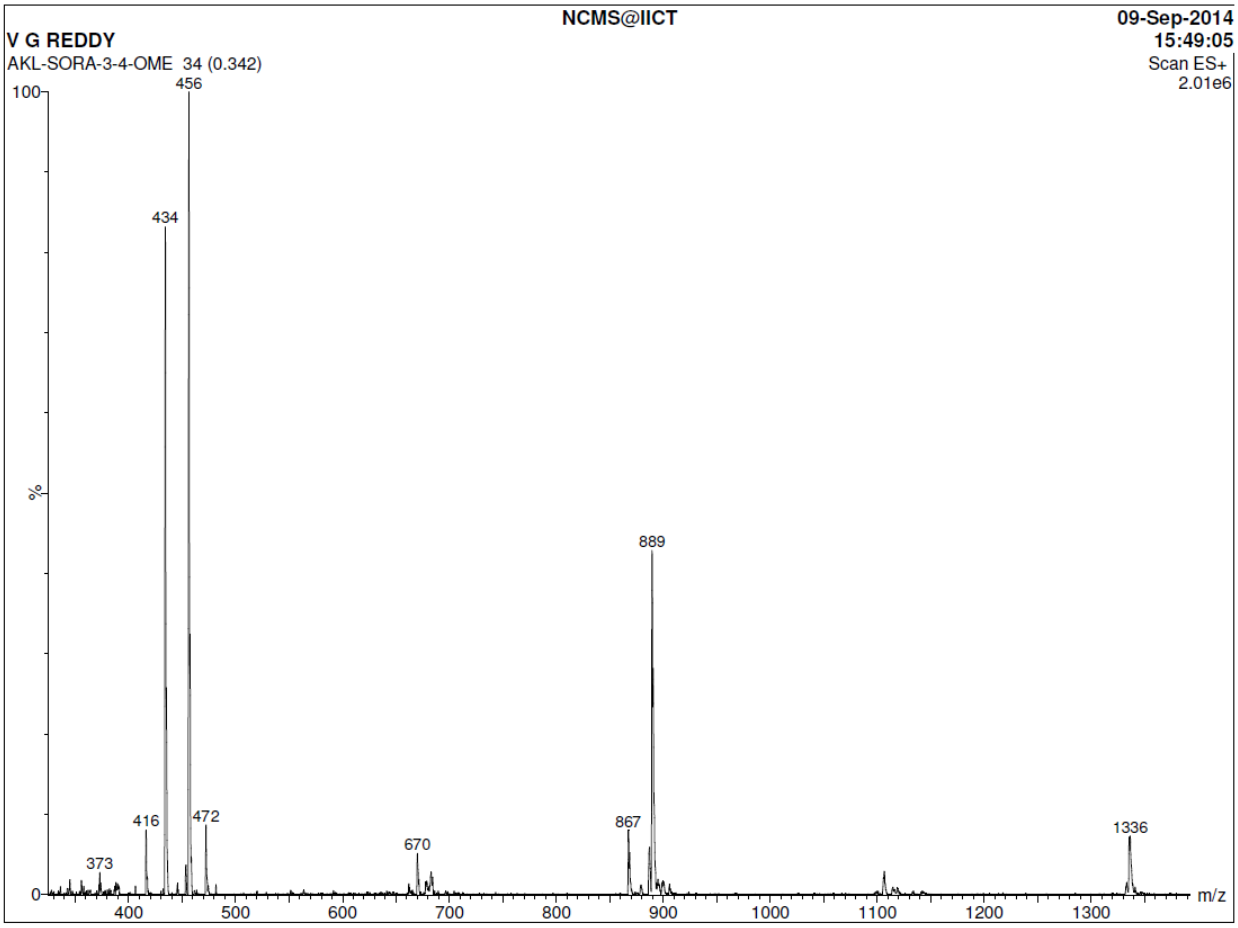

Compound **5n**_HRMS

National Centre for Mass Spectrometry
CSIR-Indian Institute of Chemical Technology

File Name AKL-SORA-3-4-OME
Sample Name
Sample ID V-GANGA-REDDY
Date and Time 16-09-14 22:01:39

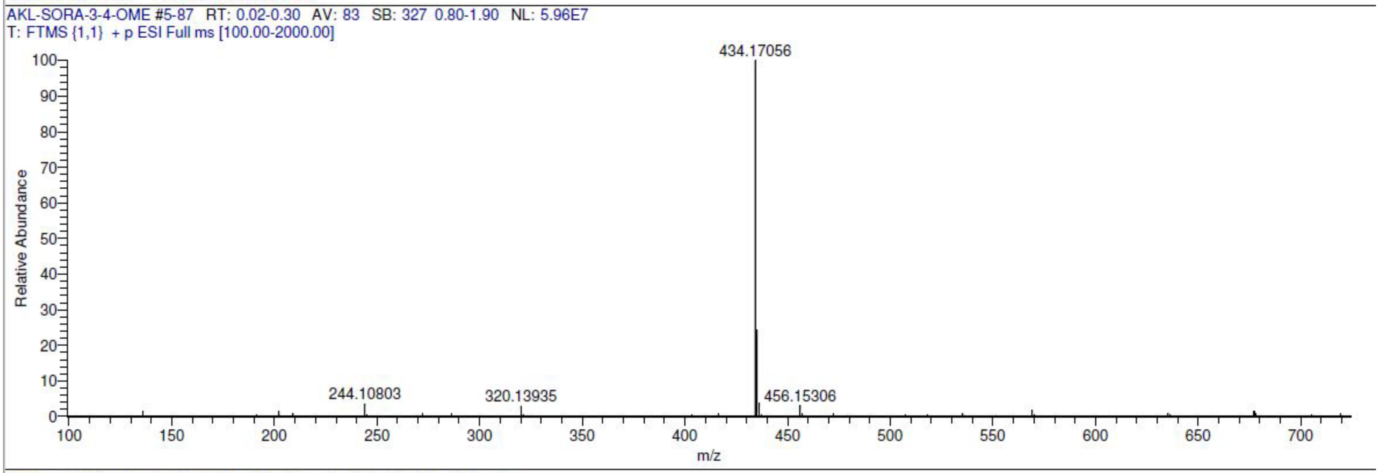

AKL-SORA-3-4-OME#8-30 RT: 0.03-0.11 AV: 23
T: FTMS {1,1} + p ESI Full ms [100.00-2000.00]
m/z= 425.34-441.53

m/z	Intensity	Relative	Theo. Mass	Delta (ppm)	RDB equiv.	Composition
434.17056	51398916.0	100.00	434.17105	-1.13	14.5	$C_{24}H_{24}O_5N_3$

Compound **5o**_Mass

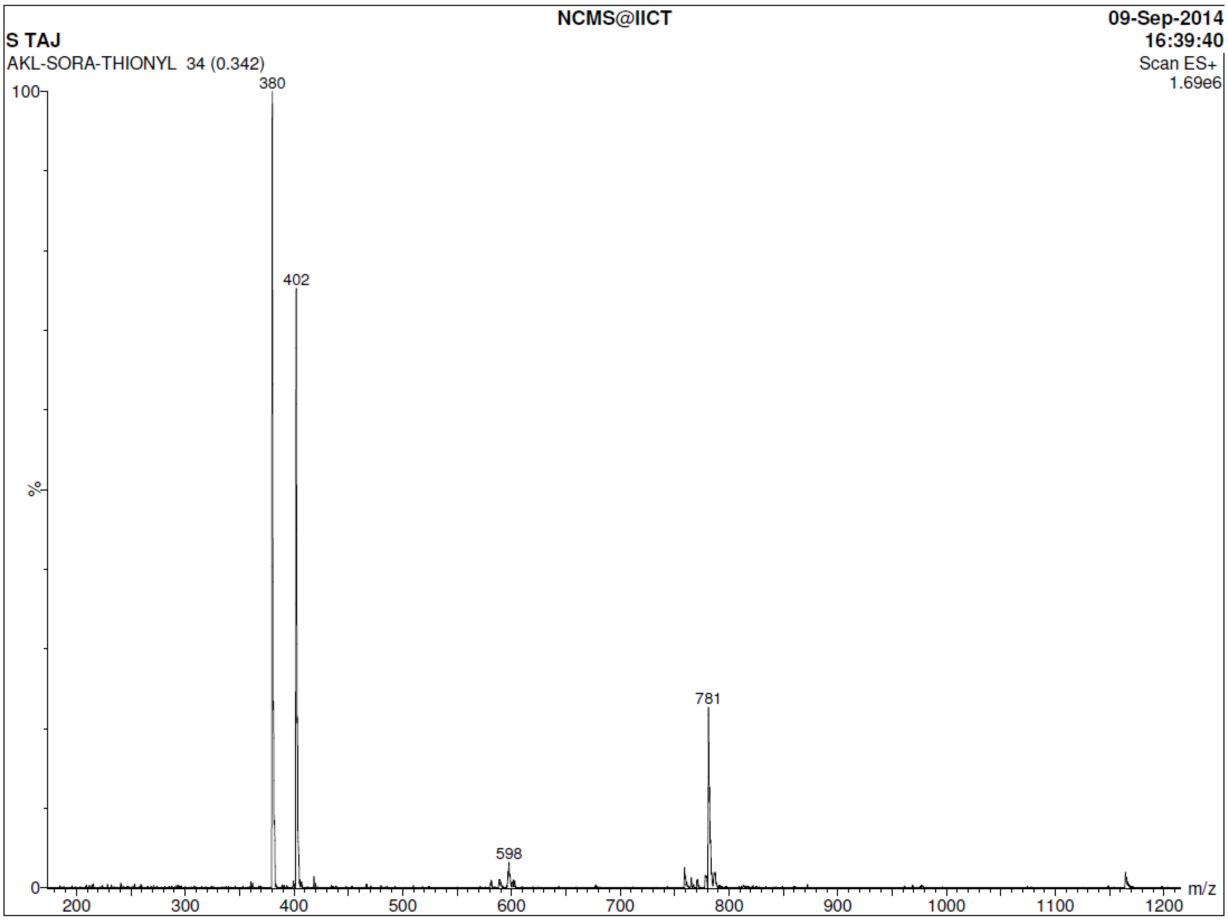

Compound **5o**_HRMS

National Centre for Mass Spectrometry
CSIR-Indian Institute of Chemical Technology

File Name AKL-SORA-THIONYL
Sample Name
Sample ID SHAIK-TAJ
Date and Time 16-09-14 21:39:51

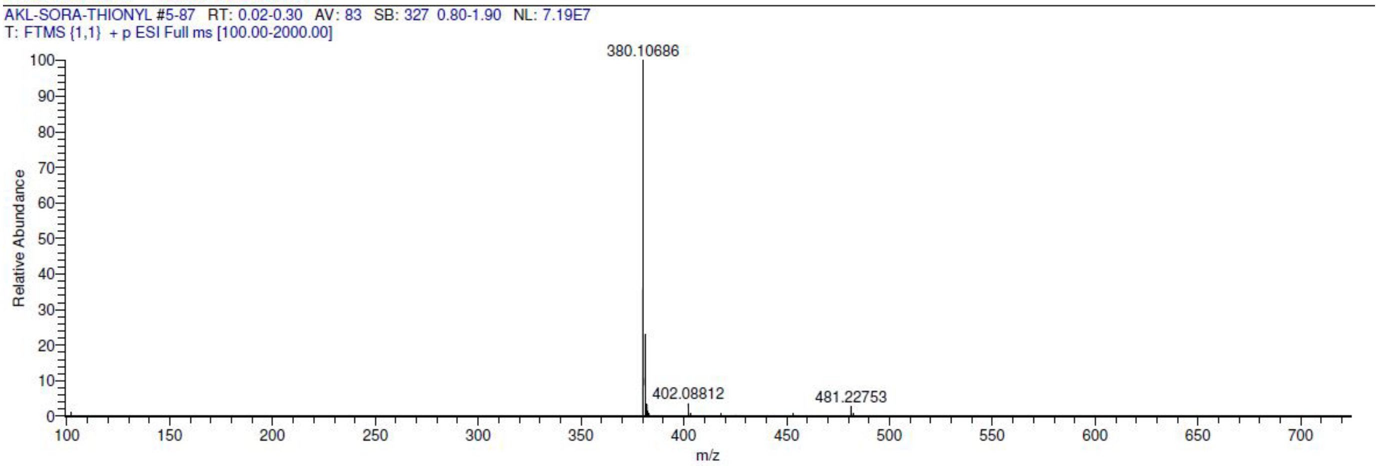

AKL-SORA-THIONYL#8-30 RT: 0.03-0.11 AV: 23
T: FTMS {1,1} + p ESI Full ms [100.00-2000.00]
m/z= 368.65-396.18

m/z	Intensity	Relative	Theo. Mass	Delta (ppm)	RDB equiv.	Composition
380.10673	52163220.0	100.00	380.10634	1.03	13.5	$C_{20}H_{18}O_3N_3S$

Compound **5p**_Mass

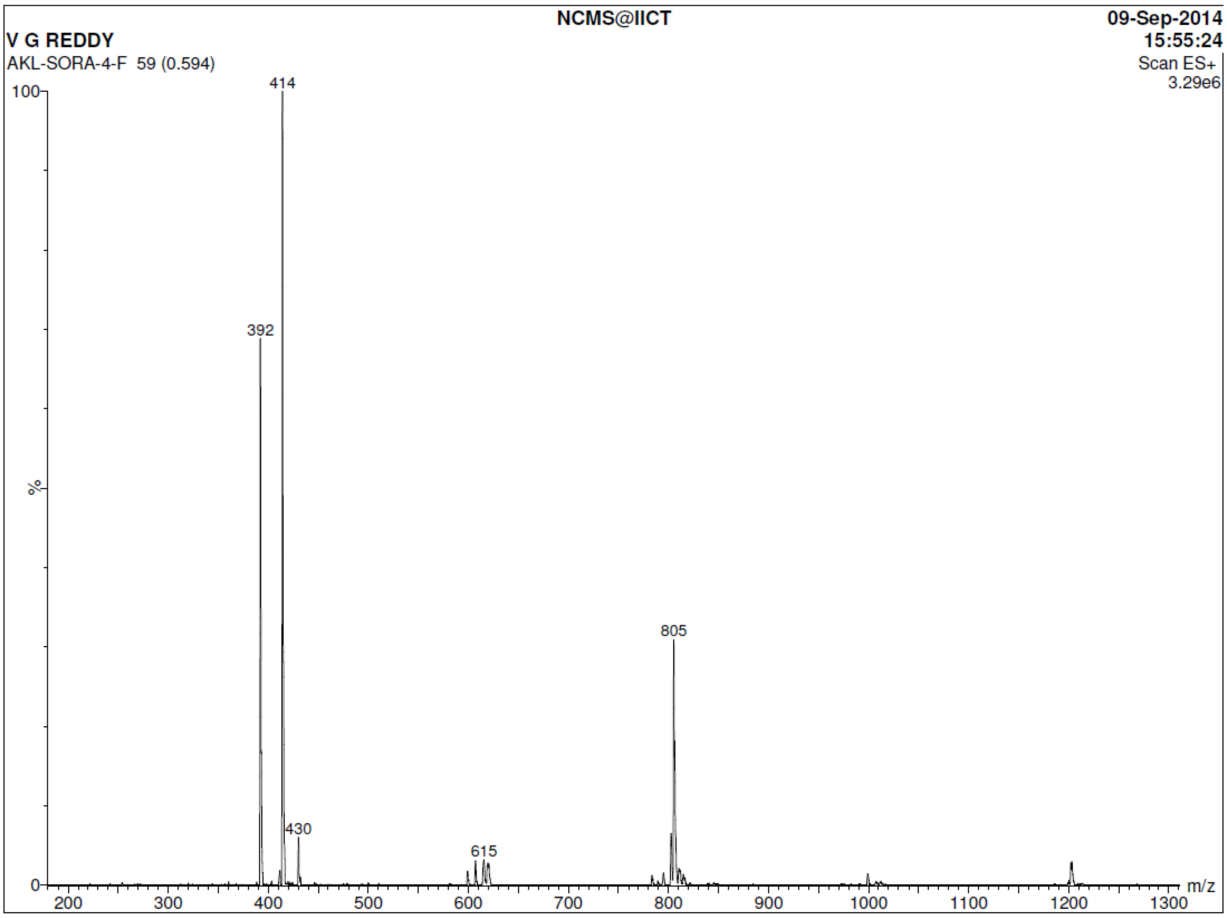

Compound **5p**_HRMS

National Centre for Mass Spectrometry
CSIR-Indian Institute of Chemical Technology

File Name C:\IICT HRMS-03.09.2014\...\AKL-SORA-4-F
Sample Name
Sample ID V-GANGA-REDDY
Date and Time 16-09-14 21:53:30

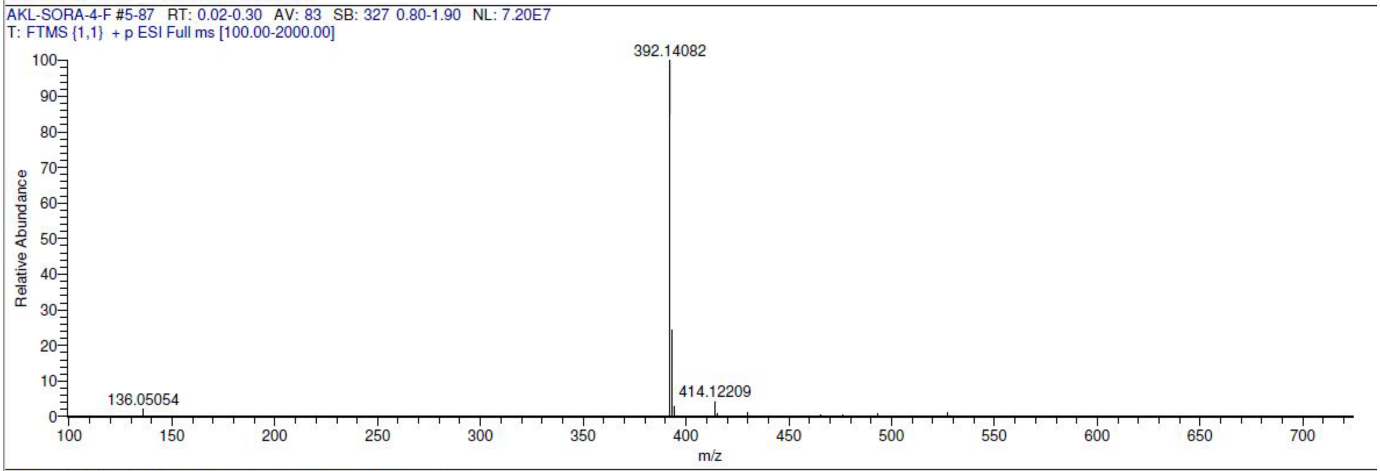

AKL-SORA-4-F#8-30 RT: 0.03-0.11 AV: 23
T: FTMS {1,1} + p ESI Full ms [100.00-2000.00]
m/z= 345.57-429.38

m/z	Intensity	Relative	Theo. Mass	Delta (ppm)	RDB equiv.	Composition
392.14058	56455024.0	100.00	392.14050	0.22	14.5	$C_{22} H_{19} O_3 N_3 F$

Compound **5q**_Mass

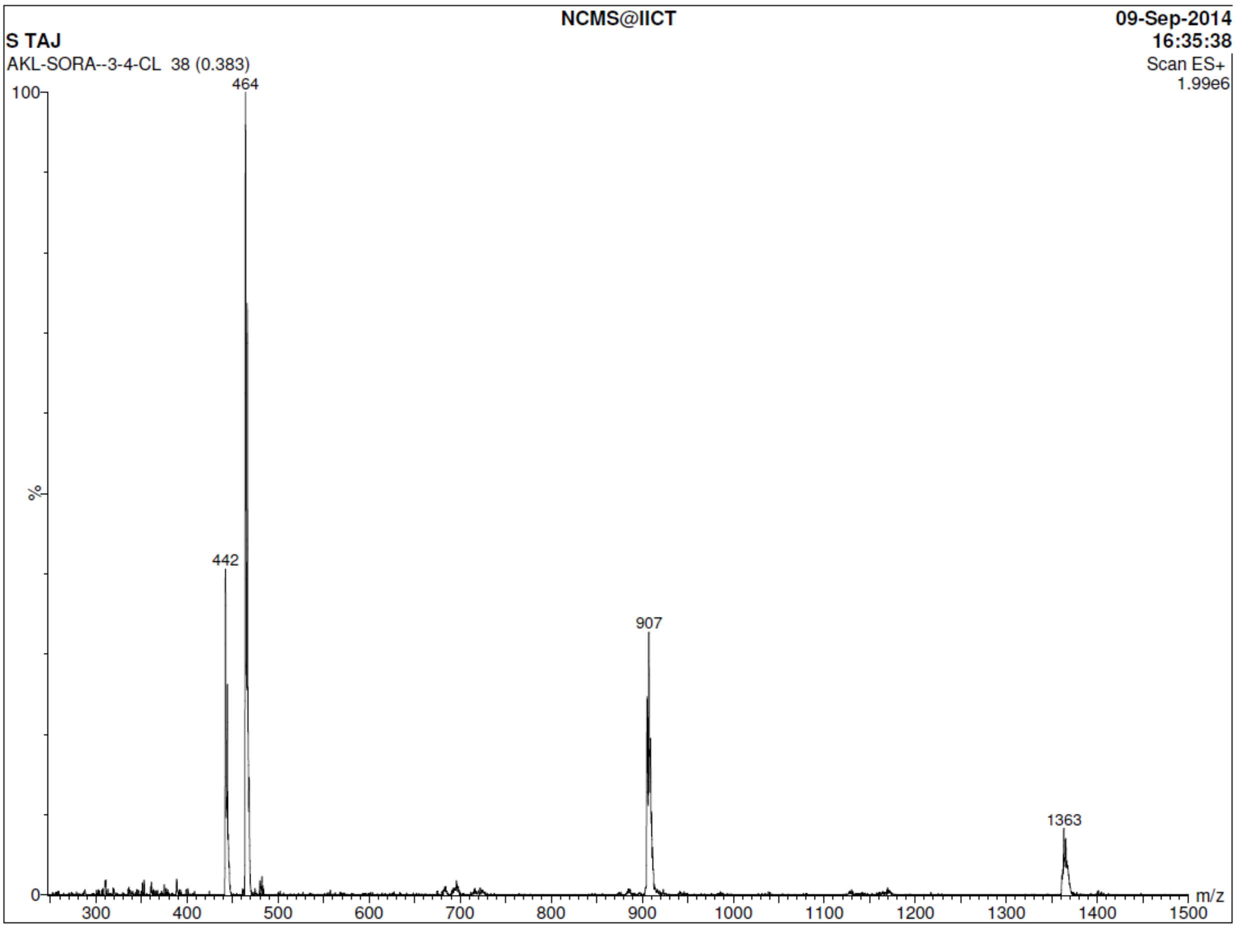

Compound **5q**_HRMS

National Centre for Mass Spectrometry
CSIR-Indian Institute of Chemical Technology

File Name AKL-SORA-3-4-CL
Sample Name
Sample ID SHAIK-TAJ
Date and Time 16-09-14 21:34:29

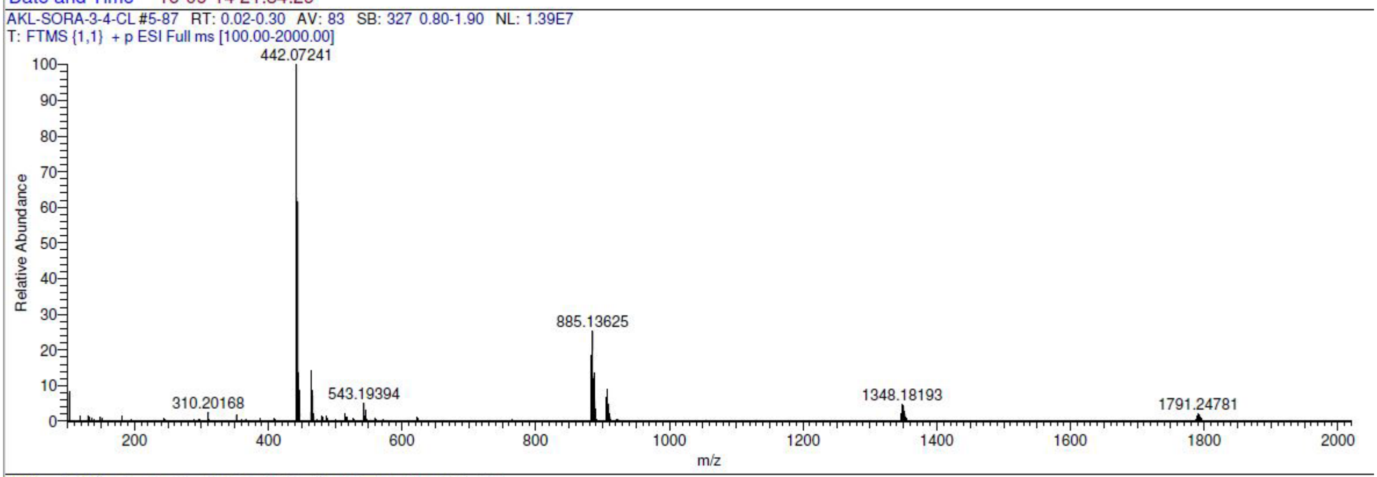

AKL-SORA-3-4-CL#8-30 RT: 0.03-0.11 AV: 23
T: FTMS {1,1} + p ESI Full ms [100.00-2000.00]

m/z	Intensity	Relative	Theo. Mass	Delta (ppm)	RDB equiv.	Composition
442.07266	14034828.0	100.00	442.07197	1.55	14.5	$C_{22}H_{18}O_3N_3Cl_2$

Compound **5r**_Mass

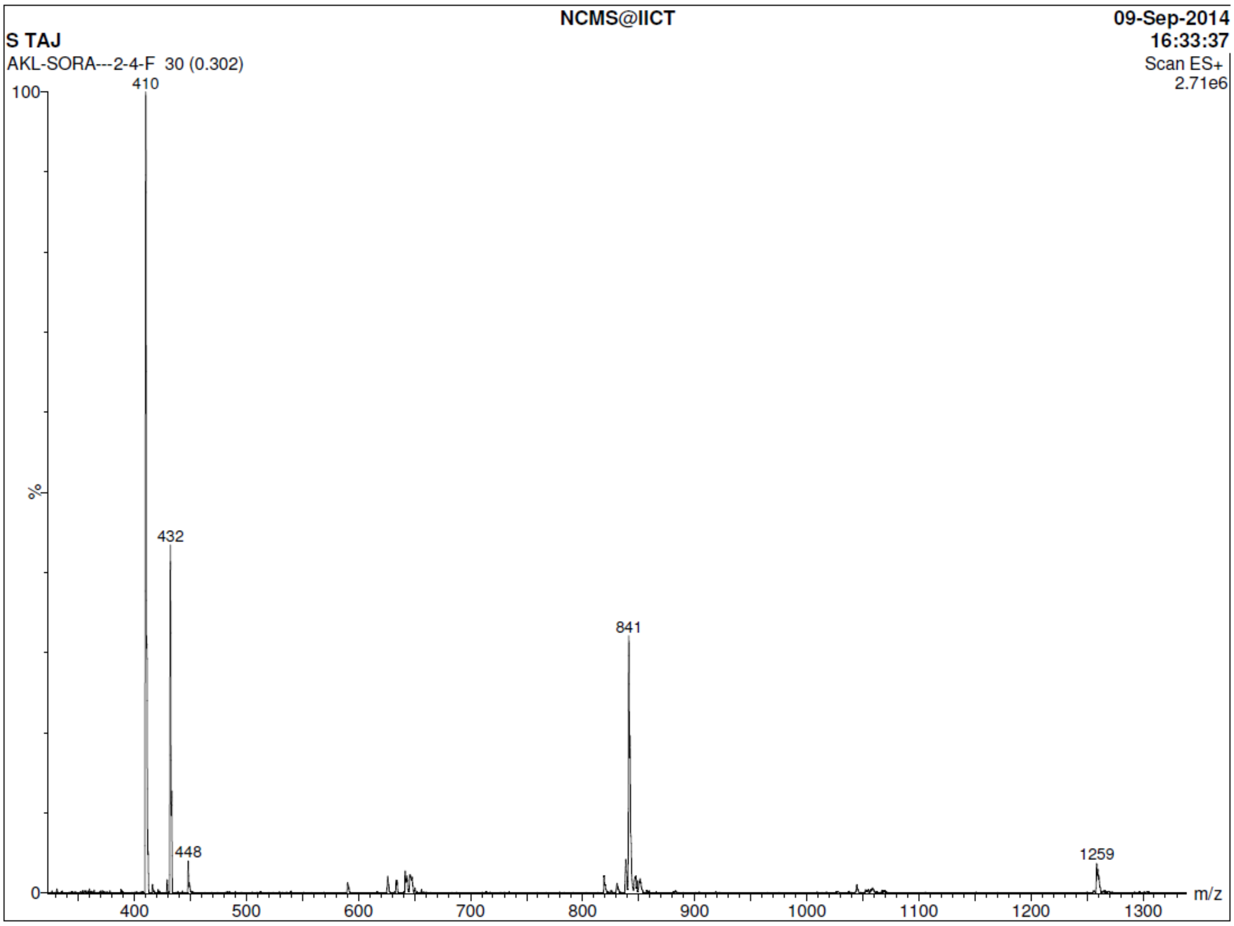

Compound **5r**_HRMS

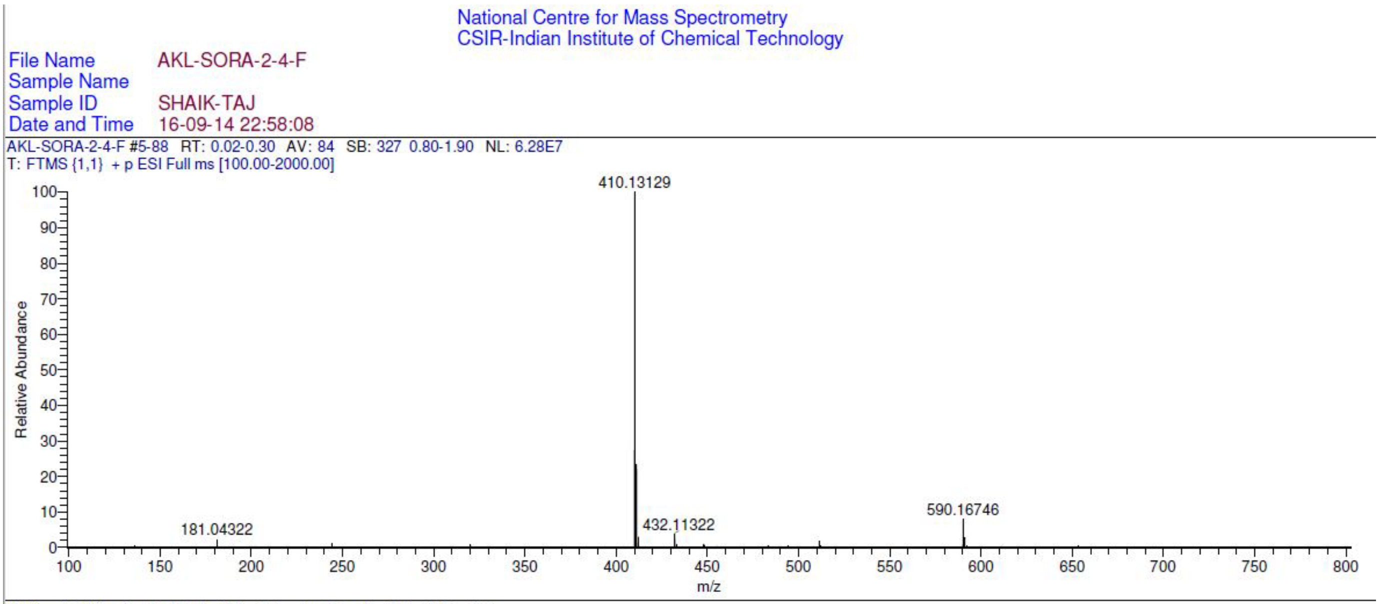

AKL-SORA-2-4-F#8-30 RT: 0.03-0.11 AV: 23
T: FTMS {1,1} + p ESI Full ms [100.00-2000.00]
m/z= 391.49-452.99

m/z	Intensity	Relative	Theo. Mass	Delta (ppm)	RDB equiv.	Composition
410.13111	48726796.0	100.00	410.13107	0.08	14.5	$C_{22}H_{18}O_3N_3F_2$

Compound **5s**_Mass

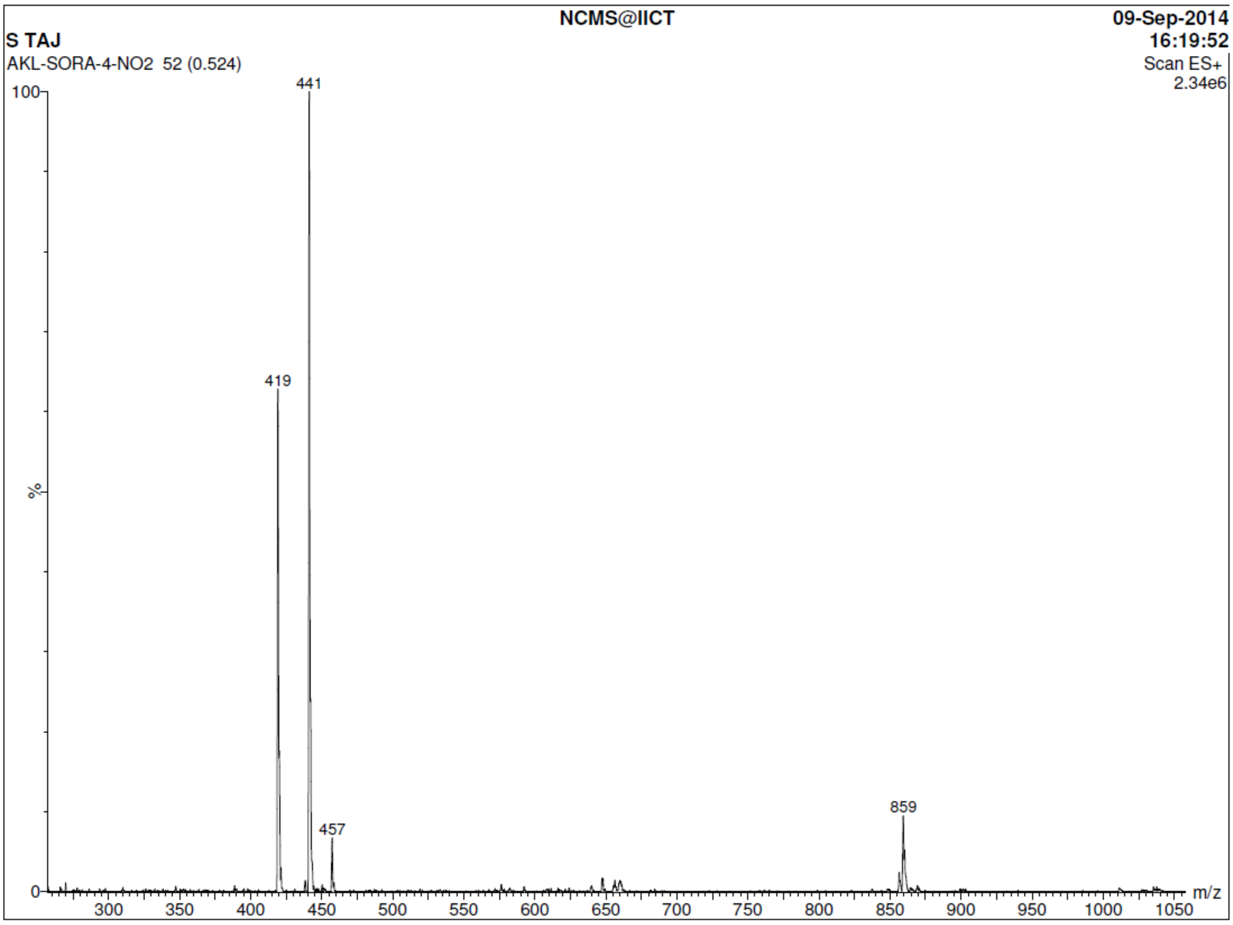

Compound **5s**_HRMS

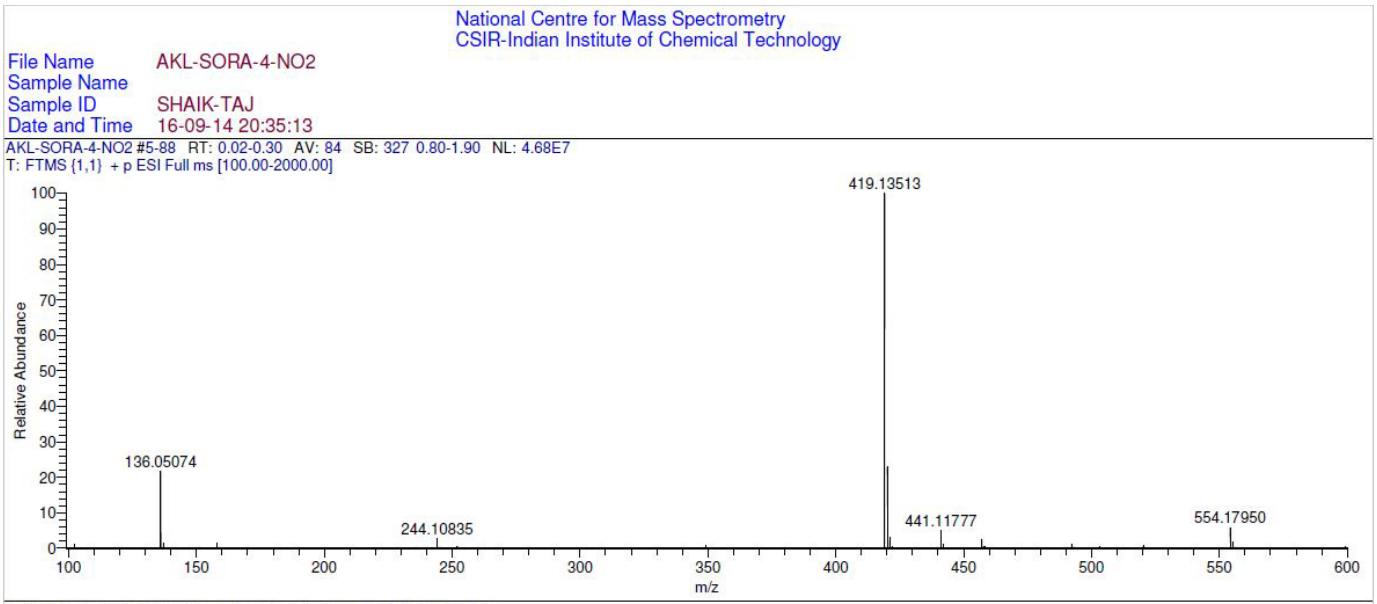

AKL-SORA-4-NO2#8-30 RT: 0.03-0.11 AV: 23
T: FTMS {1,1} + p ESI Full ms [100.00-2000.00]
m/z= 396.79-438.31

m/z	Intensity	Relative	Theo. Mass	Delta (ppm)	RDB equiv.	Composition
419.13513	37396996.0	100.00	419.13500	0.33	15.5	$C_{22} H_{19} O_5 N_4$
